# Taming a Blue Gas—Structure and Chemistry of Hexafluorothioacetone

**DOI:** 10.1002/anie.1044664

**Published:** 2026-07-16

**Authors:** Hennes Günther, Antti J. Karttunen, Xiulan Xie, Frank Tambornino

**Affiliations:** ^1^ Department of Chemistry Philipps University of Marburg Marburg Germany; ^2^ Department of Chemistry and Materials Science Aalto University Aalto Finland

**Keywords:** bonding analysis, complex chemistry, DFT, nuclear magnetic resonance, thioketone

## Abstract

Hexafluorothioacetone (**1**), a blue gas at r.t. and unusually prone to dimerization, was synthesized and characterized by single‑crystal x‑ray diffraction, NMR spectroscopy, and density functional theory (DFT) calculations. The C═S bond lengths in **1** were determined to be 1.583–1.595 Å, representing the shortest C═S bond lengths reported for a non‐cumulene system. DFT calculations indicate an only mildly polar covalent C═S bond. Furthermore, due to the strongly electron withdrawing CF_3_ groups, the polarity of the C═S bond is reversed to C^δ–^═S^δ+^, running contrary to their electronegativities. The ^13^C NMR spectrum of neat **1** collected at 193 K shows resonances at 205.4 ppm (^2^ *J*_CF_ = 37.7 Hz) and 116.7 ppm (^1^ *J*_CF_ = 278.7 Hz), with the thiocarbonyl resonance shifted unusually far up‐field, further underscoring the inverted polarity of the C═S bond. Nickel complexes with **1** as side‐on ligand (η^2^) and ancillary ligands (COD, pyridine, TMEDA) were obtained, in which the nickel atom adopts a pseudo square‑planar geometry. The C–S bond lengths in these complexes (1.735–1.762 Å) lie between typical single (1.78 Å) and double (1.61 Å) bonds.

## Graphical abstract

Hexafluorothioacetone (**1**), a blue gas at r.t., was synthesized by flash vacuum pyrolysis of its cyclic dimer at 660°C. We discuss fundamental properties of elusive **1**, including single‐crystal structure and nuclear magnetic resonance spectroscopy, despite its tendency to dimerize even at –74°C. Furthermore, we investigate the bonding situation of **1** in metal complexes [(**1**)Ni(COD)], [(**1**)Ni(Py)_2_] and [(**1**)Ni(TMEDA)]. Experimental data are supported by DFT calculations.

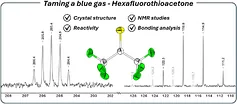

## 1 Introduction

Thioketones and thioaldehydes, the sulfur congeners of ketones and aldehydes, are well known for their disagreeable smell. Thioacetone in particular is highly malodourous and was notoriously reported to contaminate an entire district of Freiburg (Germany) with its foul smell in 1889 [1]. The chemical and physical properties of these reagents are largely determined by their C═S double bond. In comparison to C═O double bonds, the C═S bond exhibits poor orbital overlap between the diffuse 3p orbital of sulfur and the 2p orbital of the carbon atom [2]. Consequently, compounds featuring C═S double bonds exhibit a pronounced tendency to form di‐, oligo‐, and polymers, readily induced by light or heat [2, 3]. However, these systems can be stabilized, either thermodynamically by introducing electron donating groups [2] or kinetically by utilizing sterically demanding frameworks [4]. Further hallmarks of the thiocarbonyl moiety are ^13^C NMR signals in the downfield 250–280 ppm region as well as their coloration from the n–π* absorption band in the same region as visible light [5].

Hexafluorothioacetone, the per fluorinated analogue of thioacetone, is the most thoroughly investigated thioketone in terms of reactivity [6, 7, 8]. This can be attributed to the inability of the C═S double bond to enolize, in addition to its ready preparation via flash vacuum pyrolysis of its cyclic dimer [9]. Reactions of hexafluorothioacetone with nucleophiles reveal the electrophilic character of the C═S double bond. Hexafluorothioacetone is also a highly reactive dienophile, demonstrating exceptional reactivity in Diels‐Alder reactions: The Diels‐Alder reaction with butadiene occurs almost instantaneously at –78°C [7]. It has also been shown that hexafluorothioacetone can act as a ligand for group 10 metals, and NMR studies tentatively indicate the formation of a metallacyclopropane [10, 11, 12]. Although the reactivity of hexafluorothioacetone has been intensely studied, few details of its physical properties have been reported. Its boiling point is 8 °C [13], and the ^19^F‐NMR resonance shows up at 68 ppm (with respect to CCl_3_F) [9]. Both IR and RAMAN spectra in argon matrix have been reported with characteristic bands at 1300–1350 cm^−1^ [5].

In this study we fill the knowledge gap focusing on the structural and spectroscopic properties of hexafluorothioacetone. We report the elusive single‐crystal structure of blue hexafluorothioacetone along with its cyclic dimer. In addition, we investigated the coordination properties of hexafluorothioacetone by preparing a series of nickel complexes. The influence of different ancillary ligands on the binding mode of hexafluorothioacetone toward the nickel atom is discussed on the basis of NMR spectroscopy and SC‐XRD. All experimental data is supported by quantum chemical calculations with density functional theory (DFT).

## 2 Results and Discussion

### 2.1 Syntheses

Hexafluorothioacetone (**1**) was synthesized following the literature protocol [5]. Flash vacuum pyrolysis (FVP) of 2,2,4,4‐tetrakis(trifluormethyl)‐1,3‐dithietan (**2**) at 933 K yields **1** as a deep blue solid which was collected in a cold trap at 77 K (Scheme 1, see Supporting Information for experimental details). Since, **1** can dimerize to reform the starting material **2** at temperatures as low as 195 K, **1** is best stored at 77 K.

**SCHEME 1:**
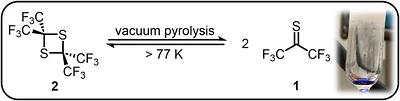
Equilibrium between **1** and **2** (scheme on the left) and picture of **1** in a Schlenk flask right after isolation (image on the right).

To further investigate the thermodynamics of this reversible reaction we performed quantum chemical calculations (DFT‐PBE0/def2‐TZVP level of theory, see Supporting Information). The reaction shown in Scheme 1 is thermodynamically unfavorable at 300 K (Δ*G* = +83 kJ/mol). The reaction eventually becomes thermodynamically favorable at temperatures higher than 700 K (Δ*G* = –19 kJ/mol at 800 K). These results are in line with experimental observations and the high required reaction temperature for the synthesis of **1**.

Complexes of nickel [10], palladium [11], and platinum [12] bearing **1** as a ligand have been described by treating the respective metal precursor (oxidation state ±0) with **2** yielding the metal complexes bearing coordinated **1** (η^2^). It is particularly noteworthy that in all reported reactions the conditions are mild (0–30°C), which stands in sharp contrast to the synthesis of neat **1** (pyrolysis temperature of 933 K, vide supra). We chose to synthesize a series of nickel complexes of general composition of [(**1**)Ni(*L*)_2_] (**3**–**5**) to examine the structural and spectroscopic influence of ***L*** in these complexes (see Scheme 2). For ***L*** we chose cyclooctadiene (COD) since [(**1**)Ni(COD)] serves as precursor for derivatives [10]. We further chose pyridine (Py) for its σ‐donor and weak π‐acceptor properties and tetramethyl ethylenediamine (TMEDA) as a pure σ‐donor. Moreover, DFT calculations show that the formation of [(**1**)Ni(COD)] from Ni(COD)_2_ and **2** is thermodynamically strongly favored by Δ*G* = –105 kJ/mol (gas‐phase calculation at 300 K). Coordination of **1** toward the nickel atom in [(**1**)Ni(COD)] leads to significant weakening of the COD ligand and the latter is readily replaced by Py and TMEDA. All three complexes show an intense yellow/brown (***L*** = COD) to red color (***L*** = Py; ***L*** = TMEDA). The compounds are obtained in moderate yields (33%–68%, see Supporting Information for experimental details).

**SCHEME 2:**
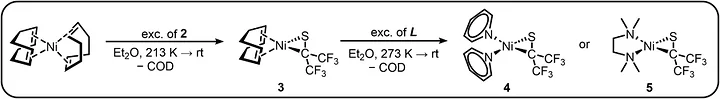
Modified literature synthesis [10] of **3** and ligand replacement yielding derivates **4** (***L*** = pyridine) and **5** (***L*** = TMEDA).

### 2.2 Single‐crystal X‐Ray Studies

Due to the strong inductive effects of the CF_3_ groups on the CS carbon atom, a shortening of the C═S double bond in **1** can be expected. A previous study predicted the C═S bond length in **1** to be 1.582 Å at the HF/6‐311G(d) level of theory [5]. This distance is shorter than the theoretical value of a C═S double bond derived from self‐consistent covalent radii (1.61 Å) [14]. Therefore, one prime objective was to gain insight into the molecular structure of **1** in the solid state by single‐crystal x‐ray diffraction. Because **1** is a reactive gas at r.t., with a tendency to dimerize rapidly above 77 K (especially as a neat sample), the elucidation of the crystal structure was challenging. For liquids or gases that are not as temperature sensitive as **1**, it usually suffices to fill a capillary, flame‐seal it and transfer it to the diffractometer. With this protocol, the sample temperature is at room temperature (in between preparation of the capillary and measurement) with the capillary withstanding the pressure of the enclosed gas. Subsequently, crystals can be grown from melt by manipulation of the temperature of the cooling stream. When trying to get a crystal structure of **1** this procedure resulted in datasets consistent with the dimer **2**, even if visually the sample still exhibited its typical blue color. Clearly, the procedure had to be modified. It was paramount to keep the temperature as low as possible and thus to avoid dimerization. Accordingly, after FVP **1** is first collected in a cold trap kept at 77 K. Next, a small amount of sample was condensed into a capillary (outer diameter 0.3 mm, wall thickness 0.01 mm, Lindemann Spezialglas) that was also cooled to 77 K with liquid N_2_ before it was flame sealed with an arc lighter. The lower part of the capillary was already glued into the capillary holder whose weight, upon flame‐sealing, let the capillary drop into the beaker filled with liquid N_2_. Thus, the sample was maintained at low temperatures throughout the procedure. The capillary was then transferred onto the diffractometer with the cooling stream pre‐set to 100 K. Consequently, the sample was only marginally exposed to r.t., keeping the temperature for most of the time at 77 K. The full procedure with pictures can be found in the Supporting Information. Test measurements revealed a polycrystalline sample with typical “powder rings” (see Figure S33). To grow a single‐crystal in the capillary, the sample was warmed at a rate of 80 K/h and simultaneously observed through the attached microscope to find its melting point. At 133 K, **1** began to melt. Afterwards, the sample was cooled from 140 to 100 K with a cooling rate of 5 K/h. Test measurements indicated the growth of multiple individual crystals with well separated reflection maxima. We were able to index a cell with cell parameters different from the dimer **2** (see Supporting Information for details). Subsequently, a full data set was collected at 100 K.

**1** crystallizes in the monoclinic crystal system with space group *C*2/*c* (No. 15, *a* = 11.6099(5) Å, *b* = 6.3249(2) Å, *c* = 32.3849(14) Å, *β* = 91.893(4)°, *V* = 2376.77(16) Å^3^), two independent molecules in the asymmetric unit and 16 molecules in the unit cell (*Z* = 16) (Figure 1, left). All atoms of both molecules occupy the general position 8*f*. Whereas, one molecule is fully ordered, the other shows rotational disorder in the CF_3_ groups (see Figure S39).

**FIGURE 1:**
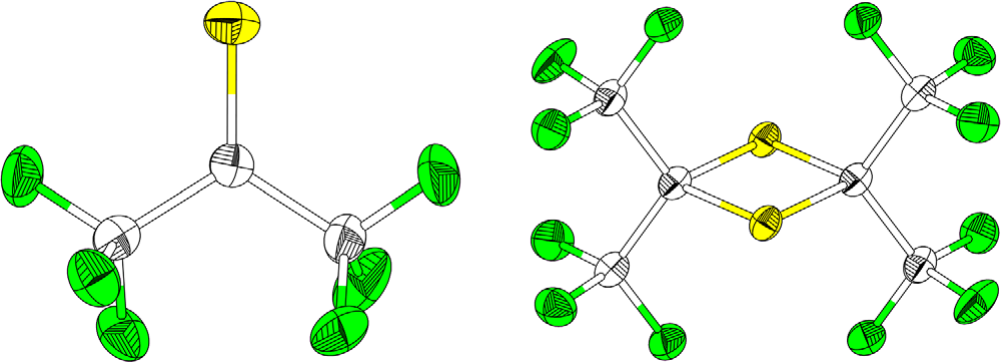
Molecular structure of one molecule of **1** (left) and **2** (right) in the single‐crystal drawn with 30% (**1**) and 50% (**2**) displacement ellipsoids at 100 K. Color code: C white, F green, S yellow.

The C═S bond lengths in **1** are 1.583(5) Å and 1.595(5) Å (for both crystallographically independent moieties) and thus are shorter than the theoretical value of a C═S double bond (1.61 Å) [14]. The C–C bond lengths are 1.509(7)–1.52(6) Å and thus in the typical range of C–C single bonds (1.50 Å) [14]. Bond angles of ∢(S–C–C) and ∢(C–C–C) are 121.8(4)–123.4(4)° and 114.8(4), 115.0(4)°, respectively. To get deeper insight into the bonding and find indication for the source of the shortness of the C═S bond, we performed quantum chemical calculations. The molecular structure of **1** was optimized in the gas phase using quantum chemical calculations (DFT‐PBE0/def2‐TZVP level of theory, *C*_2_ point group, see Supporting Information for computational details) [15, 16, 17]. The bond lengths and angles of the optimized gas‐phase molecular structure are very close to those observed in the crystal structure: The C═S bond length is 1.59 Å and the C–C bond lengths are 1.53 Å. The bond angles ∢(S–C–C) and ∢(C–C–C) are 122.7° and 114.7°, respectively.

We analyzed the chemical bonding in **1**–**5** with the aid of Intrinsic Bond Orbitals (IBOs) [18]. IBOs can be considered as a physically well‐defined form of localized molecular orbitals designed for chemical interpretation. A full listing of the IBOs of **1**–**5** is available in the Supporting Information. Figure 2, shows the Intrinsic Atomic Orbital (IAO) partial charges and IBOs of **1**. The S atom and central C atom possess partial charges of +0.16 *e* and –0.19 *e*, respectively. In its hydrogen analogue thioacetone these values are –0.09 *e* and –0.01 *e* (see Figure S57 for details). This charge inversion is due to the high group electronegativity of the CF_3_ groups which manifests itself by remarkable reactivity. Nucleophilic [SO_3_H]^–^ reacts with the S atom of the C═S bond in **1** forming [H(CF_3_)_2_CSSO_3_]^–^ [6]. The IBO analysis shows a C–S σ bond (Figure 2b) and C–S π bond (Figure 2c), as expected for a C═S double bond. The percentages show the contribution of each atom to an IBO. In a purely covalent two‐atomic bond, each atom would contribute 50%. If the percentages do not add up to 100%, other atoms have small contributions of less than 2%. The plotted IBO isosurfaces enclose 80% of the total electron density of the IBO. In the case of **2** (see Supporting Information), all four C–S bonds are slightly polar σ bonds, where the C atom shows 53% contribution.

**FIGURE 2:**
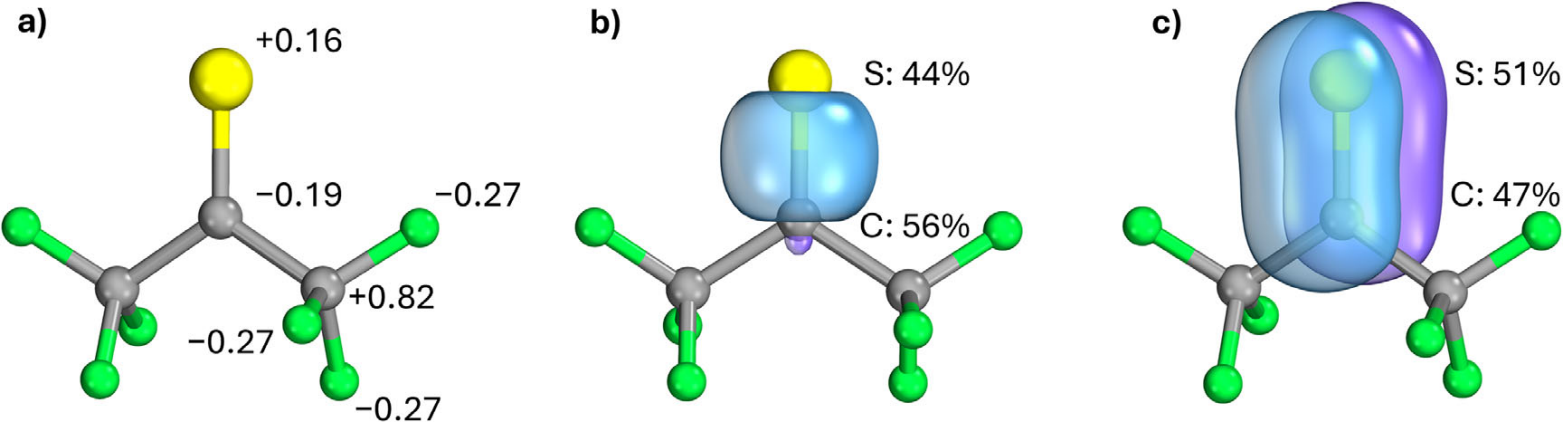
(a) Partial charges (*e*) of **1** from Intrinsic Atomic Orbitals analysis. (b) Intrinsic bond orbital (IBO) corresponding to C–S σ bond in **1**. (c) IBO corresponding to C–S π bond in **1**. Color code as in Figure 1. If the percentages do not add up to 100%, other atoms have small contributions of less than 2%. The plotted IBO isosurfaces enclose 80% of the total electron density of the IBO.

For structural comparison, we also determined the single‐crystal structure of **2** (see Supporting Information for additional information). **2** crystallizes in the monoclinic crystal system with space group *P*2_1_/*n* (No. 14, *a* = 6.6082(4) Å, *b* = 9.7165(4) Å, *c* = 8.5973(5) Å, *β* = 108.372(5)°, and *V* = 523.88(5) Å^3^), with one independent molecule in the asymmetric unit and **2** molecules in the unit cell (*Z* = 2). The C–S bond lengths are considerably longer than in **1** with values of 1.825(2) and 1.830(2) Å. These correspond to a slightly elongated C–S single bond (1.78 Å) [14]. Furthermore, a less pronounced elongation of the C–C bond lengths (1.542(3)–1.545(2) Å) is observed. Secondly the ∢(C–C–C) angles of **2** are widened to 111.8(1)°. The 1,3‐dithietane ring is strained with angles of 84.75(8)° and 95.25(8) for ∢(C–S–C) and ∢(S–C–S), respectively. DFT‐PBE0 structural optimization of **2** in the gas phase (point group *C*_2_ *_h_*) results in bond lengths and angles in close agreement with the crystal structure. The C–S and C–C bond lengths are 1.82 and 1.55 Å, respectively. The ∢(S–C–C) bond angles are 111.1–113.9° and the ∢(C–C–C) bond angles are 111.2°.

The C═S double bond length in “classic aliphatic” compounds, for example, thiocamphor [19] (1.617(2) Å) is consistent with the theoretical length of a C═S double bond (1.61 Å) [14], however, significantly longer than in **1** (1.583(5) Å). To the best of our knowledge, **1** exhibits the shortest C═S double bond of a thioketone based on single‐crystal structure data reported in the literature. Only the C═S double bonds in crystalline CS_2_ [20] are shorter than in **1**, exhibiting a value of 1.560(3) Å, which is possibly attributable to the specific allene‐type electron configuration. The IBO analysis of CS_2_ shows differences between **1** and CS_2_. **1** has a polar C–S σ bond similar to the C–S σ bond in CS_2_ (Figure S58). However, CS_2_ shows two highly polar C–S π bonds (S contribution 74%) and one S lone pair, while **1** shows only one such bond (S contribution 51%) and two S lone pairs. When gas‐phase electron diffraction data are considered, the bond length of CSF_2_ is approximately equal to that of **1** (Table 1 and Figure 3) [21].

**TABLE 1:** C═S bond lengths (in Å) of **1** and selected compounds in literature.

Compound	C═S bond length / Å	Reference
**1**	1.583(5)	This work
Thiocamphor^b^	1.617(2)	[19]
CS_2_ ^a^	1.560(3)	[20]
CSF_2_ ^c^	1.585(4)	[21]
COS ^d^	1.51(3)	[22]

^a^ SCXRD (133 K).

^b^ PXRD (90 K).

^c^ gas phase electron diffraction (183.15 K).

^d^ neutron powder diffraction (90 K).

Upon coordination to a metal atom, the electronic structure of a ligand is altered, most notably in complexes where π‐bonding interactions are significant. Since syntheses of nickel complexes of **1** have already been described in the literature, however, without structural data, we aimed to elucidate how its bonding situation is modified upon metal coordination. Single‐crystals of **3**, **4** and **5** were obtained by simple solvent evaporation in vacuo (see SI for details). In all three structures, the nickel atom adopts a pseudo‐square‐planar geometry with side‐on (η^2^) coordination of **1** together with two ancillary ligands (COD in **3**, Py in **4**, and TMEDA in **5**). The C–S bond lengths are 1.735(2) Å and 1.738(6) Å, 1.751(2) Å, 1.762(5) Å, for **3**, **4** and **5**, respectively (see Tables S2 and S3). All three bond lengths fall in between canonical C–S single (1.78 Å) and C═S double bonds (1.61 Å) [14]. DFT‐PBE0 optimizations reproduce this trend, yielding C–S distances of 1.72, 1.74, and 1.75 Å in the gas phase. The bonding situation is best described within the Dewar–Chatt–Duncanson [23] framework: given the strong electron deficiency of **1**, the coordination is more consistent with a metallacyclopropane‐like structure than with a classical metal–olefin interaction (Figure 4).

**FIGURE 3:**
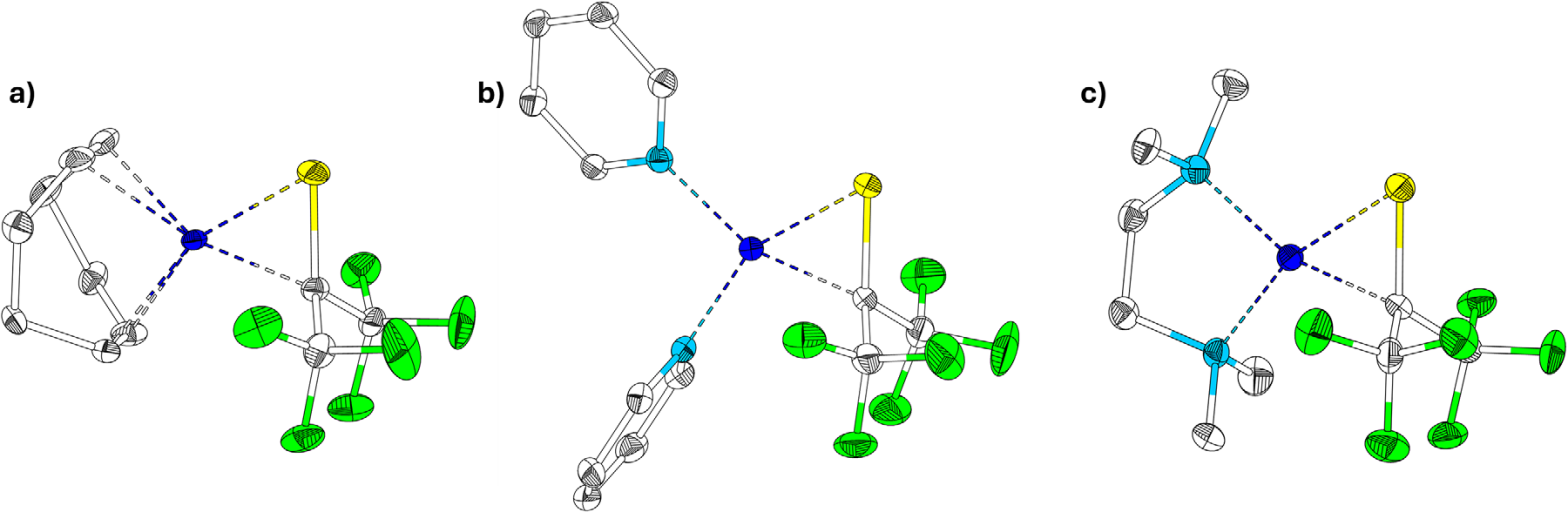
Molecular structure of **3** (a), **4** (b), and **5** (c) in the single‐crystal drawn with 50% displacement ellipsoids at 100 K. Hydrogen atoms are omitted for clarity. Color code: C white, F green, S yellow, N light blue, Ni dark blue.

**FIGURE 4:**
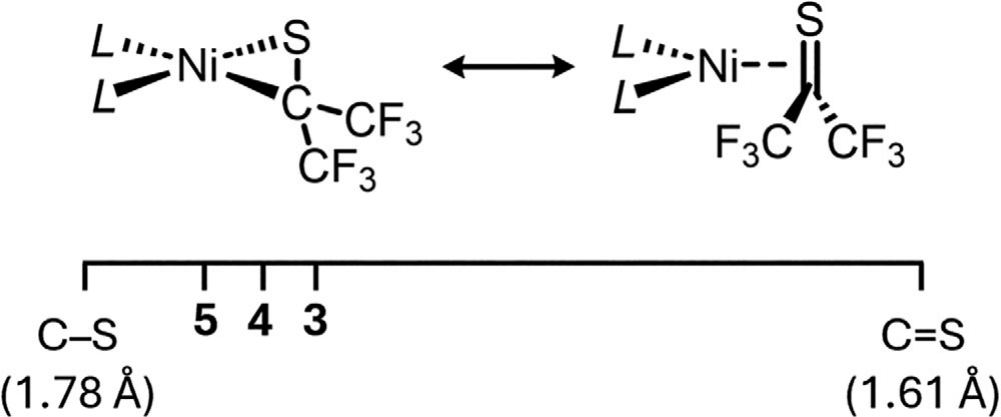
Lewis representation of the mesomeric boundary structures of the bonding situation in **3**, **4**, and **5**: Metallacyclopropane form (left) and metal‐olefin (right).

This interpretation is supported by the C–S bond lengths in complexes **3**–**5**, which approach those of a C–S single bond. Notably, the extent of bond shortening correlates with the π‐acceptor strength of the ancillary ligand ***L***. Weaker π‐acceptors lead to longer C–S distances. This trend indicates that enhanced π‐back bonding reduces electron density at the nickel atom and slightly increases the structural contribution of the classical metal–olefin bonding mode.

Figure 5 illustrates the IBOs of **3**. The IBO analysis shows one σ‐type single bond between C and S bond. Compared to **1**, the partial charge of the S atom changes from +0.16 *e* to –0.19 *e* due to the coordination of the Ni(COD). The partial charges of the central C atom and the Ni atom are –0.41 *e* and +0.67 *e*, respectively. Polar Ni–C and Ni–S bonds are formed between Ni *d*‐orbitals and the *p*‐orbitals of S and C (Ni contribution 30%–33%). A similar bonding pattern is present in **4** and **5**, with the Ni–C and Ni–S bonds becoming even more polar (Ni contribution 27%, see Supporting Information for all data) which is in line with the hypothesis that decreased π‐back bonding leads to greater structural contribution of the metallacyclopropane description.

**FIGURE 5:**
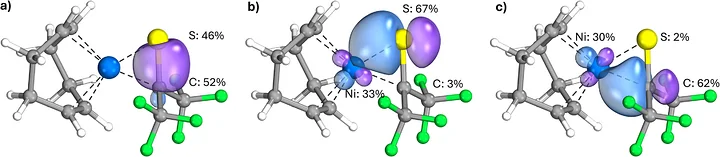
(a) Intrinsic bond orbital (IBO) corresponding to C–S σ bond in **3**. (b) IBO corresponding to Ni–S bond in **3**. (c) IBO corresponding to Ni–C bond in **3**. Color code as in Figure 3. If the percentages do not add up to 100%, other atoms have small contributions of less than 2%. The plotted IBO isosurfaces enclose 80% of the total electron density of the IBO.

### 2.3 NMR Studies

NMR spectra (^1^H, ^13^C and ^19^F) were recorded for all compounds (see Figure 6 for excerpts and Supporting Information for full spectra). To accurately determine multiplicities and coupling constants, a neat sample of **1**, and a sample of **2** in CDCl_3_ were prepared (see Supporting Information for experimental details). As for the determination of the single‐crystal structure, the sample preparation of temperature sensitive **1** proved to be challenging. To collect sensible NMR data, the sample needed to be homogenous, and we chose 193 K as an appropriate measurement temperature. Furthermore, we precooled the probe to 193 K and adjusted our sample to the same temperature before it was guided into the magnet. In this way, we achieved minimal temperature gradient along the NMR tube and were able to obtain excellent signals without shimming. Recording of all three spectra of **1** was completed within just 15 min.

**FIGURE 6:**
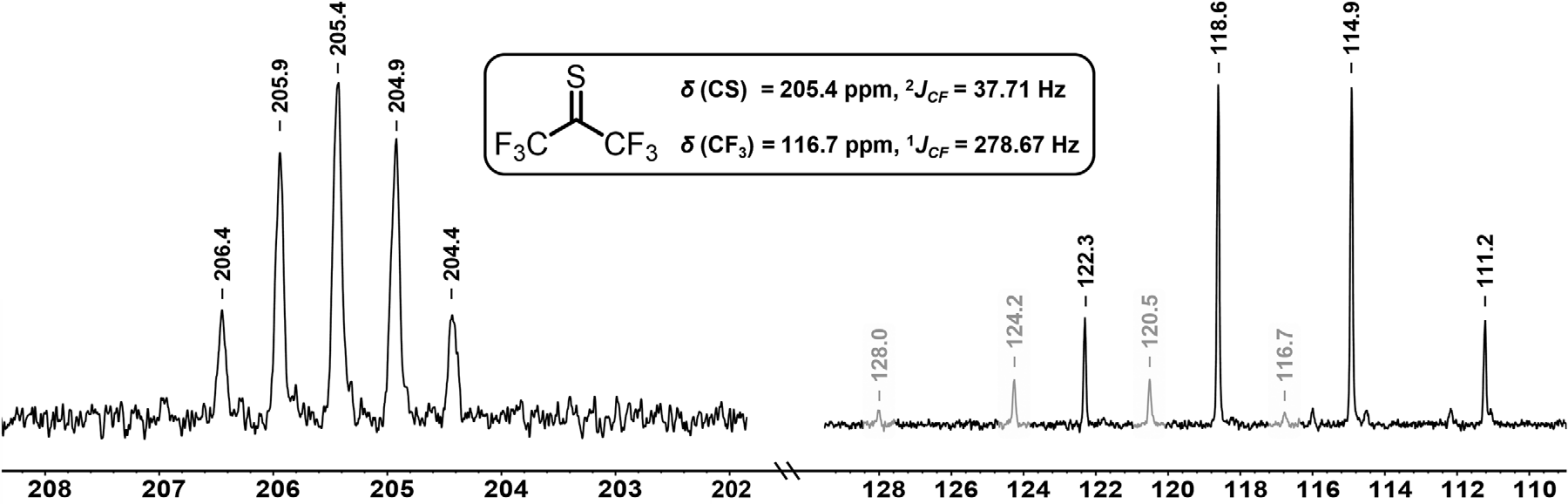
Excerpts of the ^13^C NMR spectrum of 1 collected at 193 K. Signals corresponding to 2 are shown in grey.

Even at 193 K dimerization was observed, however, the concentration of the dimerization product **2** was low. The ^13^C‐NMR spectrum of **1** shows two signals at 205.4 ppm (*pentet*, ^2^ *J_CF_* = 37.71 Hz) and 116.7 ppm (quartet, ^1^ *J_CF _* = 278.67 Hz). Signals of comparatively low intensity stemming from CS_2_ and **2** are also detected. CS_2_ likely originates from decomposition during pyrolysis. For the signal assignment of **1** this does not pose a problem, as the signals can easily be distinguished based on their chemical shift and relative intensity. Although a neat sample was used, the expected septet for the C═S carbon atom was not fully detected with the outermost parts of the septet indistinguishable from the baseline noise. This is likely due to the inherently low signal intensity of the quaternary carbon and the restricted measurement time imposed by the compound's instability. Although the sample was prepared shortly before the NMR experiment, signals of CF_3_ groups of **2** were detected, further underlining the high sensitivity to temperature. Typically, C═S groups are detected at chemical shifts of 250–280 ppm [24] and we were at first surprised by the relatively high field shift of 205.4 ppm. The hydrogen analogue of **1**, thioacetone, shows a signal at 252.7 ppm and is therefore shifted approx. 45 ppm toward low field [24]. An analogous trend is observed in the lighter chalcogen congeners acetone [25] (206.4 ppm) and hexafluoroacetone [26] (172.9 ppm). Apparently, the CF_3_ groups mitigate the strong polarization of the C═S bond toward the sulfur atom, thus a partial reversal in polarity of the CS bond is observed (vide supra). This reversal of the bond polarization was previously described and experimentally verified. The electrophilic center of **1** is not, as usual, located solely on the carbon atom, but also on the sulfur atom. Therefore, in addition to the two classical resonance structures C═S and C^+^–S^–^, they also proposed a third structure featuring a reversed polarization C^−^–S^+^, to illustrate the actual bonding situation in a simplified manner [6]. This hypothesis is supported by the calculated partial charges (C = –0.19 *e*; S = +0.16 *e*, see Figure 2). The coupling constants ^1^ *J_CF_* = 278.67 Hz and ^2^ *J_CF_* = 37.71 Hz are also comparable with those detected in CF_3_CH_2_CF_3_ [27] (^1^ *J_CF_* = 274.50 Hz; ^2^ *J_CF_* = 32.44 Hz).

The ^13^C‐NMR spectrum of **2** in CDCl_3_ shows two signals at 123.5 ppm (quartet, ^1^ *J_CF_* = 283.53 Hz) and 41.9 ppm (*pentet*, ^2^ *J_CF_* = 35.87 Hz). As expected, the CS resonance is shifted up‐field relative to **1**, consistent with the “sp^3^” configuration of the CS carbon atom. However, the chemical shift of the CF_3_ groups and respective coupling constants of **1** and **2** show only a very small deviation. Its selenium analogue 2,2,4,4‐tetrakis(trifluor‐methyl)‐1,3‐diselenetane [28] shows similar resonances and coupling constants for the CF_3_ moiety (123.2 ppm, ^1^ *J_CF_* = 280 Hz). Because of the heavier selenium, a high field shift is observed for the 1,3‐diselenetans ring; however, the coupling constant is comparable to **2** (15.0 ppm, ^2^ *J_CF_* = 37 Hz).

Particularly noteworthy is the chemical shift of the CS moiety of **1** as ligand in **3**, **4,** and **5**. Signals of the CS moiety are detected at 59.8 (**3**), 46.3 (**4**), and 41.7 ppm (**5**). Here, similarities to the trend observed in the CS bond lengths can be identified. On the one hand, all three chemical shifts lie close to that characteristic of an “sp^3^” configured CS moiety, supporting the dominance of a metallacyclopropane‐type structure (Figure 4). On the other hand, the chemical shift correlates with the π‐acceptor strength of the respective ligand ***L***: The stronger the π‐acceptor properties of ***L***, the further the signal of the CS carbon is shifted toward down field. When compared to the results of our IBO analysis we observed that the high field shift of the complexes from **3** to **5** is associated with the progressively more polar C–Ni bond (62%, 66%, and 67% for the C bonding contribution in **3**, **4,** and **5**, respectively).

## 3 Conclusion

Vacuum pyrolysis of 2,2,4,4‐Tetrakis(trifluoromethyl)‐1,3‐dithietan at 933 K yields the blue gas hexafluorothioacetone (**1**). We were able to investigate its single‐crystal structure and NMR signatures despite its pronounced tendency to dimerize. In the single‐crystal structure of hexafluorothioacetone the C═S double bond is with 1.583(5) Å the shortest reported in the literature for a thioketone and significantly shorter than a theoretical C═S double bond (1.61 Å). IBO and partial charge analysis reveal the presence of a weakly polar CS double bond with inverted polarity: Partial charges of +0.16 *e* and –0.19 *e* for S and C, respectively. Both phenomena can be traced back to the strong inductive influence of the CF_3_ groups.

The [2+2] cycloreversion of the dimer **2** becomes thermodynamically favorable only at high temperatures. However, in the presence of [Ni(COD)_2_] cycloreversion of **2** becomes exergonic with Δ*G*  = –105 kJ/mol even at 300 K, forming **3**. COD can subsequently be substituted with pyridine and TMEDA. In all three complexes, the nickel atom adopts a pseudo‐square‐planar geometry with side‐on (η^2^) coordination of **1** together with two ancillary ligands. The CS bond lengths in **3**, **4**, and **5** are significantly longer than in neat **1** and only slightly shorter than a CS single bond (1.78 Å). The Dewar‐Chatt‐Duncanson model suggests a metallacyclopropane‐like structure. Support for this interpretation is provided by IBO analysis, which shows polar single bonds between hexafluorothioacetone and the nickel atom.

The ^13^C‐NMR spectrum of neat **1** shows signals at 205.4 and 116.7 ppm. The chemical shift of the thiocarbonyl group at 205.4 ppm is the most up‐field resonance for a thioketone reported to date. The ^13^C NMR signals of the metal complexes appear at 59.8 (**3**), 46.3 (**4**), and 41.7 ppm (**5**). These up‐field shifts are consistent with the metallacyclopropane bonding model, in which the ligand adopts a “sp^3^”‐like configuration. Studies of compounds closely related to hexafluorothioacetone are currently in progress.

## Author Contributions

**Hennes Günther**: investigation, data curation, visualization, and writing – original draft. **Antti J. Karttunen**: writing – original draft, methodology, software, and data curation. **Xiulan Xie**: writing – original draft, methodology, and data curation. **Frank Tambornino**: conceptualization, funding acquisition, writing – review and editing, project administration, and supervision.

## Conflicts of Interest

The authors declare no conflicts of interest.

## Supporting information



The authors have cited additional references within the Supporting Information [29, 30, 31, 32, 33, 34, 35, 36, 37, 38, 39, 40, 41].
**Supporting File 1**: anie73558‐sup‐0001‐SuppMat.pdf.

**Supporting File 2**: anie73558‐sup‐0002‐cif.zip.

## Data Availability

The data that supports the findings of this study are available in the supplementary material of this article
