# Supplementary material for: Taming a Blue Gas—Structure and Chemistry of Hexafluorothioacetone
Source: Angew Chem Int Ed Engl. 2026 Jul 16;65(40):e1044664. doi: 10.1002/anie.1044664 (PMC13618283; doi:10.1002/anie.1044664)
Supplement: Supplementary file 1 [file ANIE-65-e1044664-s002.pdf]

**Electronic Supporting Information (ESI)**

# Taming a blue gas – Structure and chemistry of hexafluorothioacetone

Hennes Günther[a], Antti J. Karttunen[b], Xiulan Xie[a], and Frank Tambornino[a]*

[a] Department of Chemistry
Philipps University of Marburg
Hans-Meerwein-Str. 4, D-35043 Marburg, Germany
E-mail: frank.tambornino@chemie.uni-marburg.de

[b] Department of Chemistry and Materials Science,
Aalto University
Kemistintie 1, FI-00076 Aalto, Finland

**Contents**

**Inhalt**

1. Experimental section ..... 3
  1.1 General remarks ..... 3
  1.2 Additional characterization techniques: ..... 3
  1.3 Synthesis of hexafluorothioacetone (1) ..... 5
  1.4 Synthesis of cycloocta-1,5-diene(hexafluorothioacetone)nickel (3) ..... 8
  1.5 Synthesis of bispyridine(hexafluorothioacetone)nickel (4) ..... 11
  1.5 Synthesis of bistetramethylendiamine(hexafluorothioacetone)nickel (5) ..... 18
  1.6 NMR spectra of 2,2,4,4-Tetrakis(trifluormethyl)-1,3-dithietan (2) ..... 22
2. Crystallographic data ..... 24
  2.1 Experimental details on single crystal X-ray structure determination of 1 ..... 25
  2.2 Single crystal X-ray data collection and refinement parameters ..... 28
  2.2 Hirshfeld surface and fingerprint plots for 1 ..... 29
  2.3 Additional Pictures ..... 31
    2.3.1 Additional Pictures of 1 ..... 31
    2.3.2 Additional Pictures of 2 (100 K) ..... 33
    2.3.3 Additional Pictures of 3 (alpha) ..... 35
    2.3.4 Additional Pictures of 4 ..... 36
    2.3.5 Additional Pictures of 5 ..... 37
3. Additional tables ..... 38
4. Computational details ..... 39
  4.1 Intrinsic Bond Orbitals for studied systems ..... 39
  4.2 Optimized molecular structures in XYZ format ..... 46

# 1. Experimental section

## 1.1 General remarks

Compounds:

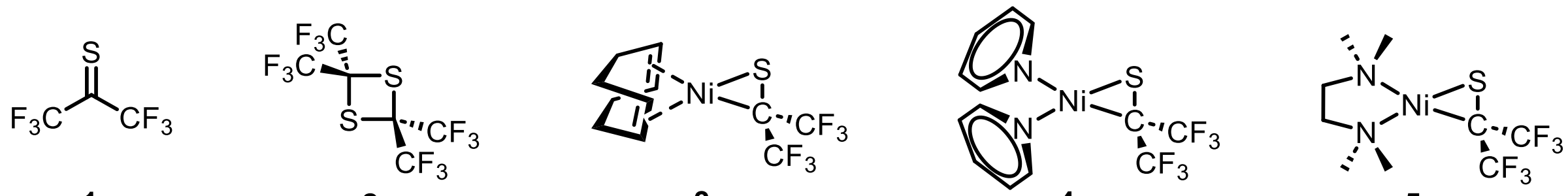

**1** **2** **3** **4** **5**

*General synthetic methods.* All reactions and manipulations were performed under an inert atmosphere of argon using standard Schlenk-line or glovebox techniques (MBraun UNIlab glovebox, maintained at < 0.1 ppm $H_2O$ and < 0.1 ppm $O_2$).

Solvents were dried according to literature.[1] $CDCl_3$ (Eurisotop, 99.8 %) and $C_6D_6$ (Eurisotop, 99.5%) were degassed and stored over molecular sieve (4 Å) prior to use. Compound **2** was kindly donated by Prof. Sundermeyer; its synthesis was carried out according to literature.[2] [Ni(COD)$_2$] was synthesized according to literature.[3]

## 1.2 Additional characterization techniques:

### IR Spectroscopy

IR spectra were recorded on a Bruker Alpha FT-IR spectrometer equipped with a diamond ATR unit mounted. The spectrometer was housed in a glovebox (MBraun UNIlab glovebox, maintained at < 0.1 ppm $H_2O$ and < 0.1 ppm $O_2$).

### NMR Spectroscopy

For NMR studies of **1,** blue hexafluorothioacetone was freshly condensed into a NMR tube and sealed under vacuum at −174 °C with a butane torch. The sample was stored at −174 °C in the absence of light until measurement and was slowly allowed to warm to the respective temperature just before measurement. Recording $^{1}H$, $^{19}F$ and $^{13}C$ spectra at temperatures down

to −80 °C was realized on a Bruker 5 mm BBFO probe equipped with devices $LN_2$ heat exchanger, $LN_2$ evaporator and variable temperature control unit. Cooling on the sample is realized by using cold gas evaporated from a reservoir of liquid $N_2$ and the temperature is regulated by the control unit. For measurements on the very sensitive sample of **1**, the probe was precooled to −80 °C and the sample was adjusted to the same temperature before it was guided into the magnet. In this way we realized minimal temperature gradient along the NMR tube and obtained homogenous condition within the sample, therefore succeeded in getting nice resonance signals without shimming.

For NMR experiments on **2**, the sample was neat liquid with trace of $CDCl_3$. For **3**, **4** and **5**, 10–30 mg samples were dissolved in 0.6 mL of either $C_6D_6$, $CD_2Cl_2$, or $CDCl_3$. Different solvents were chosen to avoid signal overlap thus assure unambiguous assignment especially to the $^{13}C$ signals. The $^{1}H$, $^{19}F$ and $^{13}C$ spectra at −80 °C were recorded on a Bruker NEO 300, while $^{1}H$, $^{19}F$, $^{13}C$ and triple resonance $^{13}C\{^{1}H, ^{19}F\}$ spectra at 25 °C were recorded on a Bruker NEO600 with a iProbe TBO BBF/H/D capable of performing decoupling on $^{1}H$ and $^{19}F$ simultaneously.

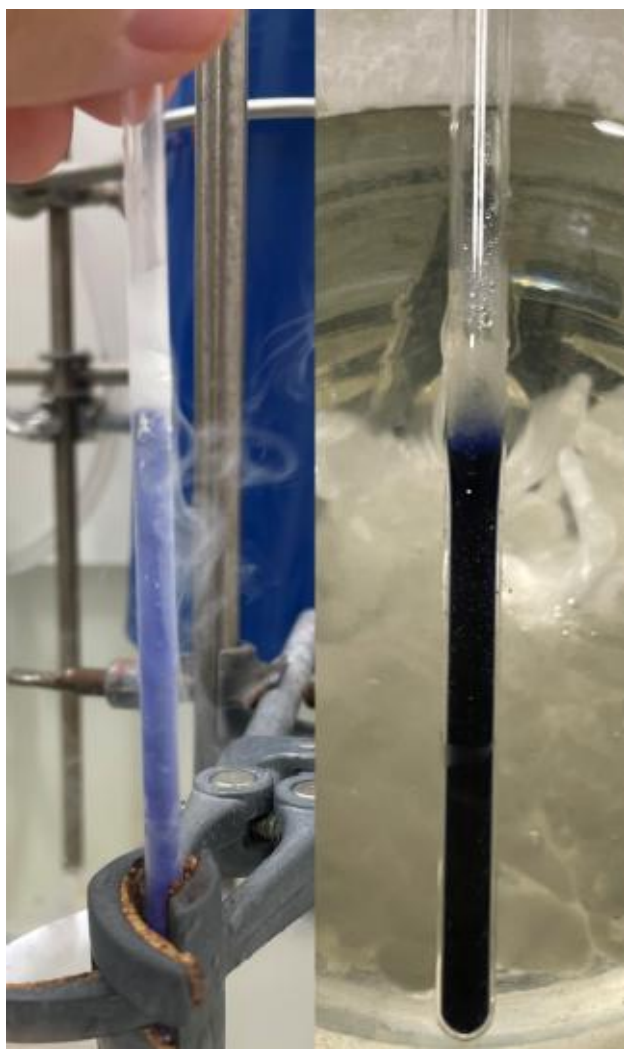

Figure S 1: NMR sample of **1**, at −174 °C as blue solid (left), and at −80 °C as dark blue liquid (right).

.

### 1.3 Synthesis of hexafluorothioacetone (1)

**Experimental setup**: A J-Young Schlenk filled with **2** was connected to a 42 cm long fused silica glass tube which was packed with approx. 7 cm of silica glass wool (***important: the wool must not be packed too tightly to allow unrestricted gas flow).*** This setup was placed horizontally in a tube furnace. The other end of the fused silica tube was connected to a cold trap consisting of a gas inlet pipe and a Schlenk tube. In this setup the actual Schlenk valve must be connected to the oven, and the gas inlet pipe valve must be connected to the Schlenk line. Otherwise, there is risk of blockage of the cold trap.

**Experimental procedure:** First the apparatus—except the storage container of **2**—was evacuated; then the oven was set to 600 °C. After the target temperature was reached, the cool trap was cooled with liquid nitrogen and the valve of the storage container of **2** was opened slowly. By evaporating **2**, it cooled down by itself that it became solid (m.p. 23.6 °C)[4] and thus created a slow and steady flow by itself. Seconds after opening the valve, the cold tap turned intensely blue, indicating the successful formation of **1**. After complete vaporization of **2,** the cold trap was disconnected from the silica tube, flushed with argon and cooled to −100 °C ***(important: guarantee equalization of pressure)***. Then the deep blue liquid **1** was purified from volatile impurities (see reference[5]) *in vacuo* at 100 K.

**1** should be stored at temperatures at −196 °C (liquid nitrogen) in the absence of light.

**$^{13}$C-NMR** (75 MHz, neat, 193 K) δ / ppm = 205.4 (*C*S, quint (theo. sept), $^{2}J_{CF}$ = 37.71 Hz), 116.7 (2×*C*F$_3$, *q*, $^{1}J_{CF}$ = 278.67 Hz).

**$^{19}$F-NMR** (282.5 MHz, $C_6D_6$, r.t.) δ / ppm = −69 (2×C*F*$_3$).

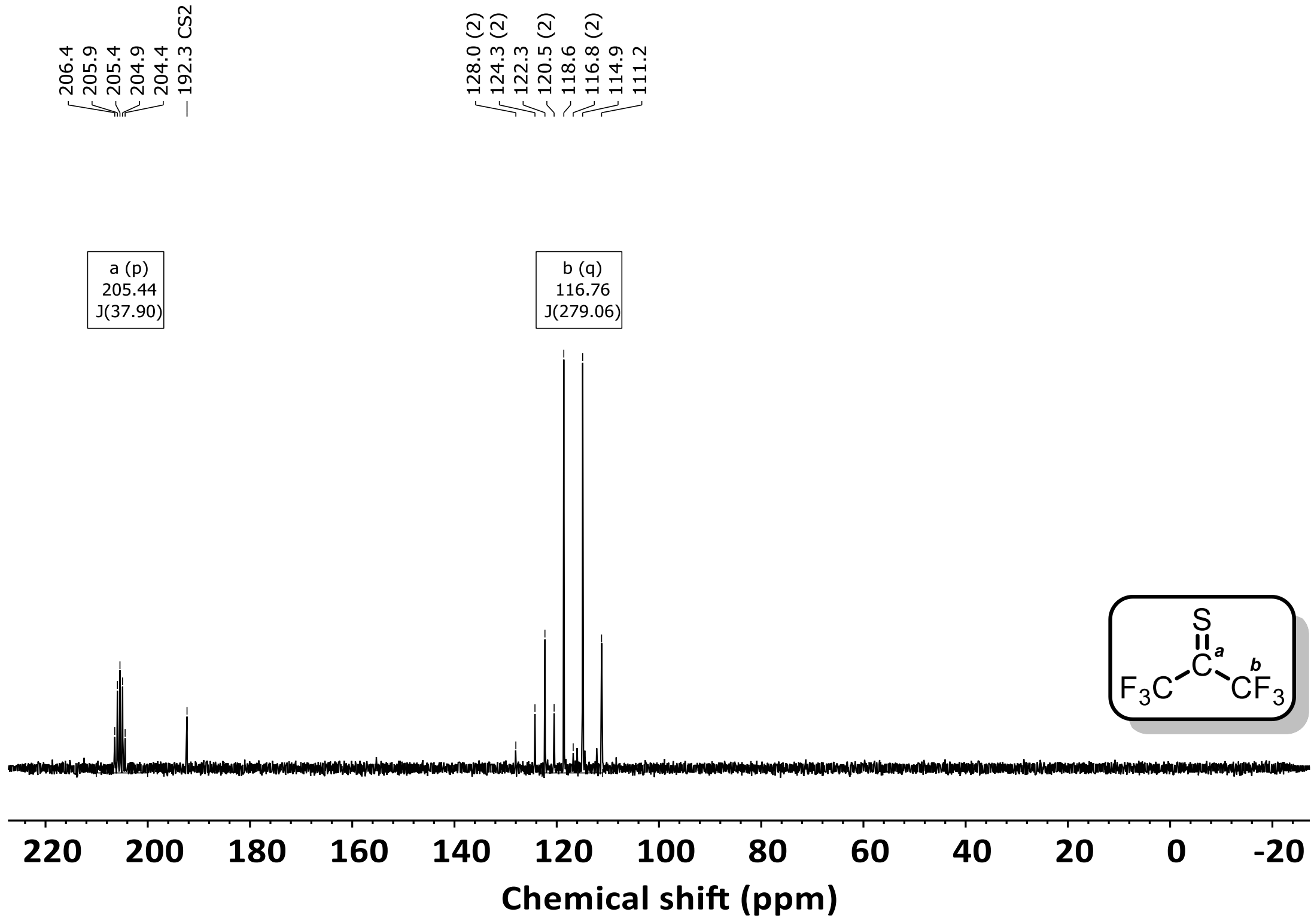


Figure S 2: $^{13}$C-NMR ($^{19}$F coupled) spectrum of **1** with traces of **2** and $CS_2$ at 193 K.

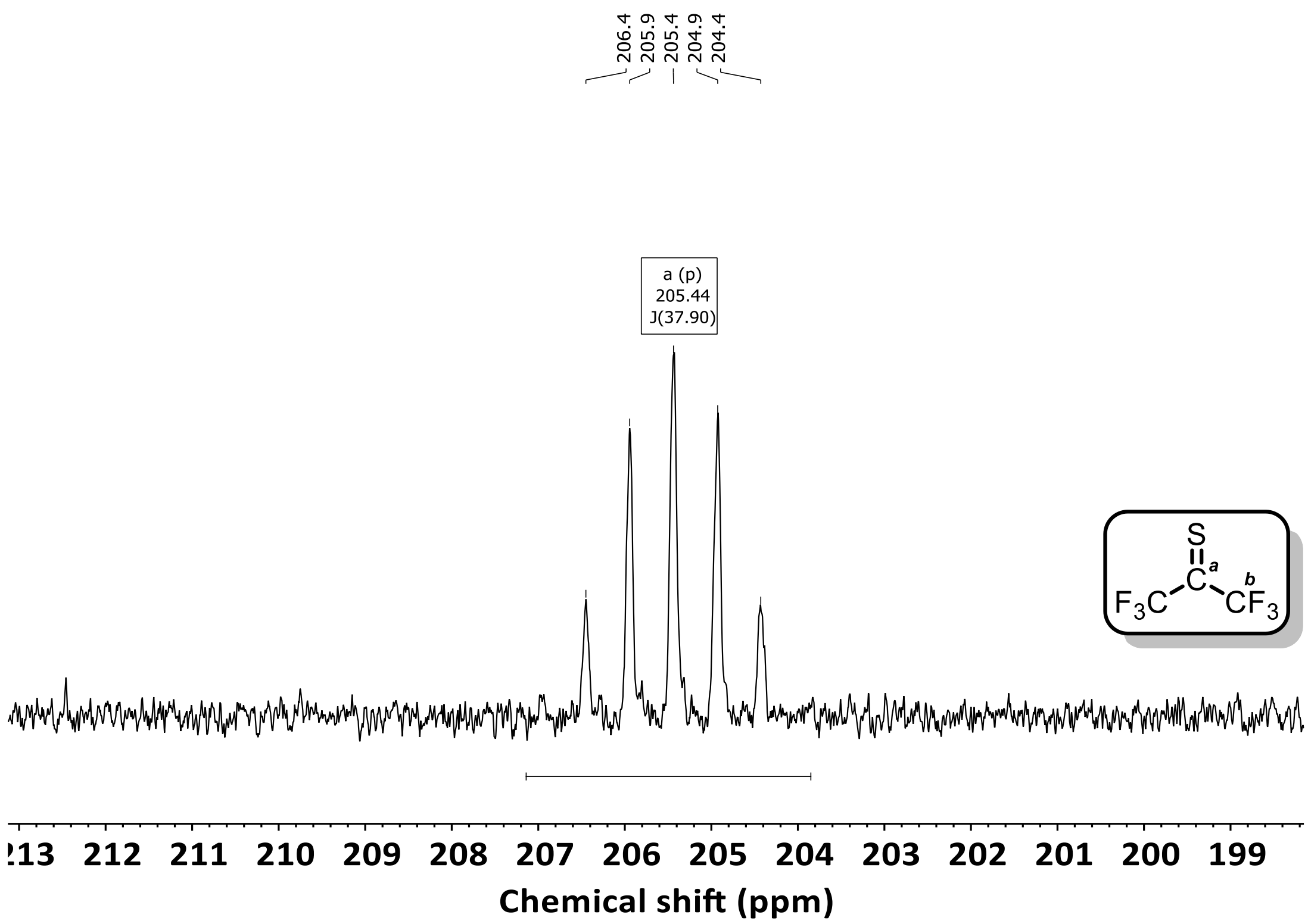


Figure S 3: Enlargement of the signal at 205.44 ppm of the $^{13}$C-NMR of **1** at 193 K.

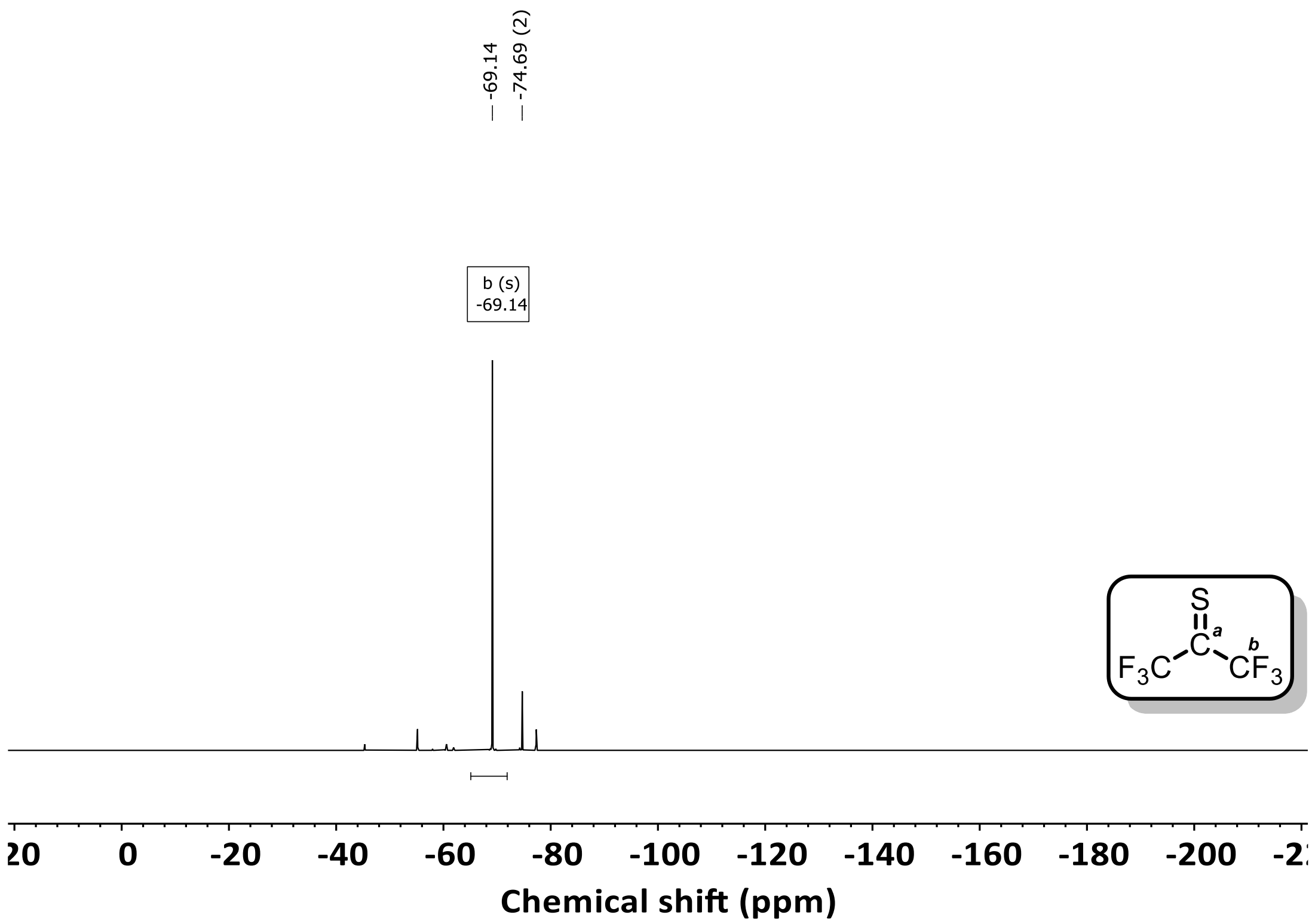


Figure S 4: $^{19}$F-NMR spectrum of **1** at 193 K. Other signals than the main signal could not be assigned.

## 1.4 Synthesis of cycloocta-1,5-diene(hexafluorothioacetone)nickel (3)

**3** was synthesized after a modified literature[6] protocol:

[Ni(COD)$_2$] (219.00 mg, 796.19 µmol, 1.00 eq.) was suspended in diethyl ether (5 mL) and cooled to −60 °C. Into the yellow suspension **2** (0.5 mL, 2.75 mmol, 3.45 eq.) was added dropwise *(**note**: sticking the syringe too deep into the reaction container leads to blockage because of solidification of **2**)*. Then the cooling bath was removed. After reaching room temperature, the reaction mixture was stirred for approx. 15 min. yielding a dark brown-yellow suspension which was subsequently filtered through a filter cannula (pore size: 0.2 µm, PVDF filter media). The solvent of the resulting dark brown-yellow solution was removed *in vacuo*. **3** (188.00 mg, 538.74 µmol, 68%) was isolated as brown-yellow crystalline solid. Crystals suitable for single crystal X-ray diffraction were obtained from the bulk sample.

**$^1$H-NMR** (300 MHz, $C_6D_6$, r.t.) δ / ppm = 5.24 (s, 2×$C_{sp2}$–H), 4.99 (s, 2×$C_{sp2}$–H), 1.78 (s, 2×$C_{sp3}$–$H_2$), 1.43 (s, 2×$C_{sp3}$–$H_2$).

**$^{13}$C{$^1$H, $^{19}$F}-NMR** (150 MHz, $C_6D_6$, r.t.) δ / ppm = 128.3 (s, 1×*C*$F_3$), 125.6 (s, 1×*C*$F_3$), 106.8 (2×$C_{sp2}$), 102.6 (2×$C_{sp2}$), 59.8 (*C*S), 30.0 (2×$C_{sp3}$), 28.6 (2×$C_{sp3}$).

**$^{19}$F-NMR** (282.5 MHz, $C_6D_6$, r.t.) δ / ppm = −56.6 (2×C*$F_3$*).

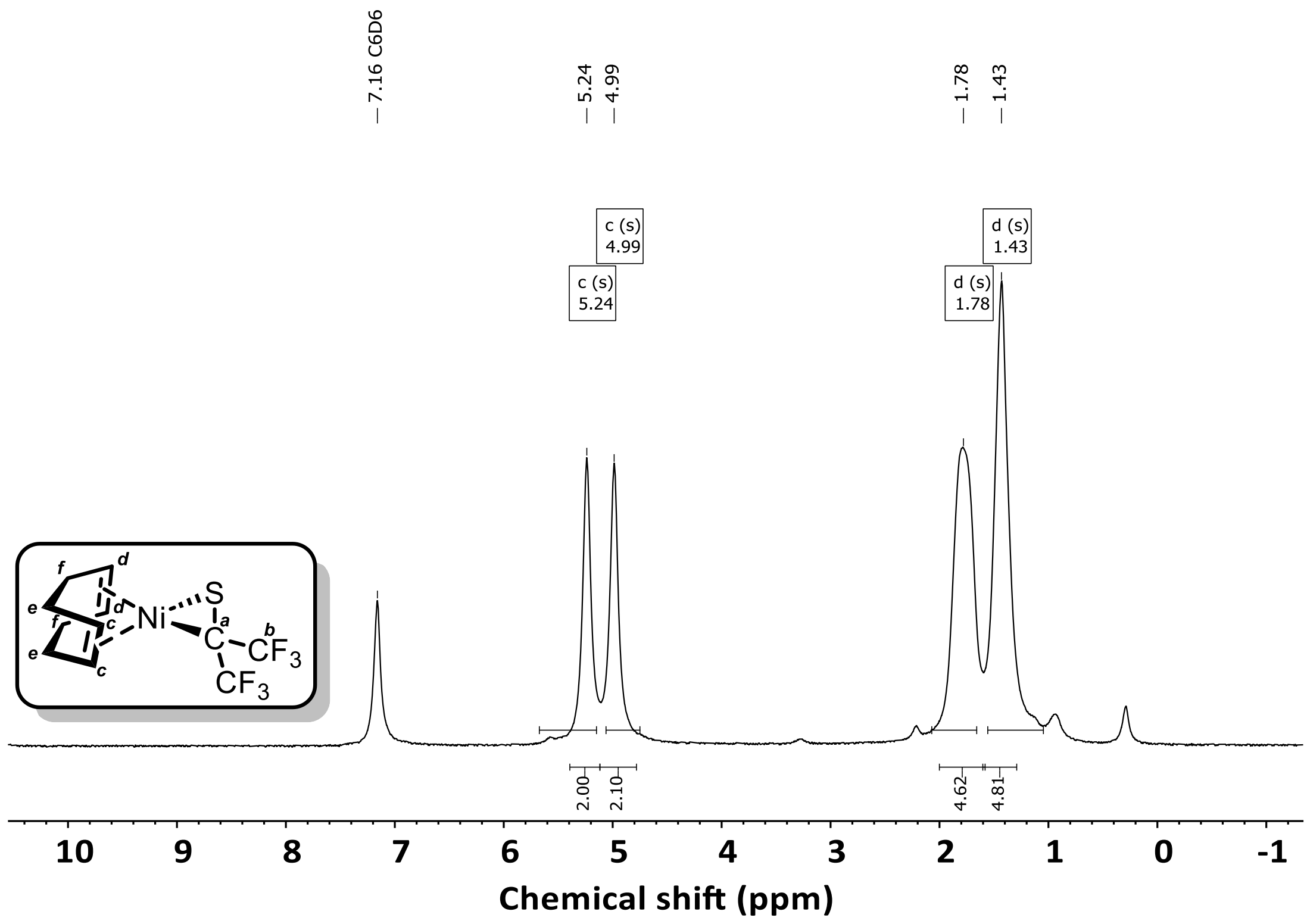


Figure S 5: $^{1}H$-NMR spectrum of **3** in $C_6D_6$ at rt.

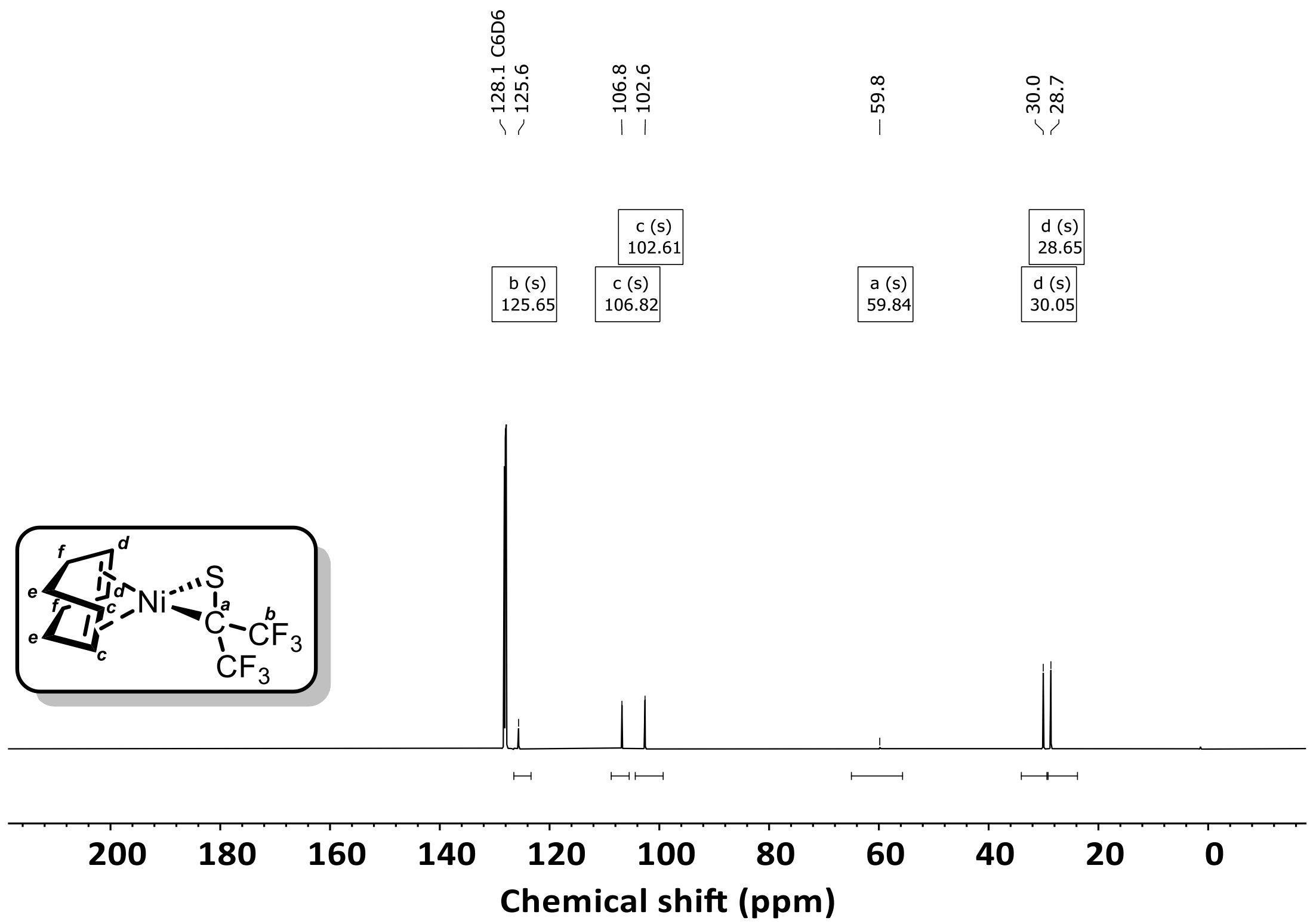


Figure S 6: $^{13}C\{^{1}H, ^{19}F\}$-NMR spectrum of **3** in $C_6D_6$ at rt.

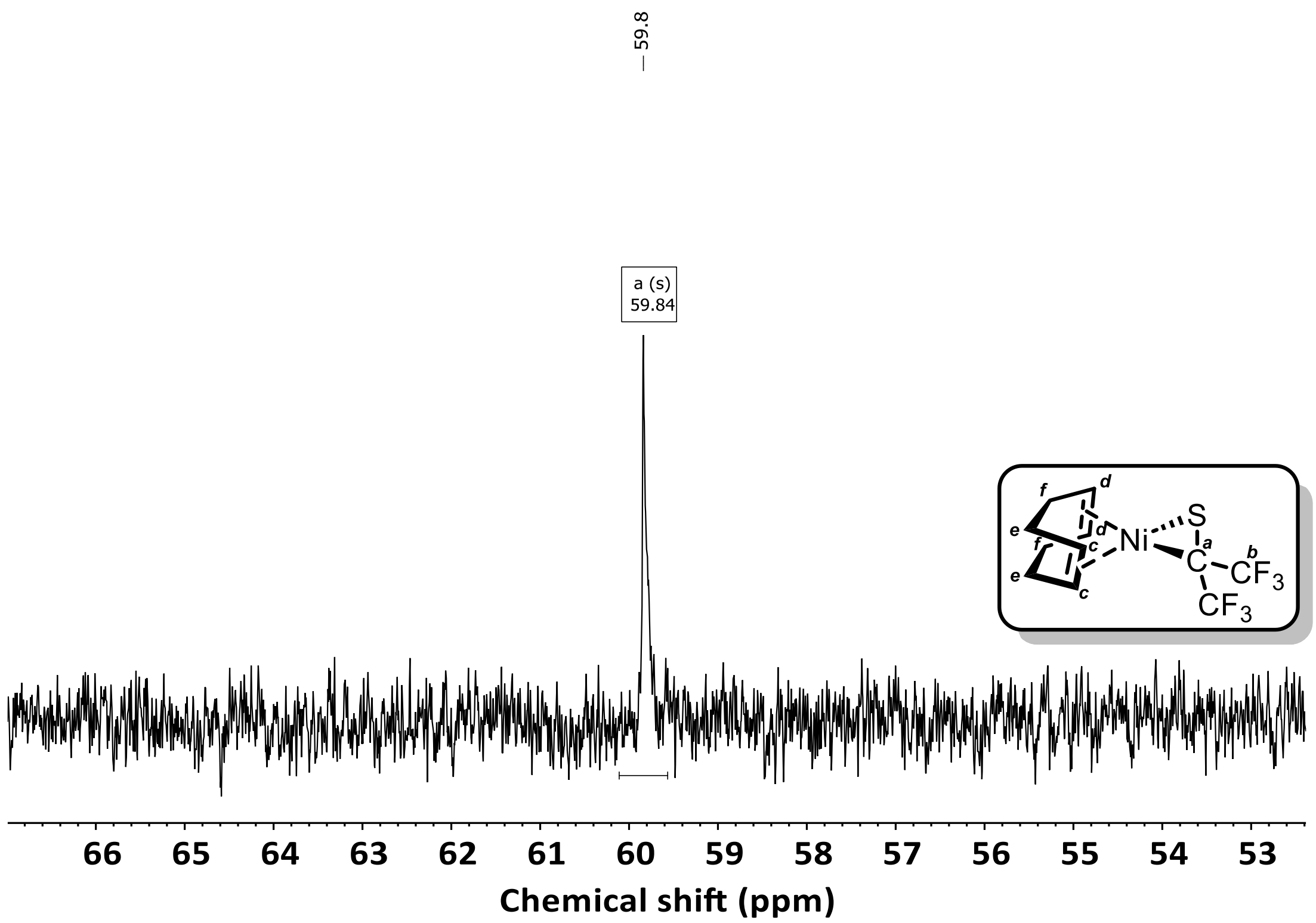


Figure S 7: Enlargement of signal **a** at 59.84 ppm of the $^{13}C\{^{1}H, ^{19}F\}$-NMR of **3**.

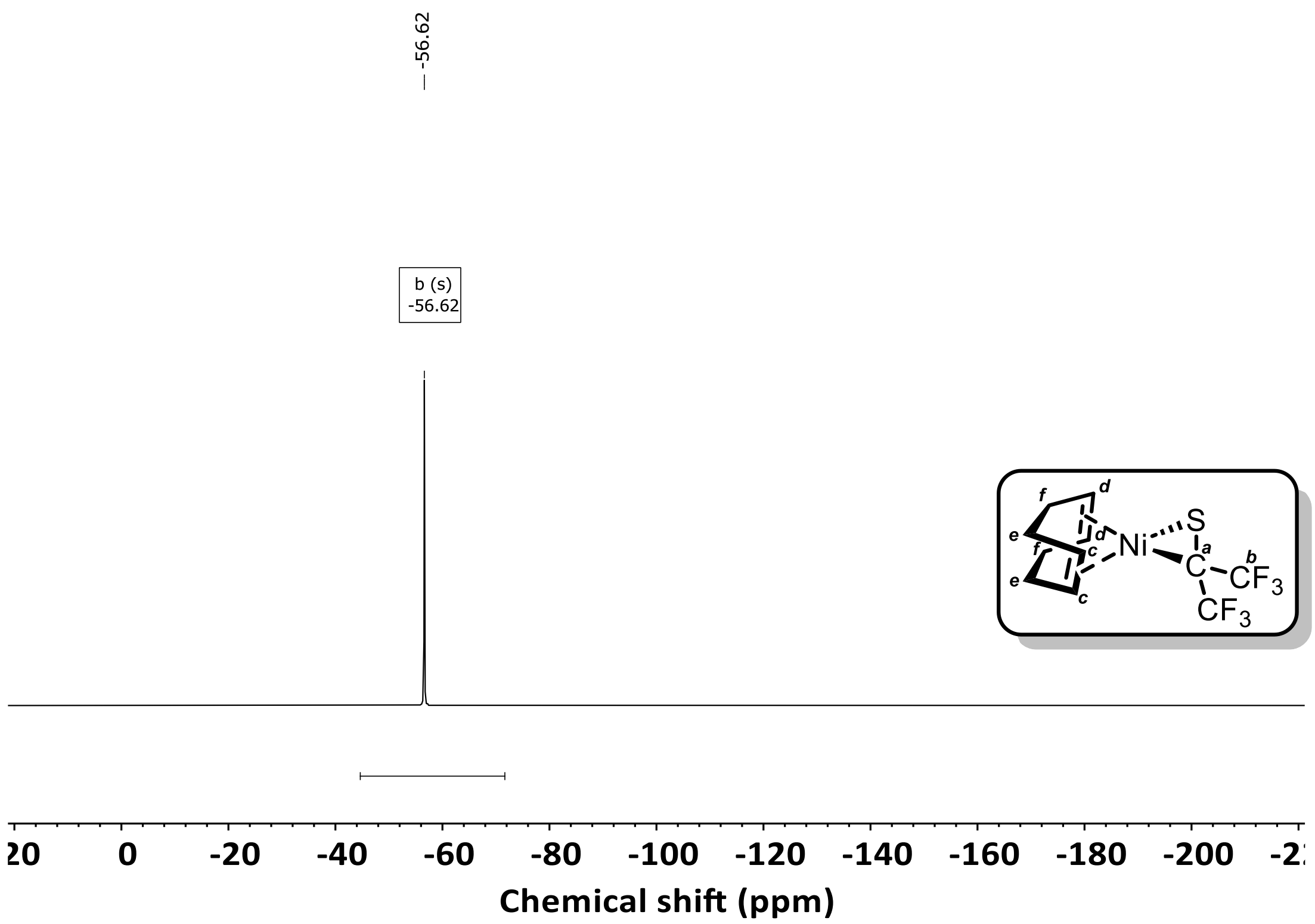


Figure S 8: $^{19}F$-NMR spectrum of **3** in $C_6D_6$ at rt.

### 1.5 Synthesis of bispyridine(hexafluorothioacetone)nickel (4)

**3** (70 mg, 200.60 µmol, 1.00 eq.) was suspended in *n*-hexane (7 mL) and the reaction mixture was cooled to −60 °C. Then, pyridine (0.1 mL, 1.24 mmol, 8.31 eq.) was added dropwise. Then, the cooling bath was removed and after reaching room temperature the reaction mixture was stirred for 60 min. Afterwards the liquid phase of the red-brown suspension (initial brown-yellow) was decanted, and the residue was dried in vacuo at 40 °C. The product was obtained as red-brown solid (43.00 mg, 107.77 µmol, 54%). Crystals suitable for single crystal X-ray diffraction were obtained by removing solvent of a solution of **3** in dichloromethane.

**$^{1}$H NMR** (600 MHz, $C_6D_6$, r.t.) δ / ppm = 8.62 (s, 2×$C_{sp2}$–H-ortho), 8.00 (s, 2×$C_{sp2}$–H-ortho), 6.69 (s, 2×$C_{sp2}$–H-para), 6.41 (s, 2×$C_{sp2}$–H-meta), 6.24 (s, 2×$C_{sp2}$–H-meta).

**$^{13}$C{$^{1}$H} NMR** (150 MHz, $C_6D_6$, r.t.) δ / ppm = 151.5 (s, 2×$C_{sp2}$-ortho), 136.6 (s, 1×$C_{sp2}$-para), 136.5 (s, 1×$C_{sp2}$-para), 127.8 (q, $^{1}J_{13C19F}$ = 275.25 Hz, 2×$CF_3$), 124.5 (s, 1×$C_{sp2}$-meta), 124.4 (s, 1×$C_{sp2}$-meta), 46.3 (t, $^{2}J_{13C19F}$ = 33.10 Hz, 1×CS).

**$^{19}$F NMR** (564 MHz, $C_6D_6$, r.t.) δ / ppm = −55.5 (s, 2×$CF_3$)

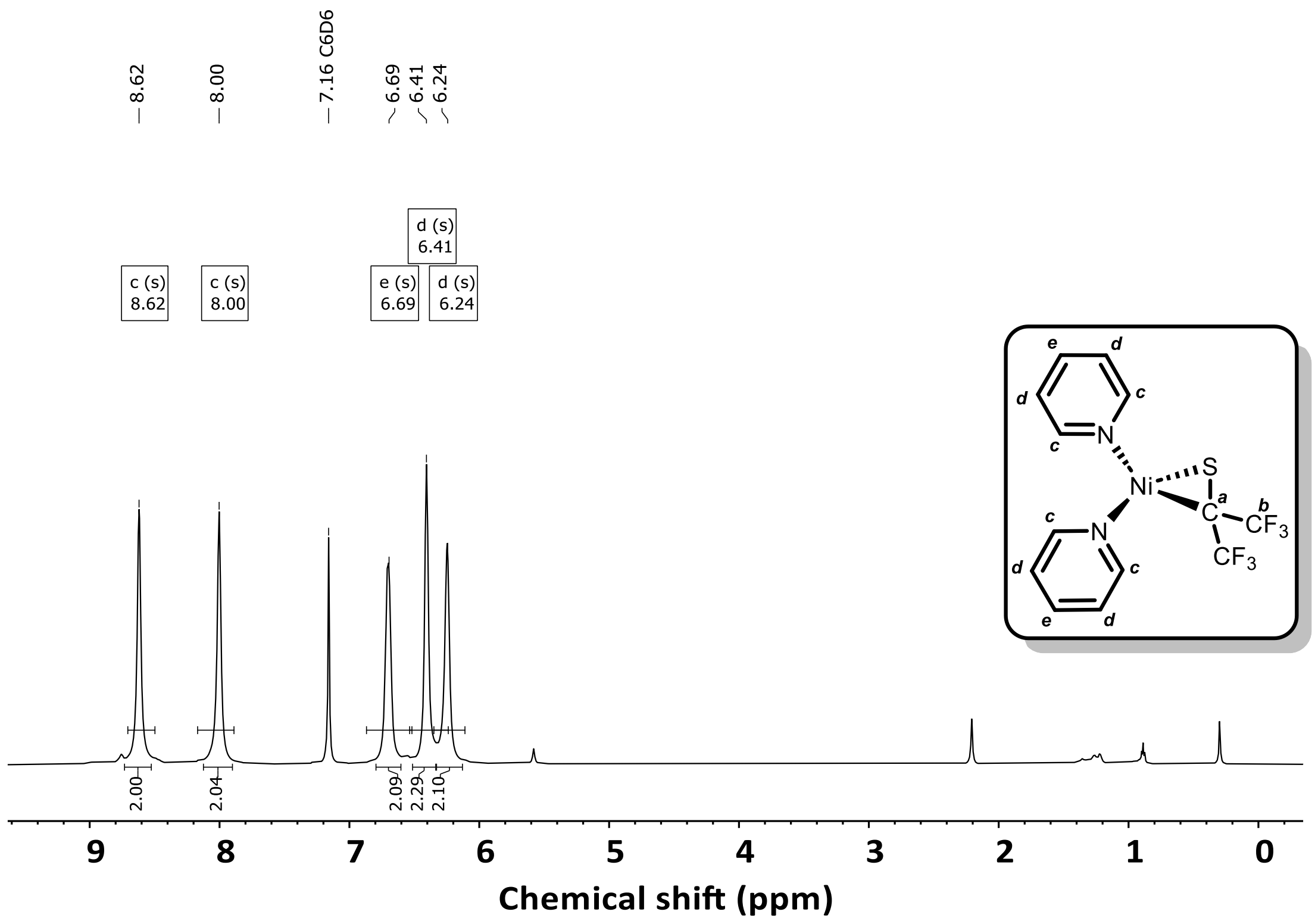


Figure S 9: $^{1}H$-NMR spectrum of **4** in $C_6D_6$ at rt.

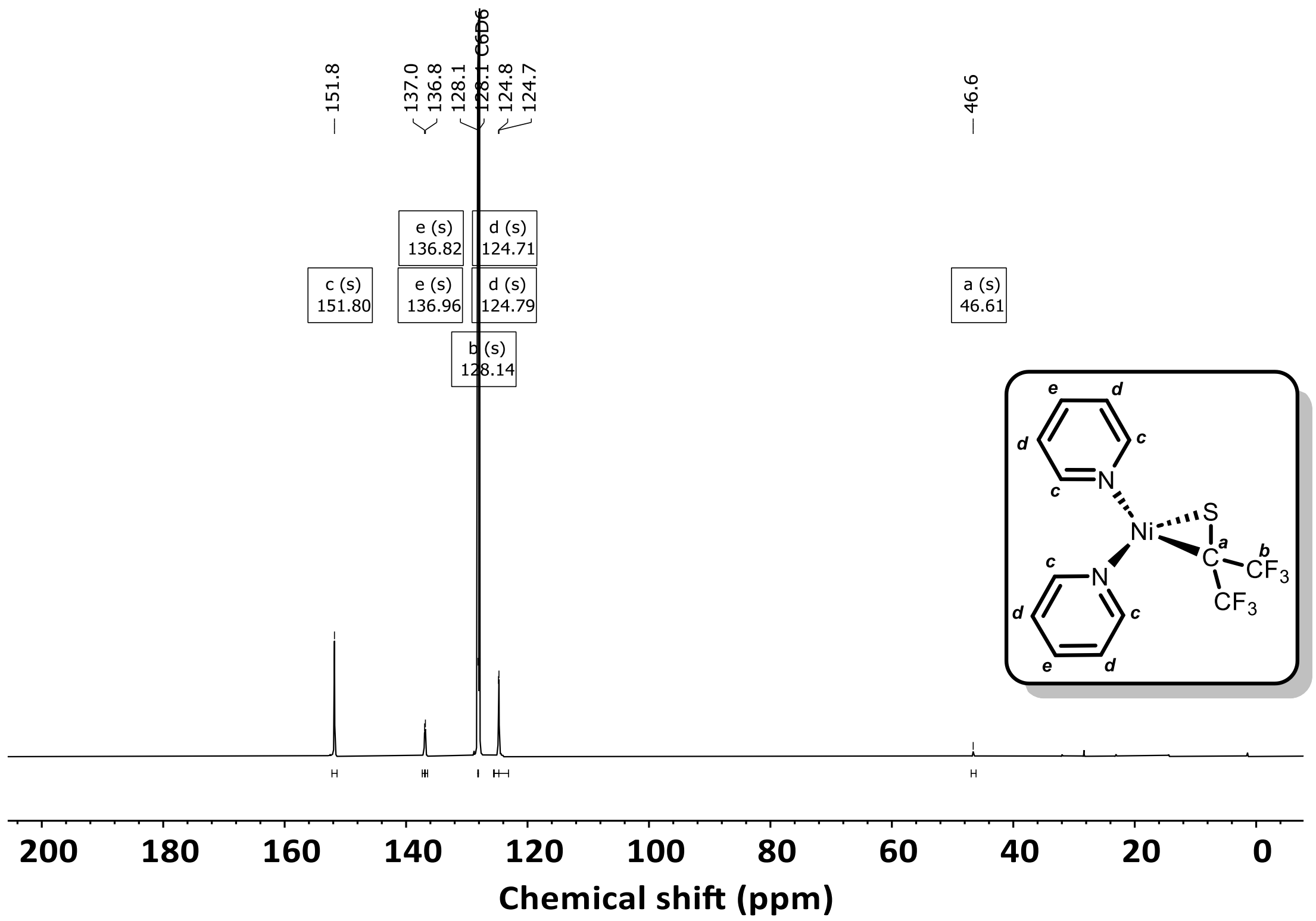


Figure S 10: $^{13}C\{^{1}H, ^{19}F\}$-NMR spectrum of **4** in $C_6D_6$ at rt.

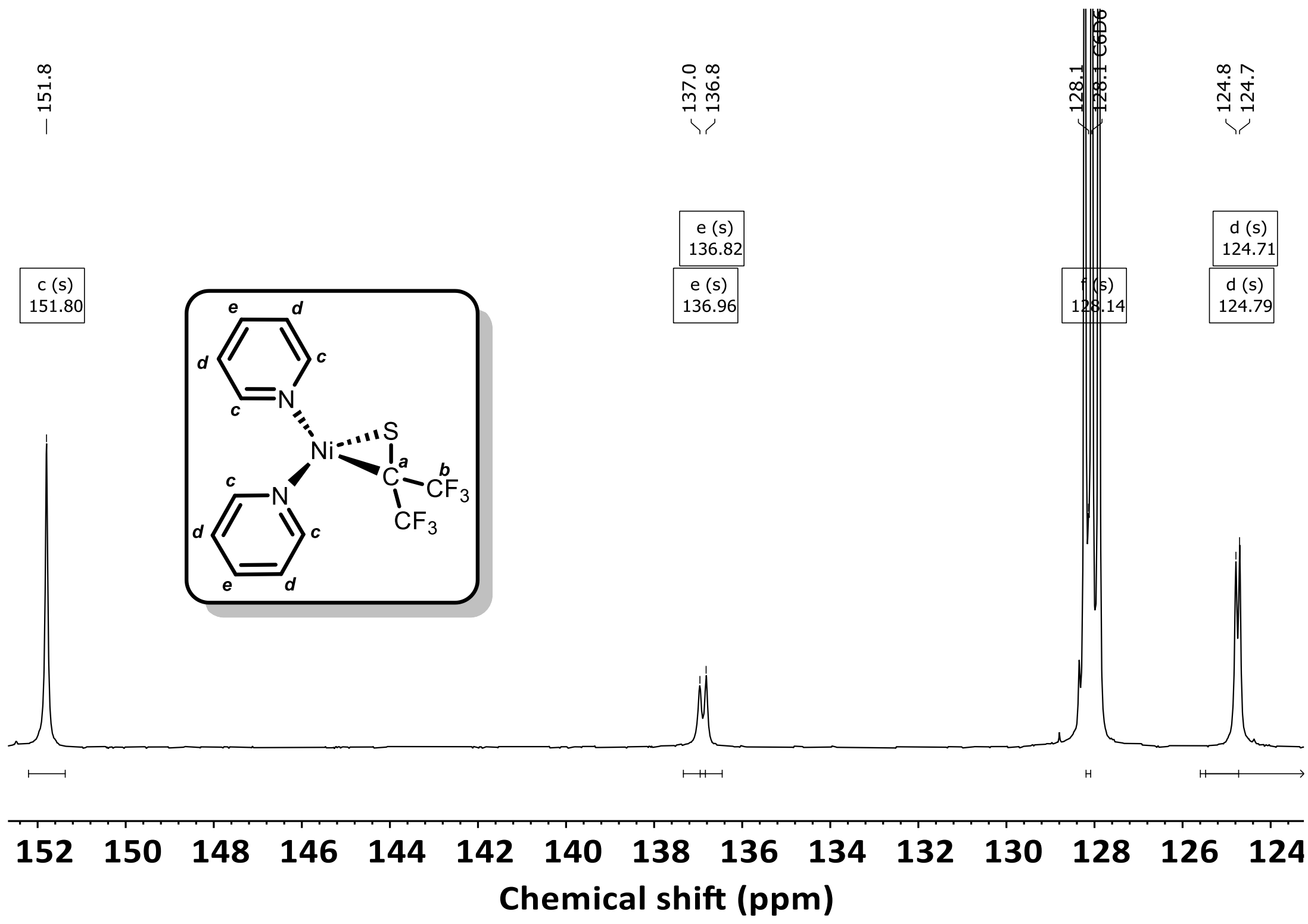


Figure S 11: Enlargement of signals at 124–152 ppm of the $^{13}C\{^1H, ^{19}F\}$-NMR spectrum of **4**.

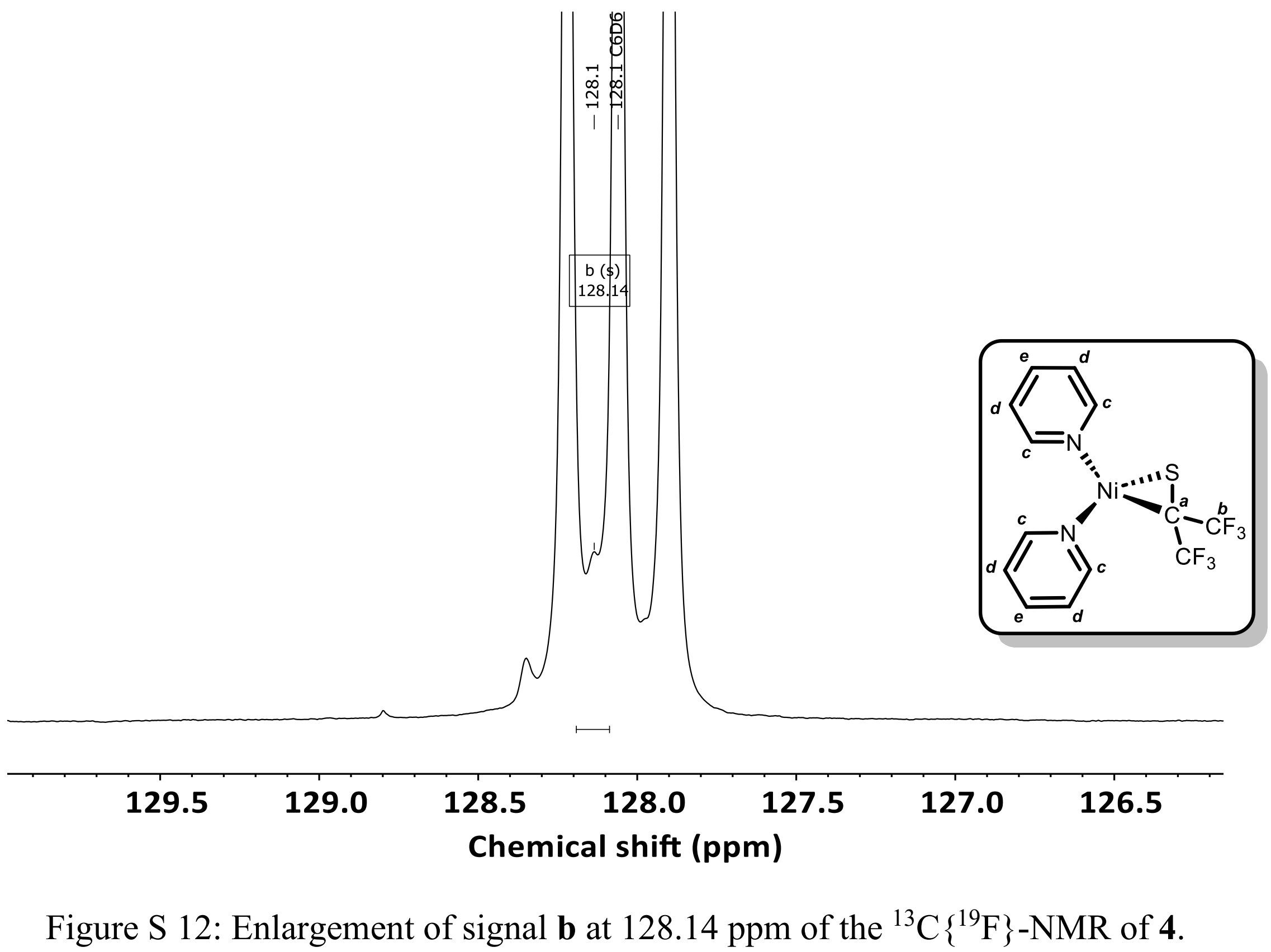


Figure S 12: Enlargement of signal **b** at 128.14 ppm of the $^{13}C\{^{19}F\}$-NMR of **4**.

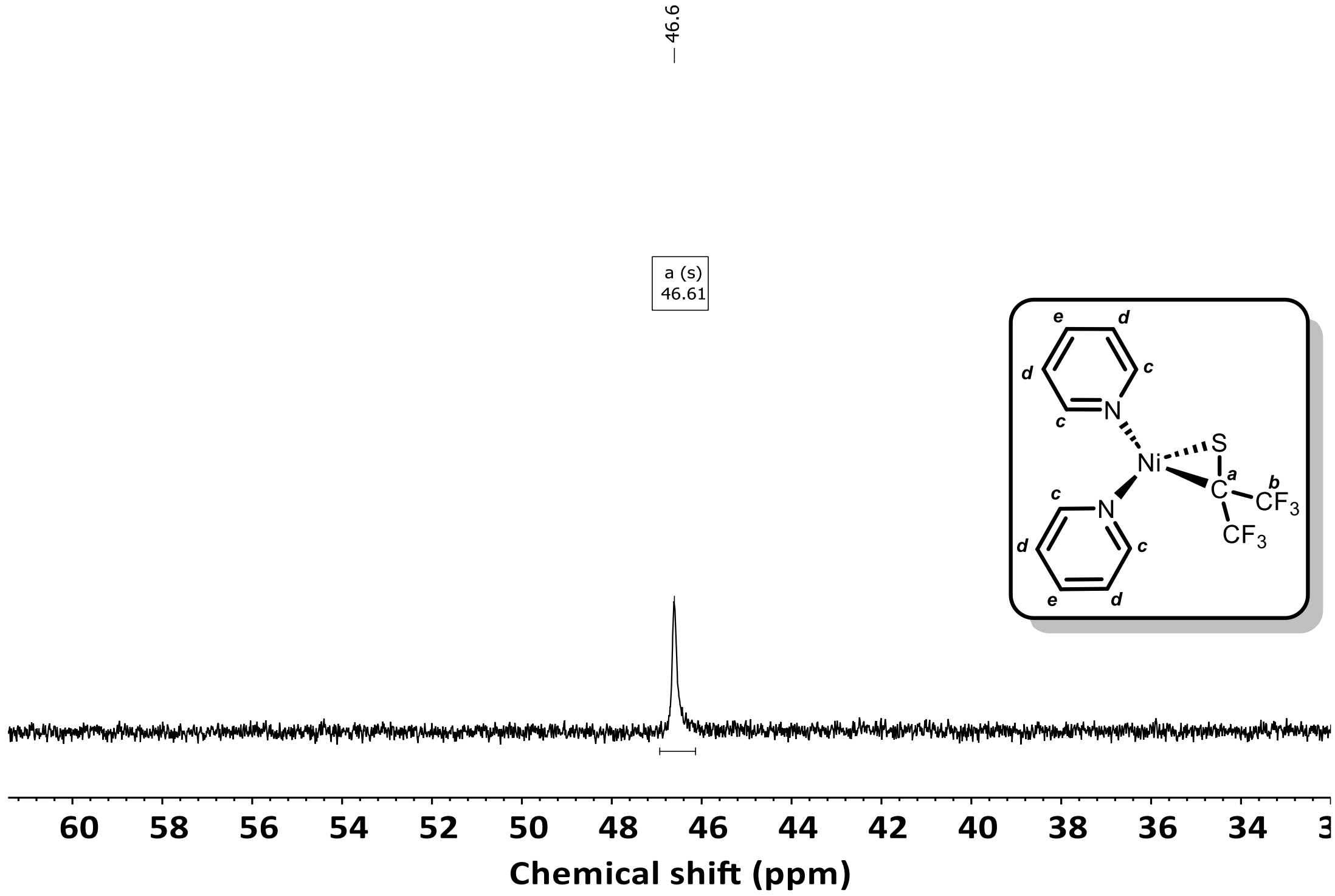


Figure S 13: Enlargement of signal **a** at 46.6 ppm of the $^{13}C\{^{19}F\}$-NMR of **4**.

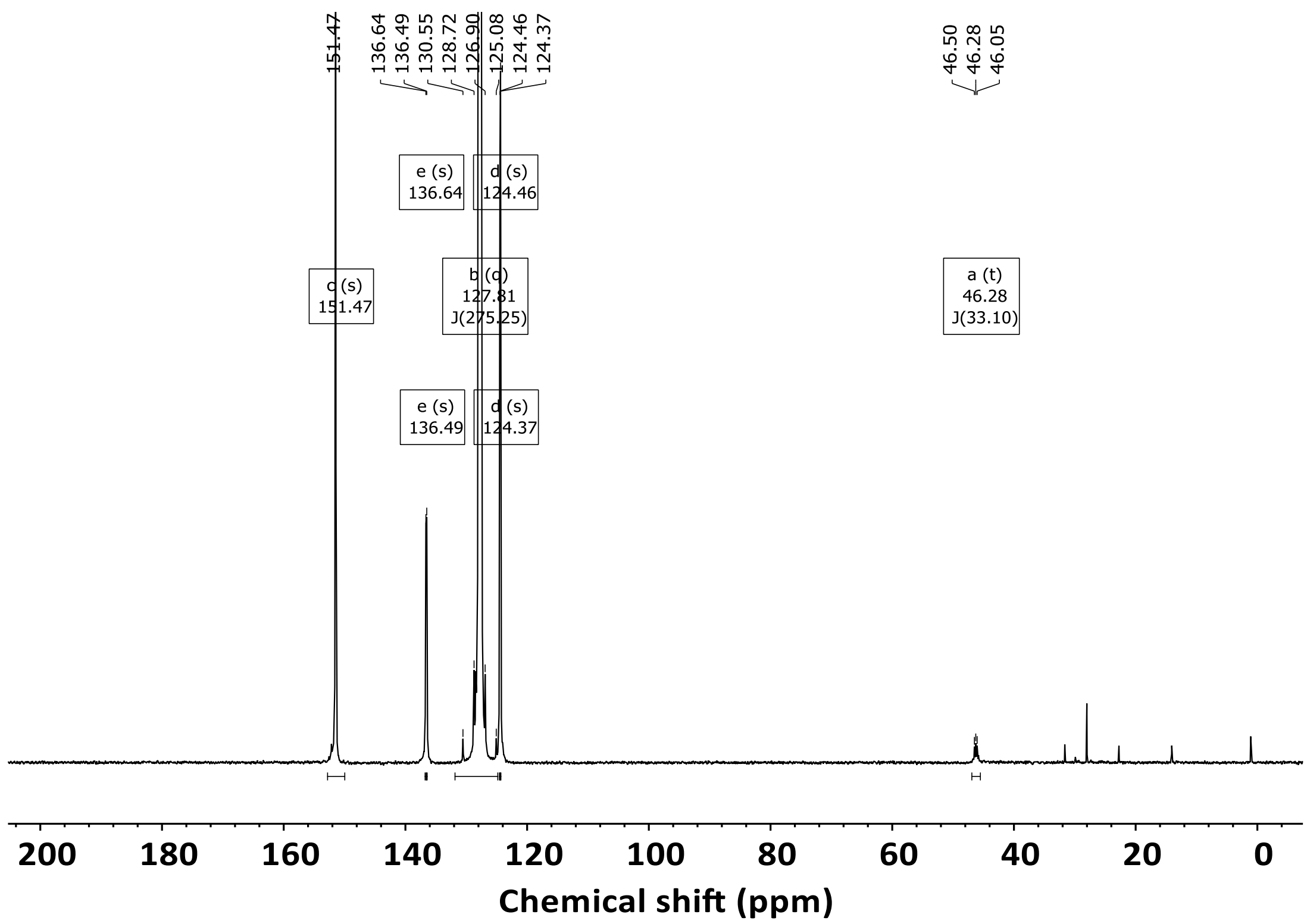


Figure S 14: $^{13}C\{^{1}H\}$-NMR spectrum of **4** in $C_6D_6$ at rt.

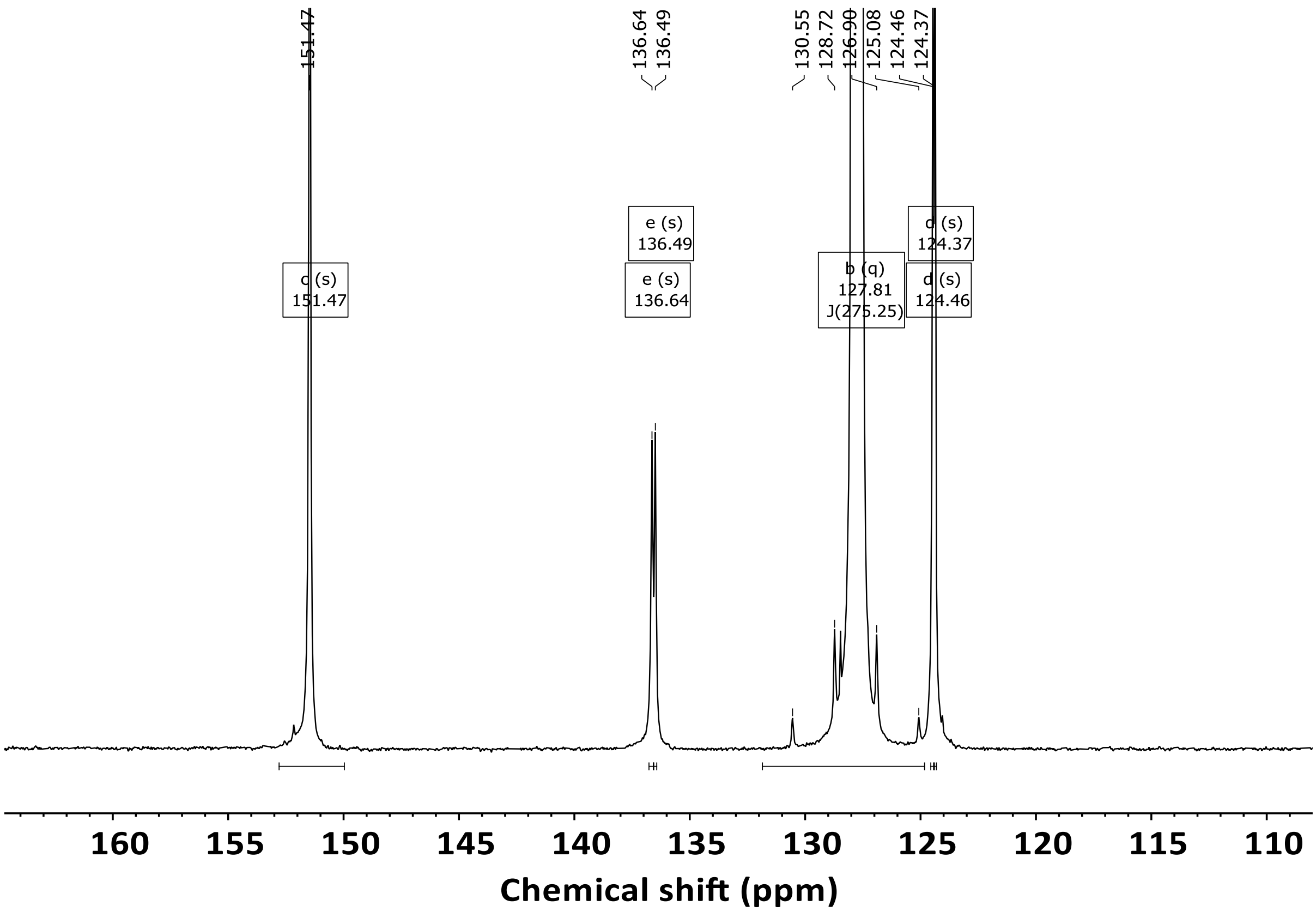


Figure S 15: Enlargement of signals at 105–170 ppm of the $^{13}C\{^{1}H\}$-NMR spectrum of **4**.

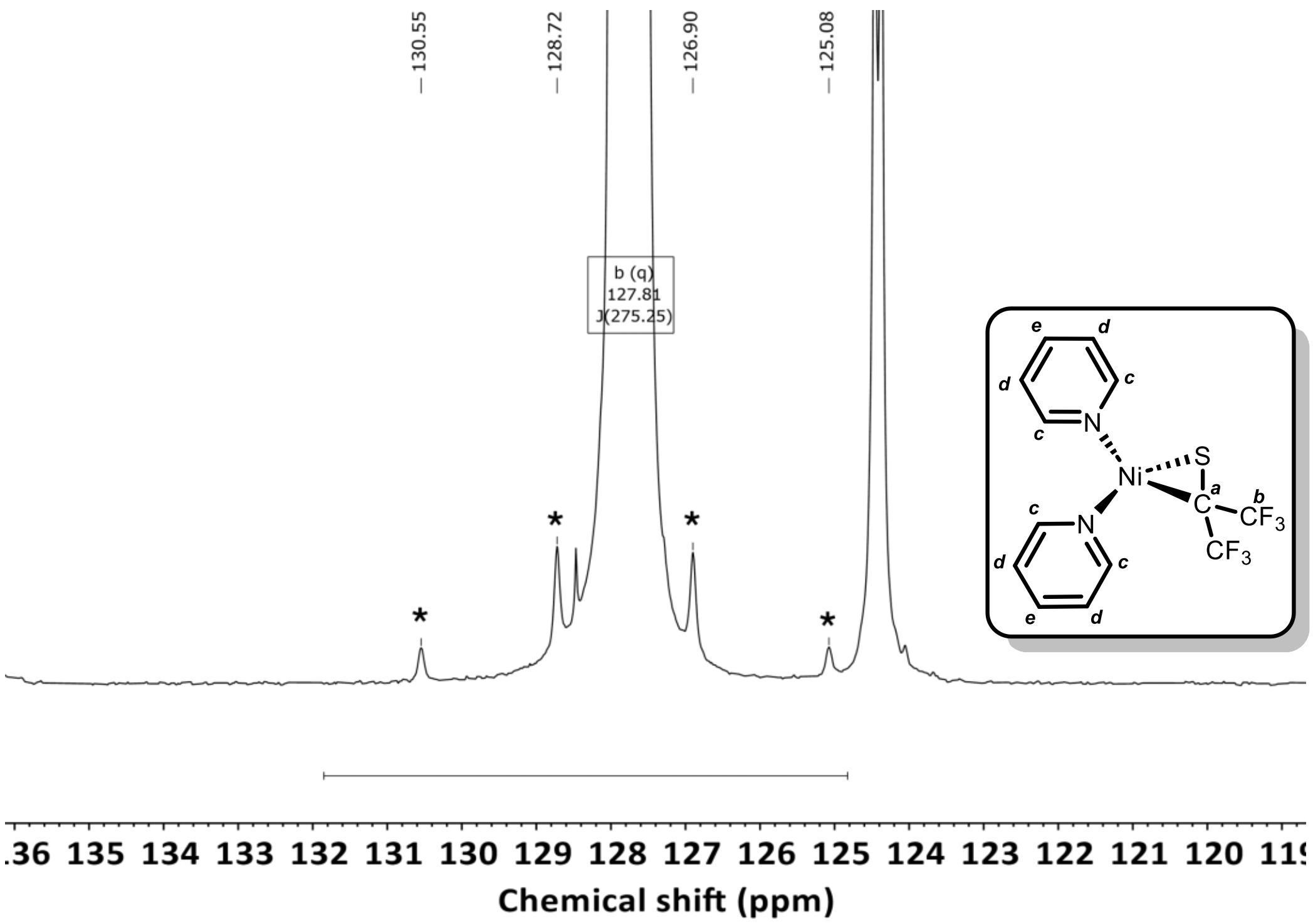


Figure S 16: Enlargement of signal **b** at 127.8 ppm of the $^{13}C\{^{1}H\}$-NMR of **4**.

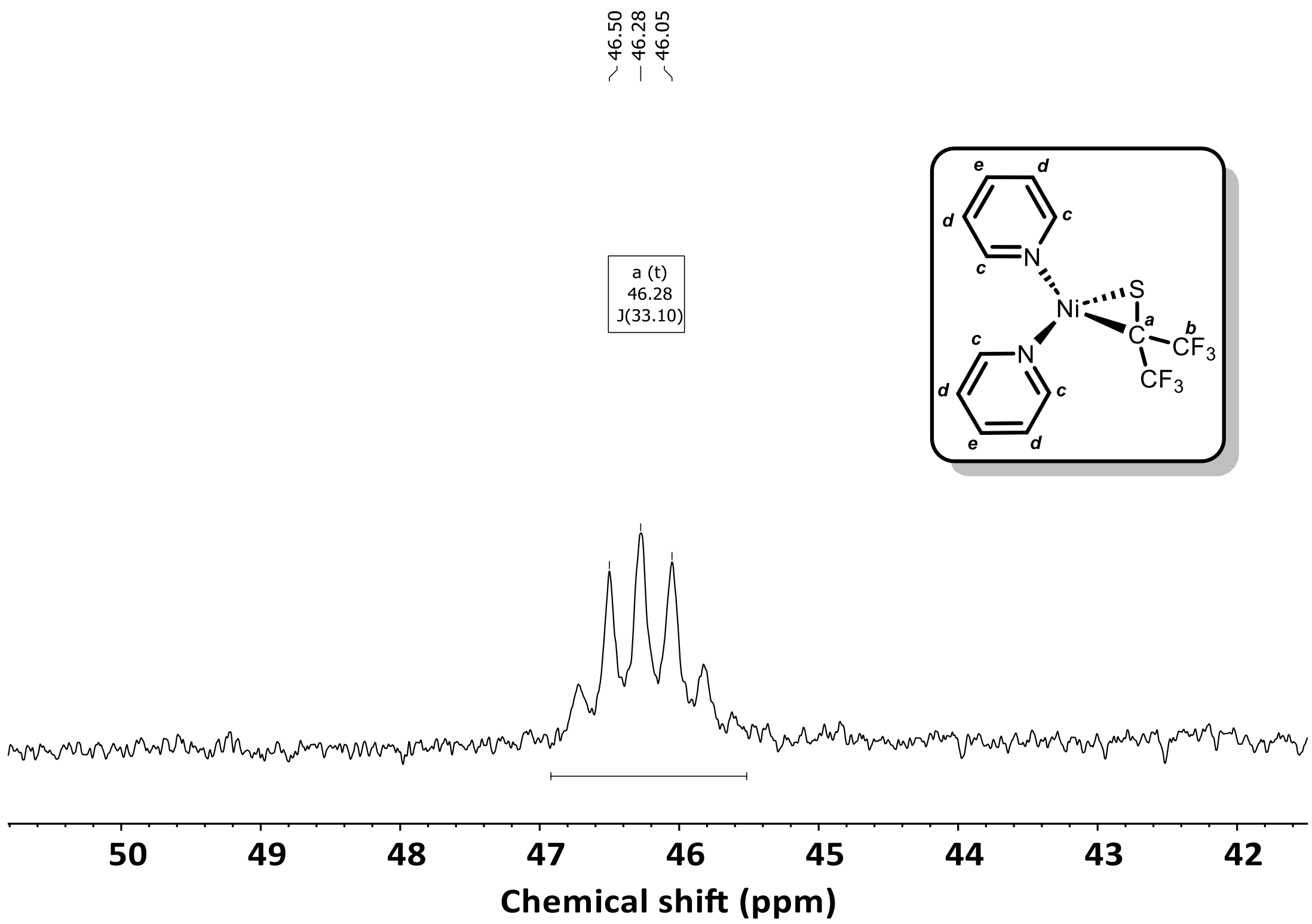


Figure S 17: Enlargement of signal **a** at 46.28 ppm of the $^{13}C\{^{1}H\}$-NMR of **4**.

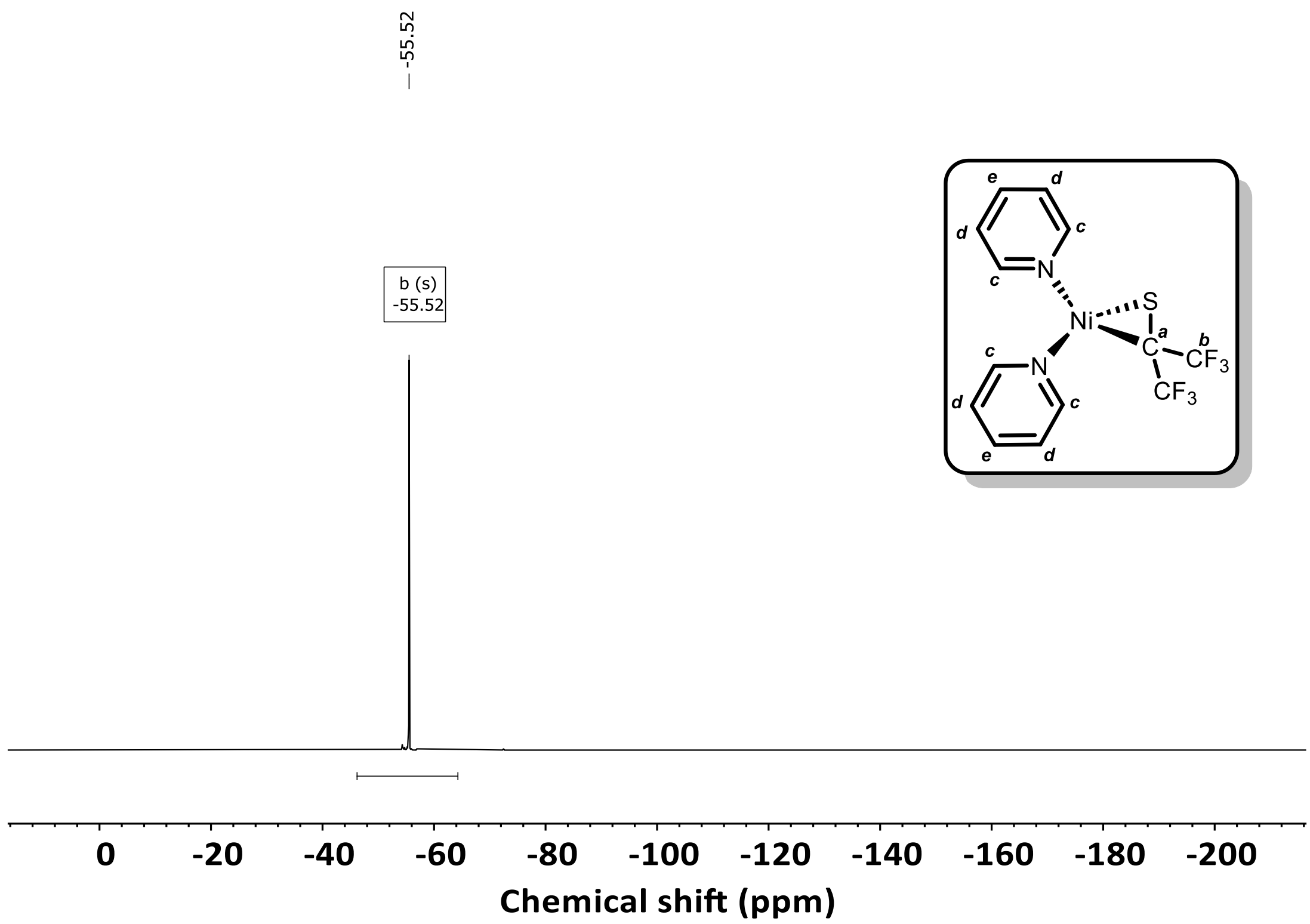


Figure S 18: $^{19}$F-NMR spectrum of **4** in $C_6D_6$ at rt.

### 1.5 Synthesis of bistetramethylendiamine(hexafluorothioacetone)nickel (5)

**3** (62 mg, 177.67 µmol, 1.00 eq.) was dissolved in diethyl ether (5 mL) and the reaction mixture was cooled to 0 °C. Then tetramethyl ethylenediamine (0.1 mL, 662.60 µmol, 5.00 eq.) was added dropwise and the colour of the reaction mixture changed to a faint red from the initial brown yellow. The cooling bath was removed and after reaching room temperature the reaction mixture was stirred for 60 min. Afterwards the solvent of the faint red solution was removed *in vacuo* and the residue was washed with *n*-hexane (2 × 2mL). Finally, the product was extracted with dichloromethane (4 mL) and subsequently dried *in vacuo* to give **5** (21 mg, 58.83 µmol, 33%) as red powder. Crystals suitable for single crystal X-ray diffraction were obtained by removing the solvent of the crude reaction mixture.

**$^{1}$H-NMR** (600 MHz, $CDCl_3$, r.t.) δ / ppm = 2.76 (s, 2×$CH_3$), 2.71 (s, 2×$CH_3$), 2.53 (m, 1×$CH_2$), 2.4.0 (m, 1×$CH_2$).

**$^{13}$C{$^{1}$H} NMR** (150 MHz, $C_6D_6$, r.t.) δ / ppm = 127.1 (q, $^{1}J_{13C19F}$ = 274.82 Hz, 2×$CF_3$), 60.9 (s, 1×$CH_2$), 58.6 (s, 1×$CH_2$), 51.0 (s, 2×$CH_3$), 50.0 (s, 2×$CH_3$), 41.7 (p, $^{2}J_{13C19F}$ = 34.62 Hz, 1×CS).

**$^{19}$F-NMR** (564 MHz, $C_6D_6$, r.t.) δ / ppm = −55.95 (s, 2×$CF_3$).

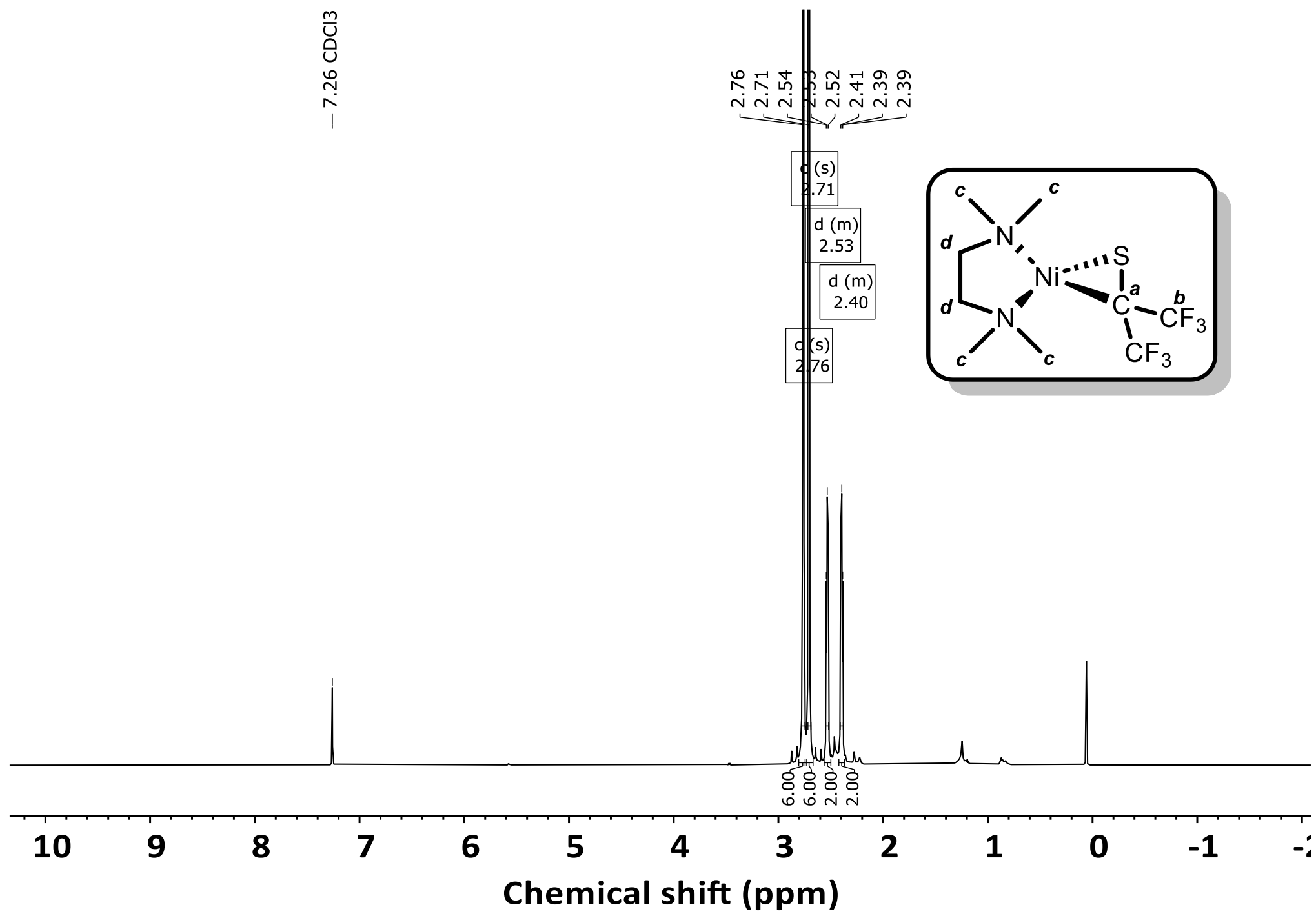


Figure S 19: $^{1}$H-NMR spectrum of **5** in $CDCl_3$ at rt.

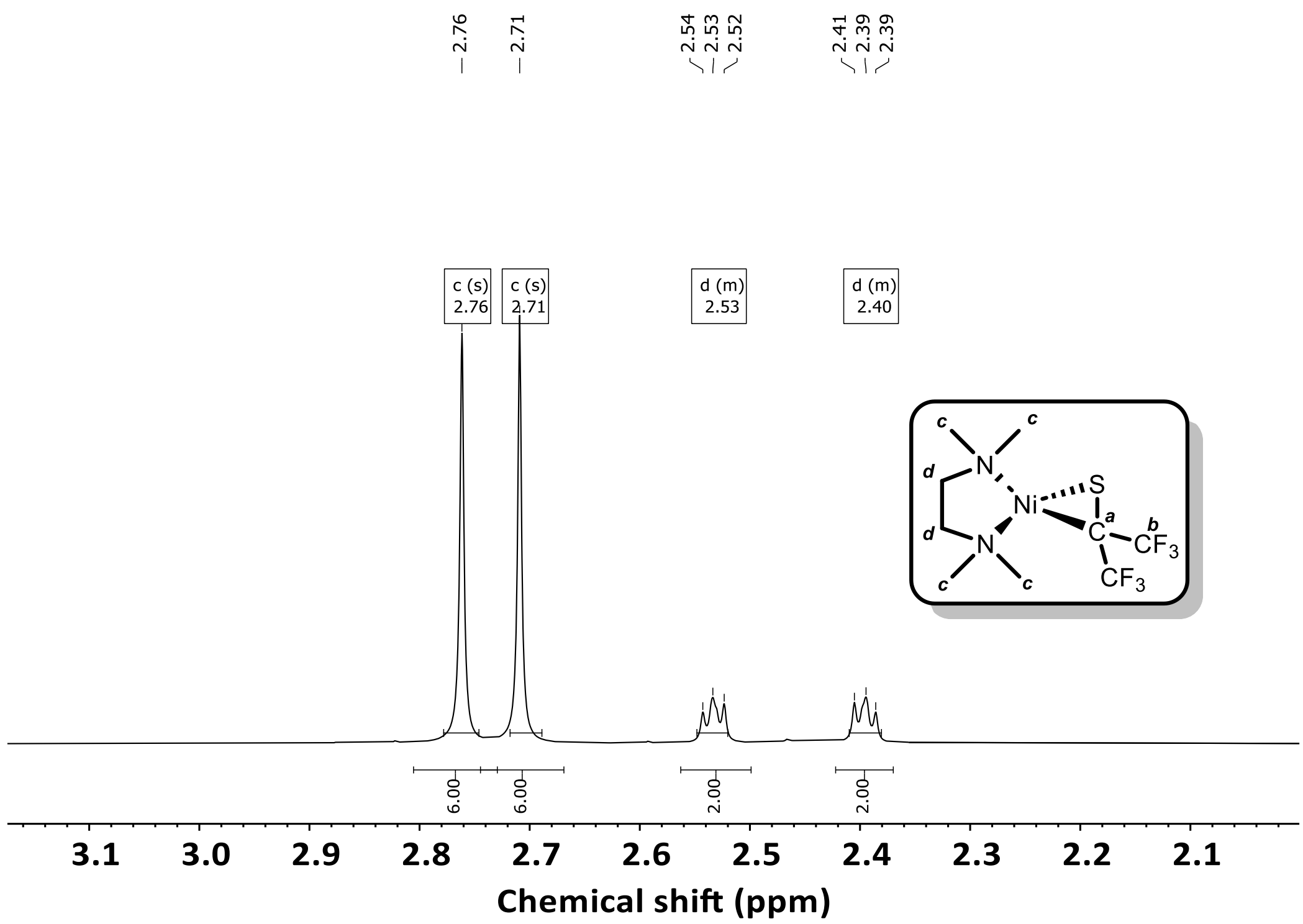


Figure S 20: Enlargement of signals at 2.1–3.1 ppm of the $^{1}$H-NMR spectrum of **5**.

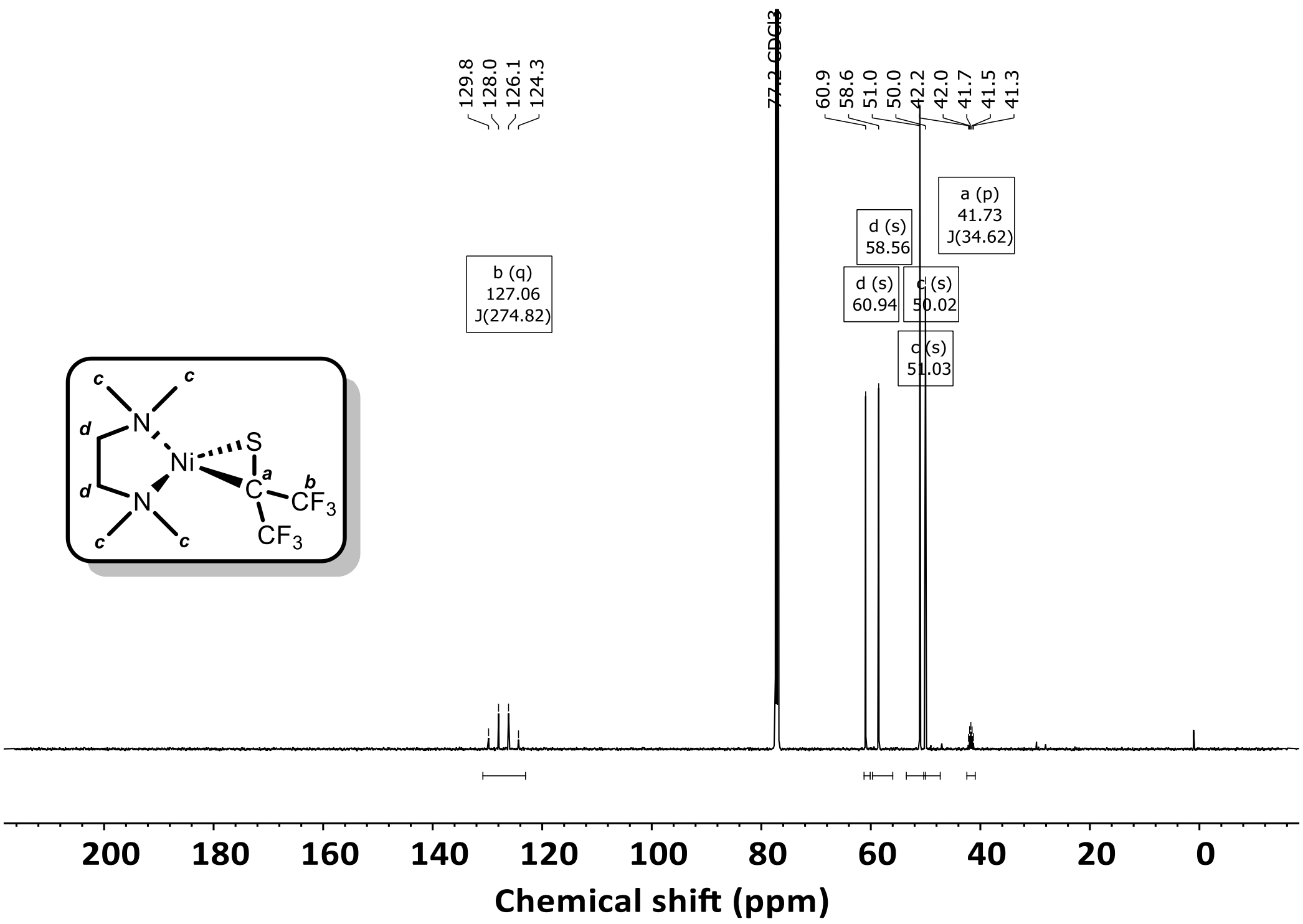


Figure S 21: $^{13}C\{^1H\}$-NMR spectrum of **5** in $CDCl_3$ at rt.

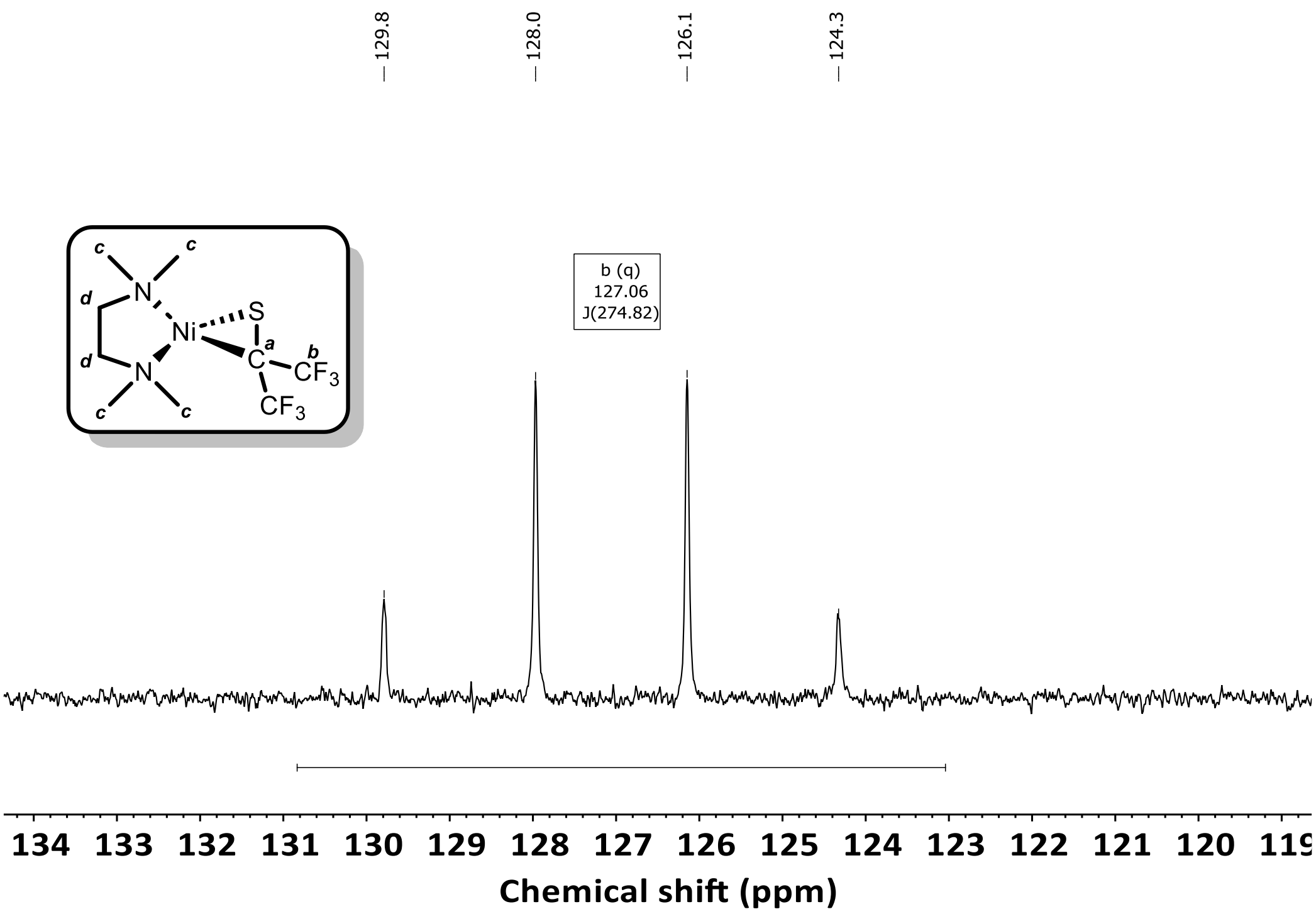


Figure S 22: Enlargement of signal **b** at 127.1 ppm of the $^{13}C\{^1H\}$-NMR spectrum of **5**.

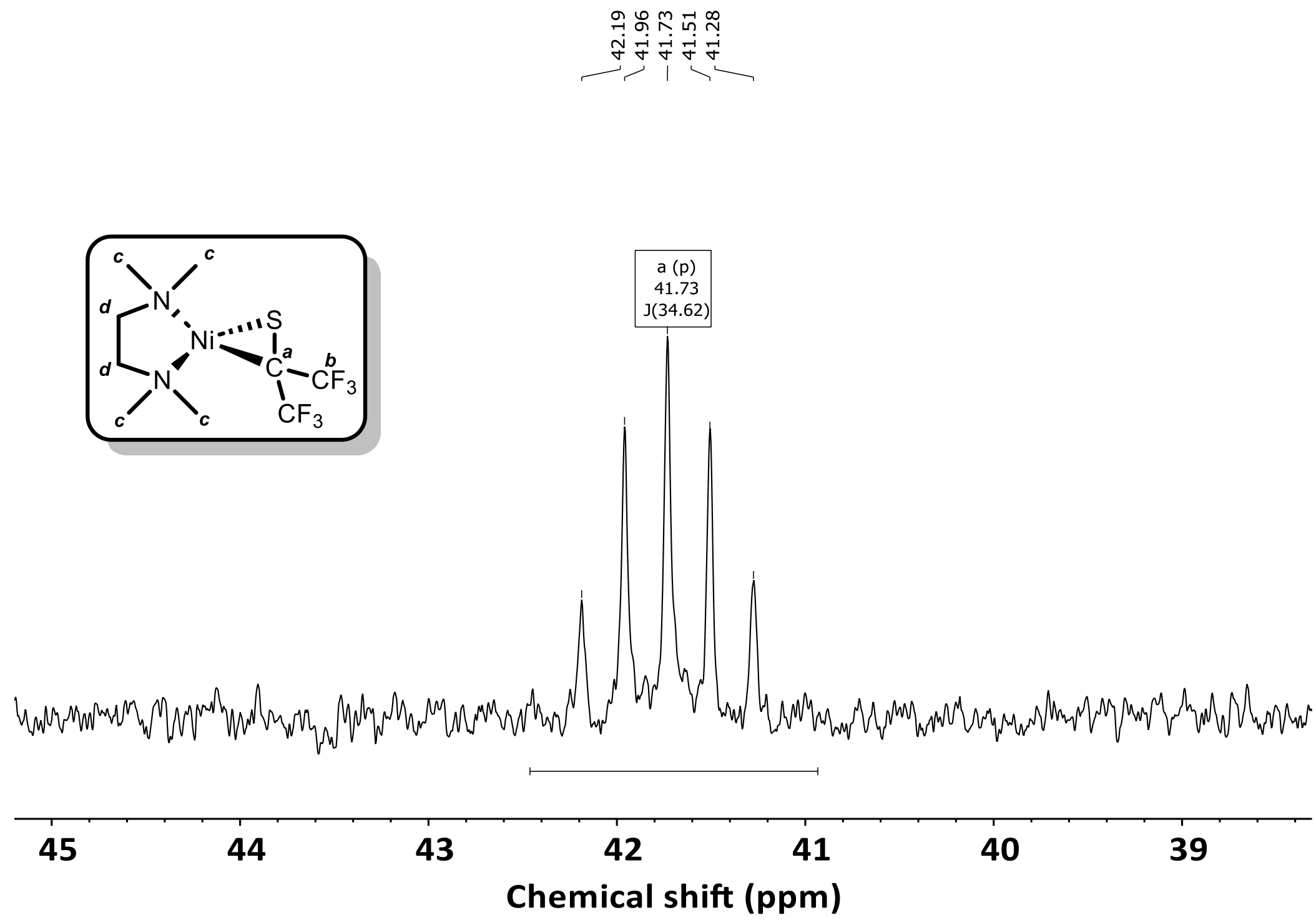


Figure S 23: Enlargement of signal **a** at 41.7 ppm of the $^{13}C\{^{1}H\}$-NMR spectrum of **5**.

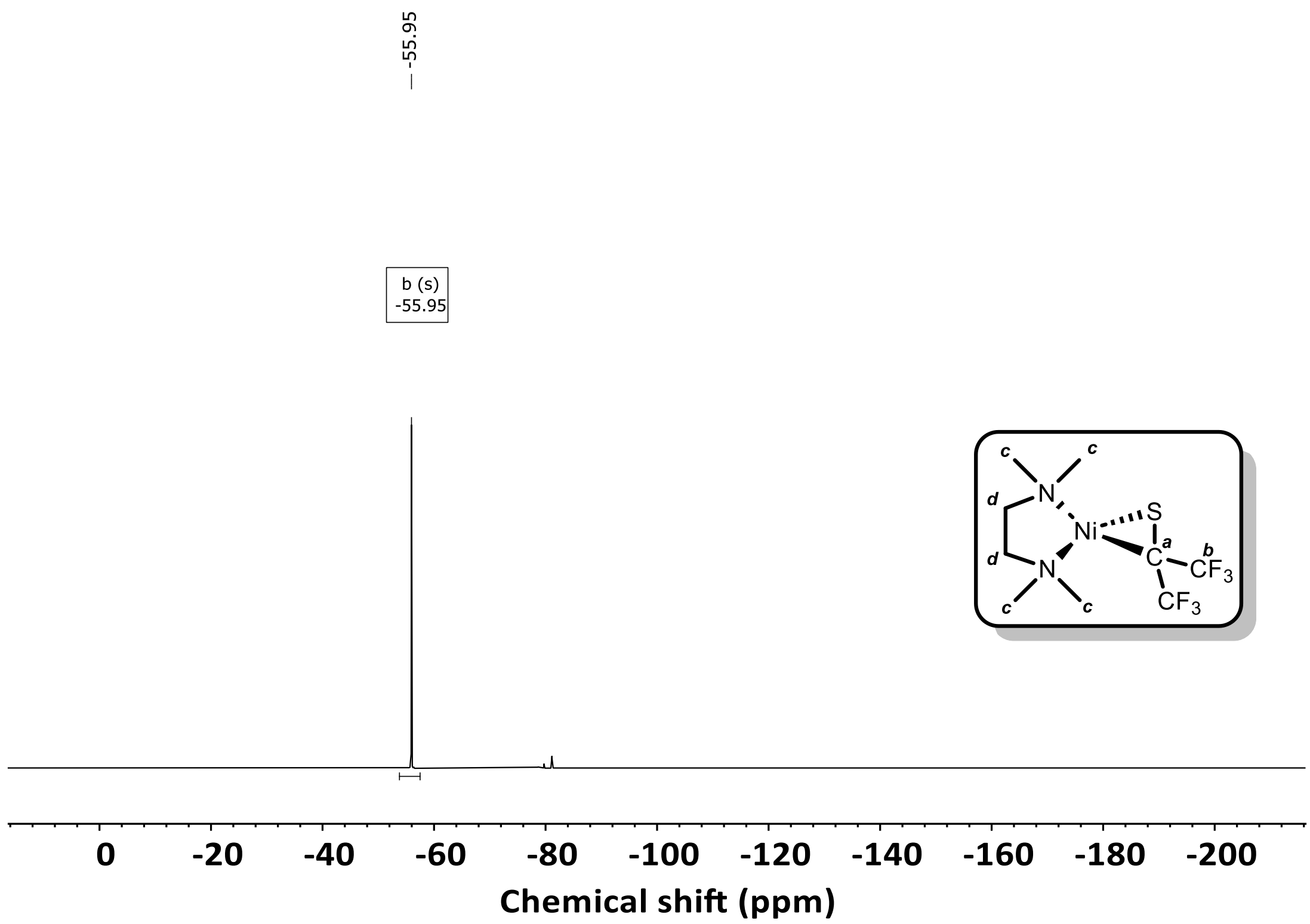


Figure S 24: $^{19}F$-NMR spectrum of **5** in $C_6D_6$ at rt.

## 1.6 NMR spectra of 2,2,4,4-Tetrakis(trifluormethyl)-1,3-dithietan (2)

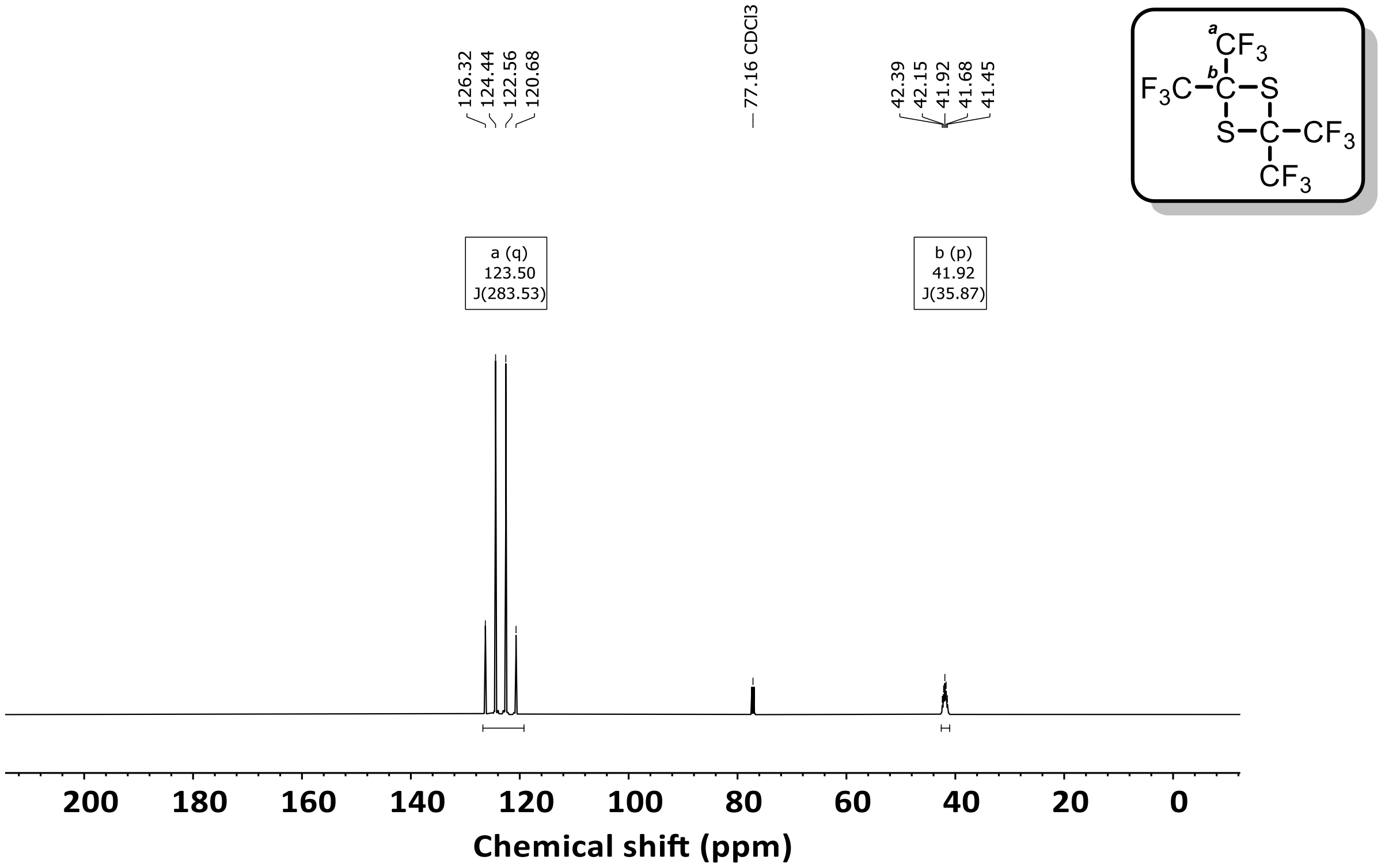


Figure S 25: $^{13}$C-NMR spectrum of **2** in $CDCl_3$ at rt.

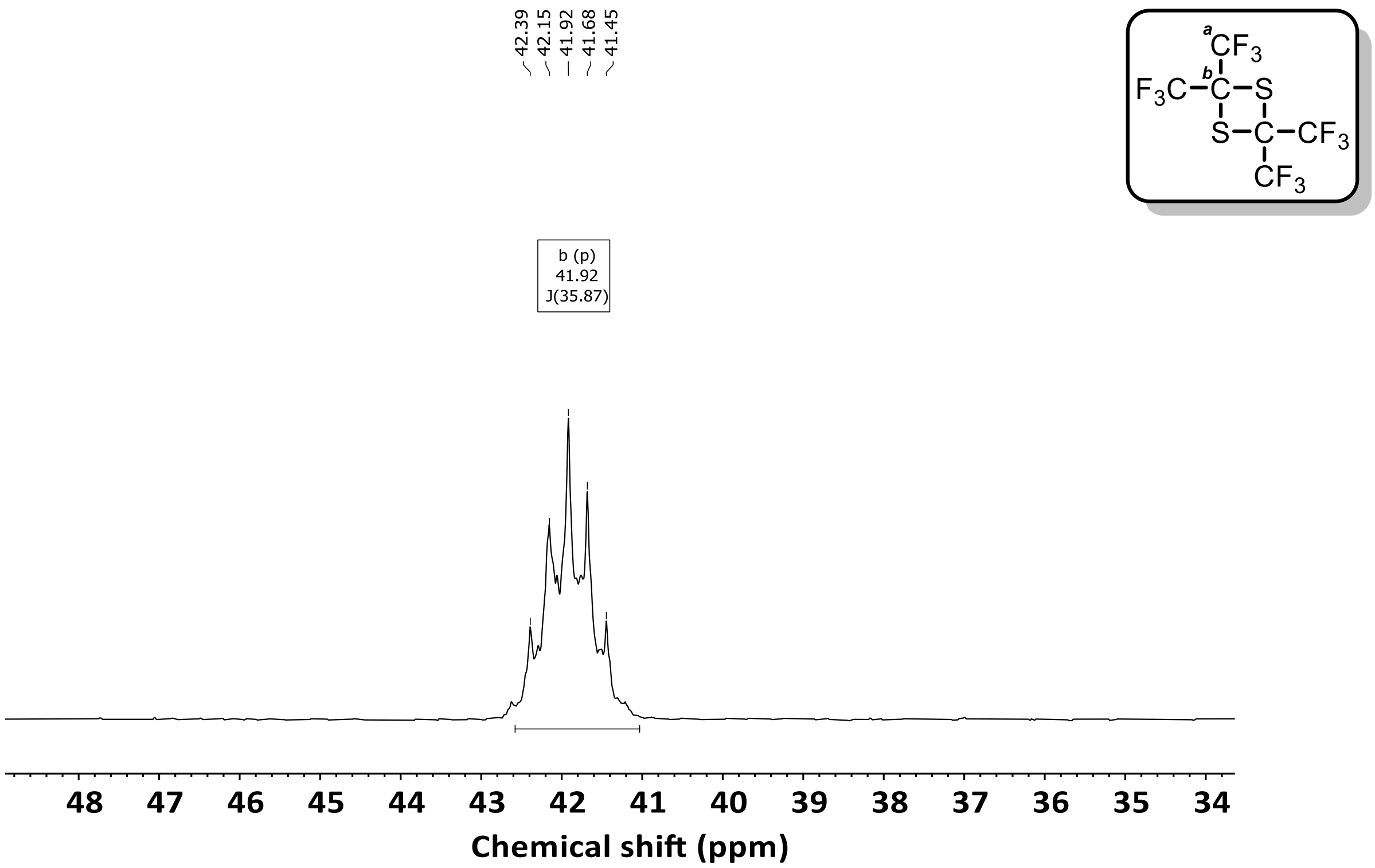


Figure S 26: Enlargement of the signal at 41.92 ppm of the $^{13}$C-NMR of **2**.

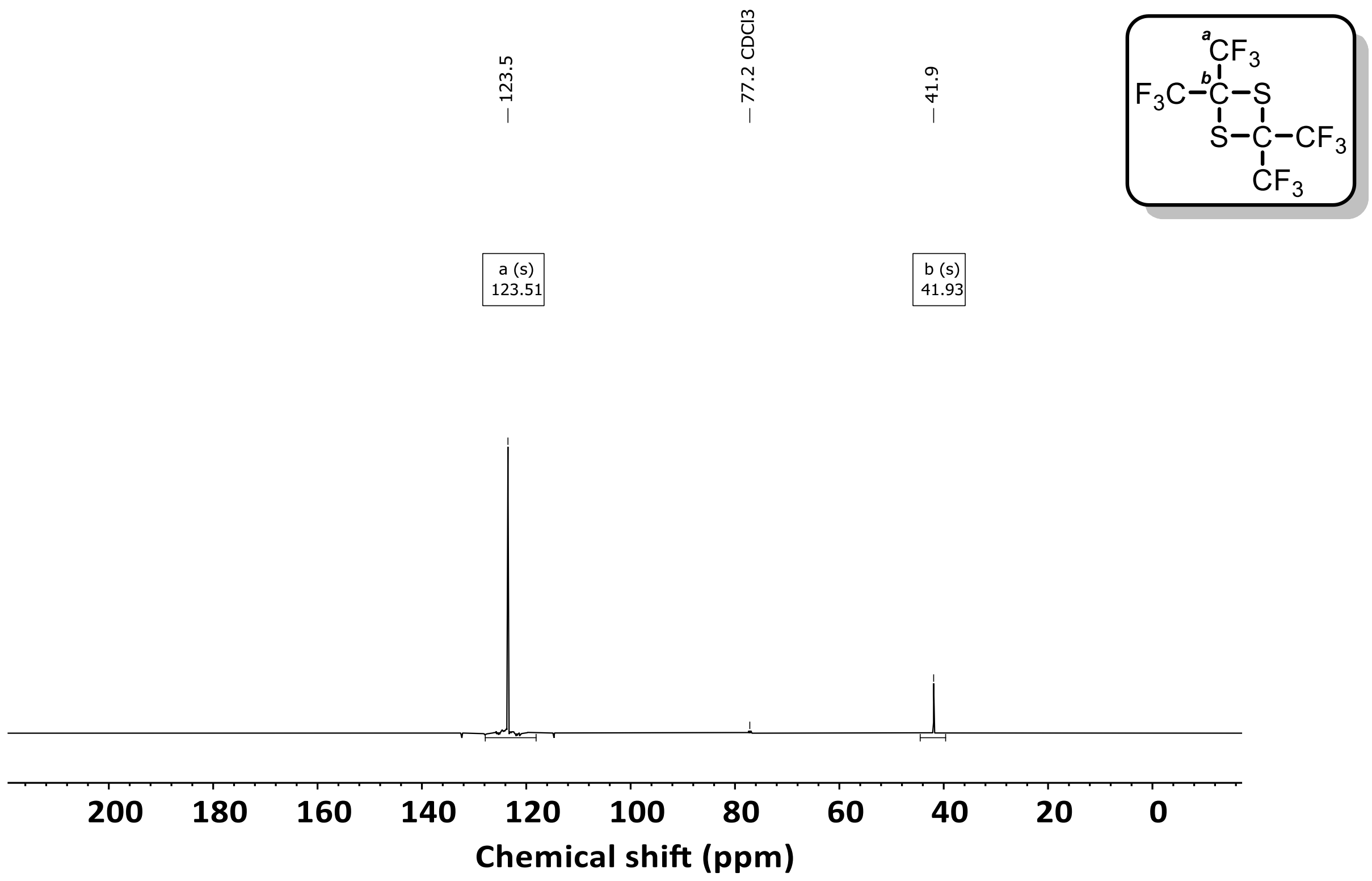


Figure S 27: Measured $^{13}C\{^{19}F\}$-NMR spectrum of **2** in $CDCl_3$ at rt.

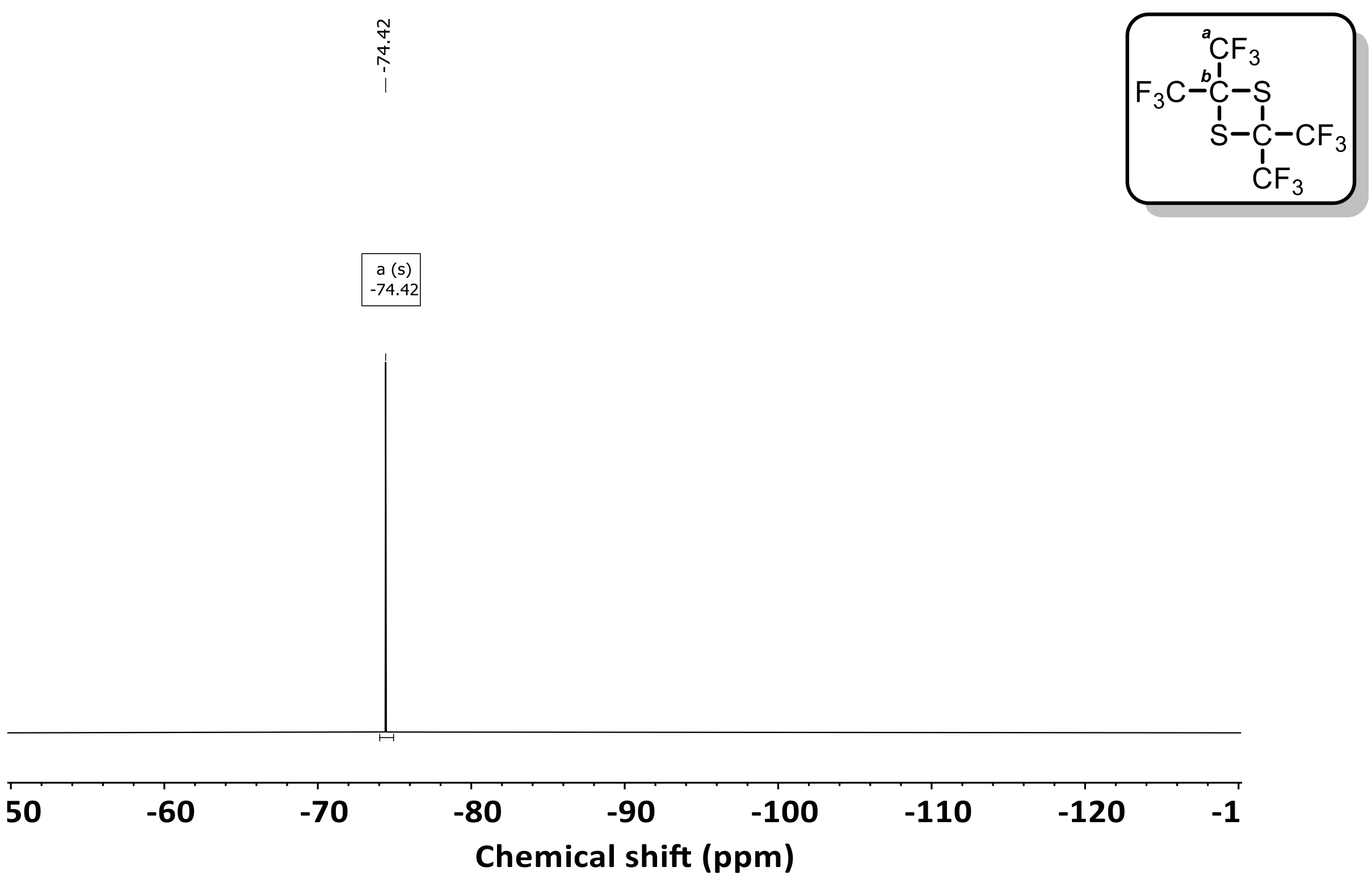


Figure S 28: Measured $^{19}F$-NMR spectrum of **2** in $CDCl_3$ at rt.

## 2. Crystallographic data

Single-crystal X-ray diffraction data were collected using an StadiVari (Stoe, Darmstadt) IPDS-2T (Stoe, Darmstadt) and QuestD8 (Bruker, Billerica) diffraction system equipped with mirror monochromated Mo $K_\alpha$ radiation ($\lambda$ = 0.7107 Å, Xenocs Microfocus Source), and Cu $K_\alpha$ radiation ($\lambda$ = 1.54186 Å, Xenocs Microfocus Source) and an Image Plate detector. Crystals were selected under Paratone-N oil, mounted on micromount loops, and quench-cooled using an Oxford Cryosystems open-flow $N_2$ cooling device. Data were collected at 100 K and processed using the X-Area program package, including unit-cell parameter refinement and interframe scaling (which was carried out using LANA within X-Area) or Apex3 software.[7]

The structures were subsequently solved using direct dual-space methods (SHELXT)[8] and refined on $F^2$ with SHELXL[9] using the Olex2[10] user interface. The crystal structure drawings were generated with DIAMOND.[11]

## 2.1 Experimental details on single crystal X-ray structure determination of 1

**Experimental setup**: Reservoir flask of **1** was connected to a Schlenk line and a capillary Schlenk line attachment *via* three-way valve. A capillary (diameter 0.3 mm, Lindemann Spezialglas) was glued with picein to the capillary attachment. (Figure S 29). To mount the capillary onto the goniometer of the StadiVari, it was further glued in the capillary holder using picein (Figure S 30).

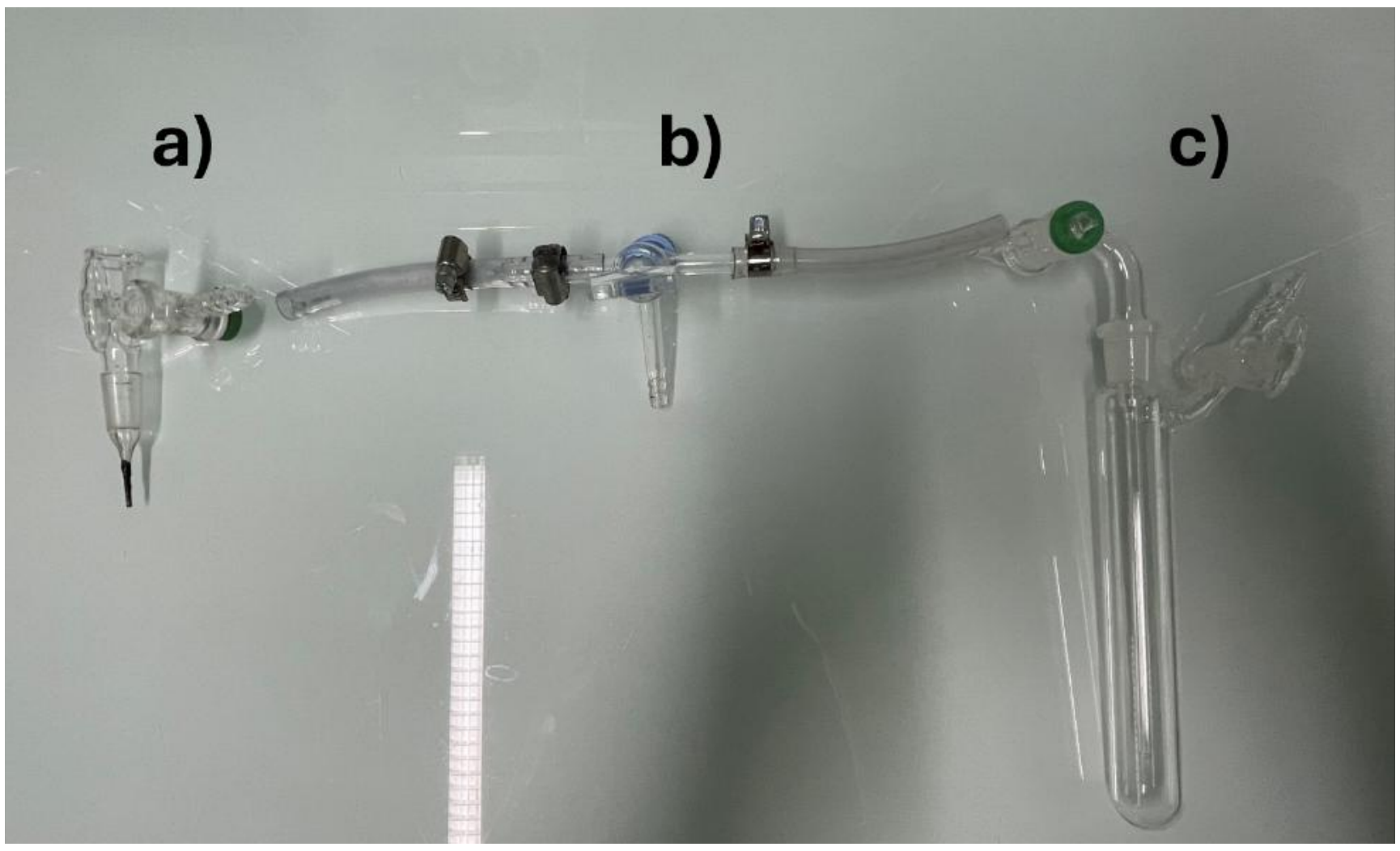


Figure S 29: Experimental setup for condensation of **1** into a capillary suitable for single-crystal structure analysis. Inert gas inlet with fixed (glue: picein) capillary (**a**). Three-way valve (**b**). Reservoir flask of **1** (**c**)

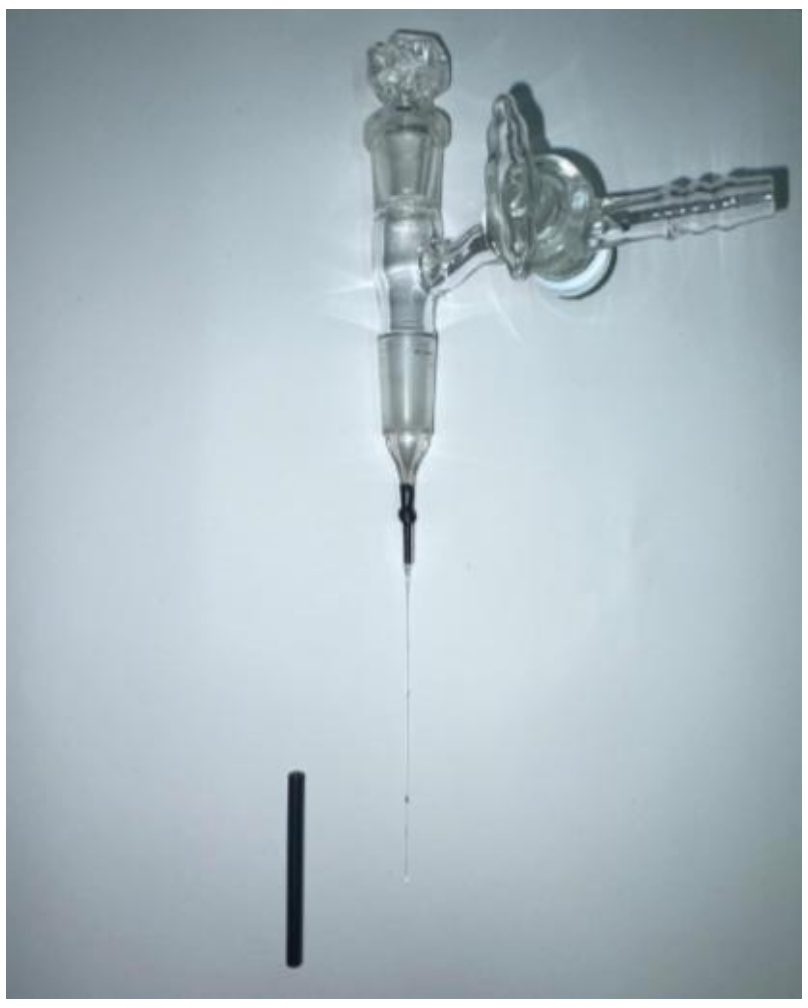

Figure S 30: Capillary holder (left) and capillary with Schlenk line attachment (right).

**Experimental procedure:** First the reservoir flask of **1** was cooled with liquid nitrogen for approx. 5 min under static argon atmosphere. Then vacuum was applied ($5\times10^{-3}$ bar). Afterwards the system was sealed, and the capillary holder was immersed about half a centimeter into liquid nitrogen, while the reservoir of **1** was removed from liquid nitrogen. Over the course of 10 minutes the capillary was slowly immersed into liquid nitrogen until about one centimeter of the condensed blue solid **1** was visible out of the top of the capillary holder. Then the reservoir container of **1** was cooled with liquid nitrogen and the system was evacuated. The capillary was pulled out of the liquid nitrogen and quickly flame sealed with an electric arc-lighter. With this procedure, the capillary seals itself due to the applied vacuum. The weight of the capillary holder pulls the flame-sealed capillary down into the liquid nitrogen, immediately cooling the substance.

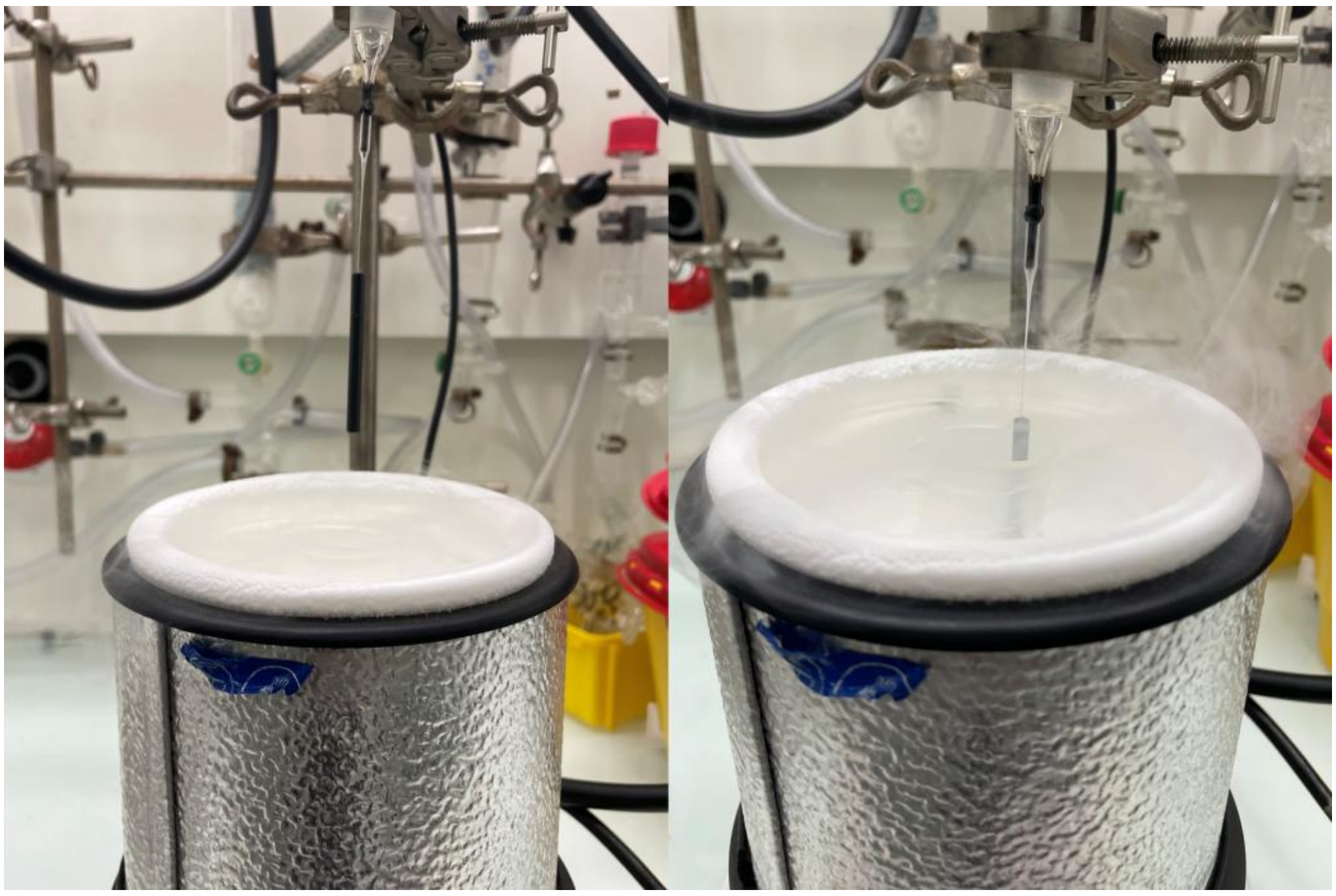

Figure S 31: Inert gas inlet with fixed (glue: picein) capillary before condensation (left) and during the condensation (right).

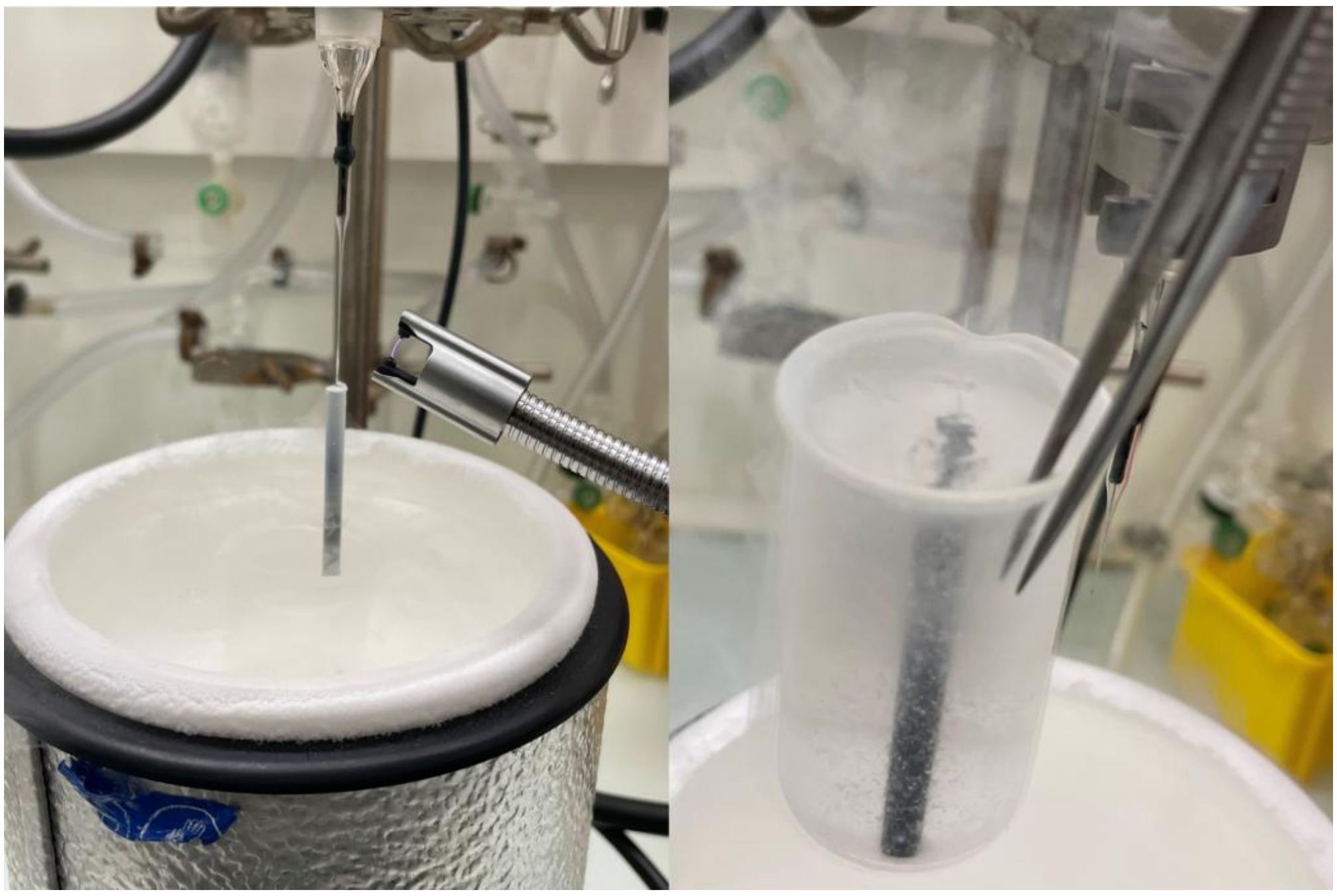

Figure S 32: Capillary with holder just before flame sealing with electric lighter (left) and vacuum sealed capillary in a beaker filled with liquid nitrogen (right, ***note****: the images are solely for visualizing the work steps and were staged, which is why there is no blue solid in the capillary.)*

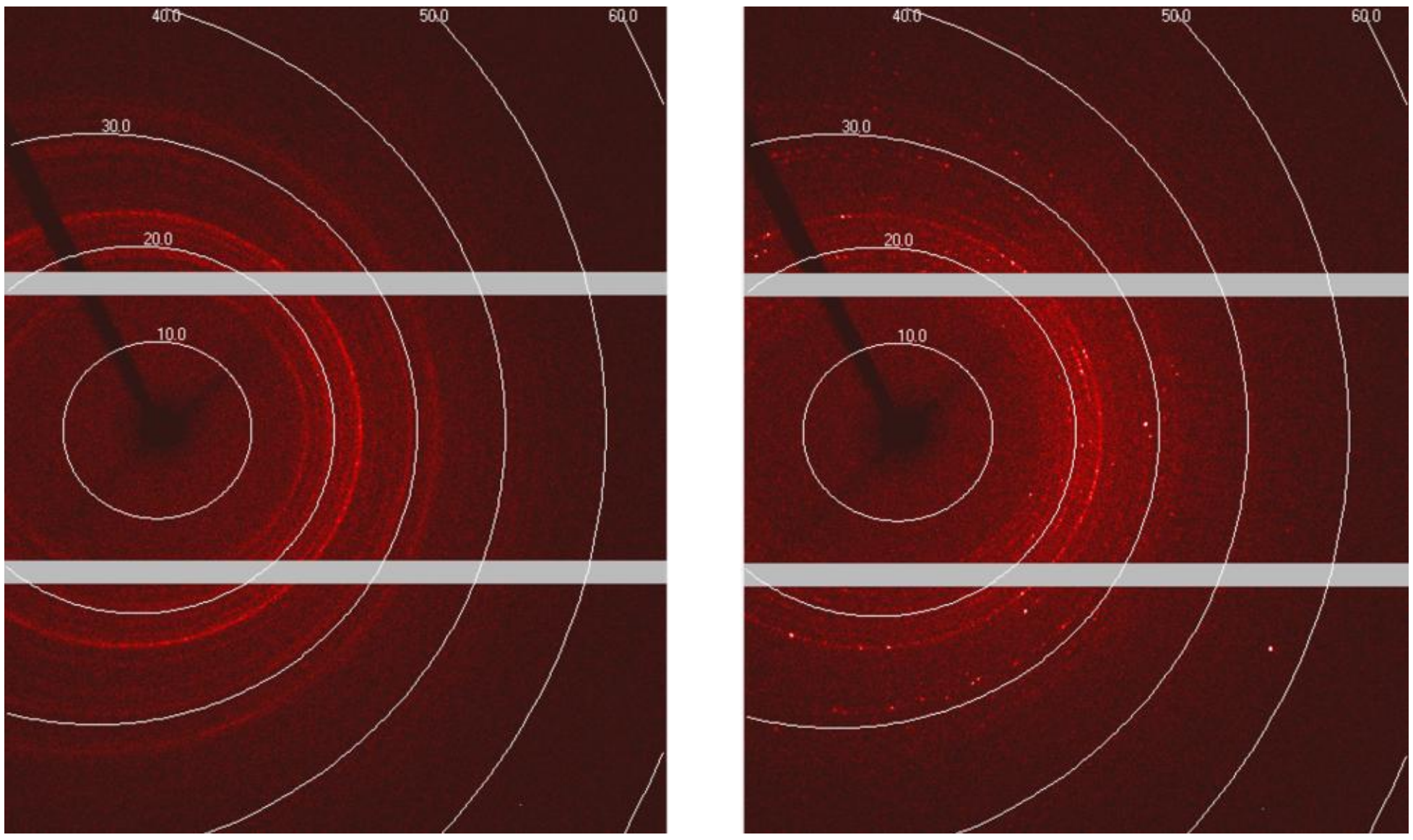


Figure S 33: Test measurement of **1** after immediate solidification at 100 K (left) and after melting and slow cooling to 100 K.

## 2.2 Single crystal X-ray data collection and refinement parameters

Table S 1: Selected single crystal X-ray data collection and refinement parameters for **1**, **2**, **3**, **4,** and **5**.

| | **1** | **2 (285 K)** | **2 (100 K)** | **3 (alpha)** | **3 (beta)** | **4** | **5** |
|---|---|---|---|---|---|---|---|
| Formula | C3 F6 S | C6 F12 S2 | C6 F12 S2 | C11 H12 F6 Ni S | C11 H12 F6 Ni S | C13 H10 F6 N2 Ni S | C9 H16 F6 N2 Ni1 S1 |
| CCDC | 2526808 | 2526810 | 2526807 | 2526813 | 2526811 | 2526812 | 2526809 |
| F. w. / g mol$^{-1}$ | 182.09 | 364.18 | 364.18 | 348.98 | 348.98 | 399.00 | 357.01 |
| Crystal system | monoclinic | monoclinic | monoclinic | triclinic | triclinic | monoclinic | monoclinic |
| Space group | $C2/c$ | $P2_1/n$ | $P2_1/n$ | $P\bar{1}$ | $P\bar{1}$ | $P2_1/n$ | $Pna2_1$ |
| $a$ / Å | 11.6099(5) | 6.734(3) | 6.6082(4) | 6.8045(4) | 6.7425(2) | 9.0624(2) | 15.7891(10) |
| $b$ / Å | 6.3249(2) | 9.872(3) | 9.7165(4) | 9.0265(6) | 8.0537(3) | 11.2389(2) | 8.2938(5) |
| $c$ / Å | 32.3849(14) | 8.795(4) | 8.5973(5) | 11.1328(7) | 11.6230(5) | 14.9956(3) | 10.8927(6) |
| $\alpha$ / ° | 90 | 90 | 90 | 102.444(2) | 89.621(3) | 90 | 90 |
| $\beta$ / ° | 91.893(4) | 108.45(3) | 108.372(5) | 93.087(2) | 86.966(3) | 101.127(2) | 90 |
| $\gamma$ / ° | 90 | 90 | 90 | 110.015(2) | 80.872(3) | 90 | 90 |
| $V$ / Å$^3$ | 2376.77(16) | 554.6(4) | 523.88(5) | 621.30(7) | 622.29(4) | 1498.61(5) | 1426.42(15) |
| $Z$ | 16 | 2 | 2 | 2 | 2 | 4 | 4 |
| Radiation, $\lambda$ / Å | 1.54186 (Cu K$_\alpha$) | 1.54186 (Cu K$_\alpha$) | 1.54186 (Cu K$_\alpha$) | 0.71073 (Mo K$_\alpha$) | Cu K$_\alpha$ ($\lambda$ = 1.54186) | Cu K$_\alpha$ ($\lambda$ = 1.54186) | 0.71073 (Mo K$_\alpha$) |
| Temp / K | 100 | 285 | 100 | 100 | 100 | 100 | 100 |
| $\rho_{calc}$ / g cm$^{-3}$ | 2.035 | 2.181 | 2.309 | 1.865 | 1.862 | 1.768 | 1.662 |
| $\mu$ / mm$^{-1}$ | 5.539 | 5.934 | 6.282 | 1.785 | 4.408 | 3.801 | 1.560 |
| Reflections collected | 14707 | 9641 | 4522 | 53745 | 16373 | 31815 | 5240 |
| Ind. Reflns. / Ind. Reflns gt | 2124 / 1632 | 1080 / 831 | 1074 / 1058 | 4488 / 3823 | 2137 / 1780 | 2700 | 2934 / 2148 |
| Parameters | 236 | 93 | 92 | 210 | 172 | 2046 | 177 |
| $R_{int}$ / $R_{(\sigma)}$ / % | 7.94 / 3.98 | 6.71 / 2.86 | 2.55 / 1.53 | 12.34 / 7.24 | 6.38 / 2.80 | 7.58 / 3.79 | 5.38 / 7.01 |
| $R1$/$wR2$,[a] $I \geq 2\sigma I$ / % | 7.03/ 19.17 | 4.01 / 9.98 | 3.41 / 9.41 | 3.22 / 7.07 | 6.45 / 18.72 | 2.82 / 5.64 | 3.18 / 5.58 |
| $R1$/$wR2$,[a] all data / % | 9.09/ 21.03 | 5.36 / 10.39 | 3.44 / 9.45 | 5.80 / 7.29 | 7.81 / 20.06 | 4.80 / 5.99 | 5.72 / 5.93 |
| GOF | 1.062 | 0.944 | 1.053 | 1.082 | 1.074 | 0.931 | 0.887 |
| Twin Law | – | – | – | – | – | – | −1 0 0 0 −1 0 0 0 −1 |
| BASF | – | – | – | – | – | – | 0.493 |

[a] $R1 = [\Sigma||F_o| - |F_c||]/\Sigma|F_o|$; $wR2 = \{[\Sigma w[(F_o)^2 - (F_c)^2]^2]/[\Sigma w(F_o^2)^2\}^{1/2}$; $w = [\sigma^2(F_o)^2 + (AP)^2 + BP]^{-1}$, where $P = [(F_o)^2 + 2(F_c)^2]/3$ and the A and B values are 0.1324 and 6.0245 for **1**, 0.0754 and 0 for **2**(285 K), 0.0606 and 0.2950 for **2**(100 K)0.0246 and 0.2873 for **3**(alpha), 0.1383 and 1.1302 for **3**(beta), 0.0323 and 0 for **4**, 0.0261 and 0 for **5**.

## 2.2 Hirshfeld surface and fingerprint plots for 1

Hirshfeld surface analyses were performed using Crystal Explorer (v25.8).[12,13]

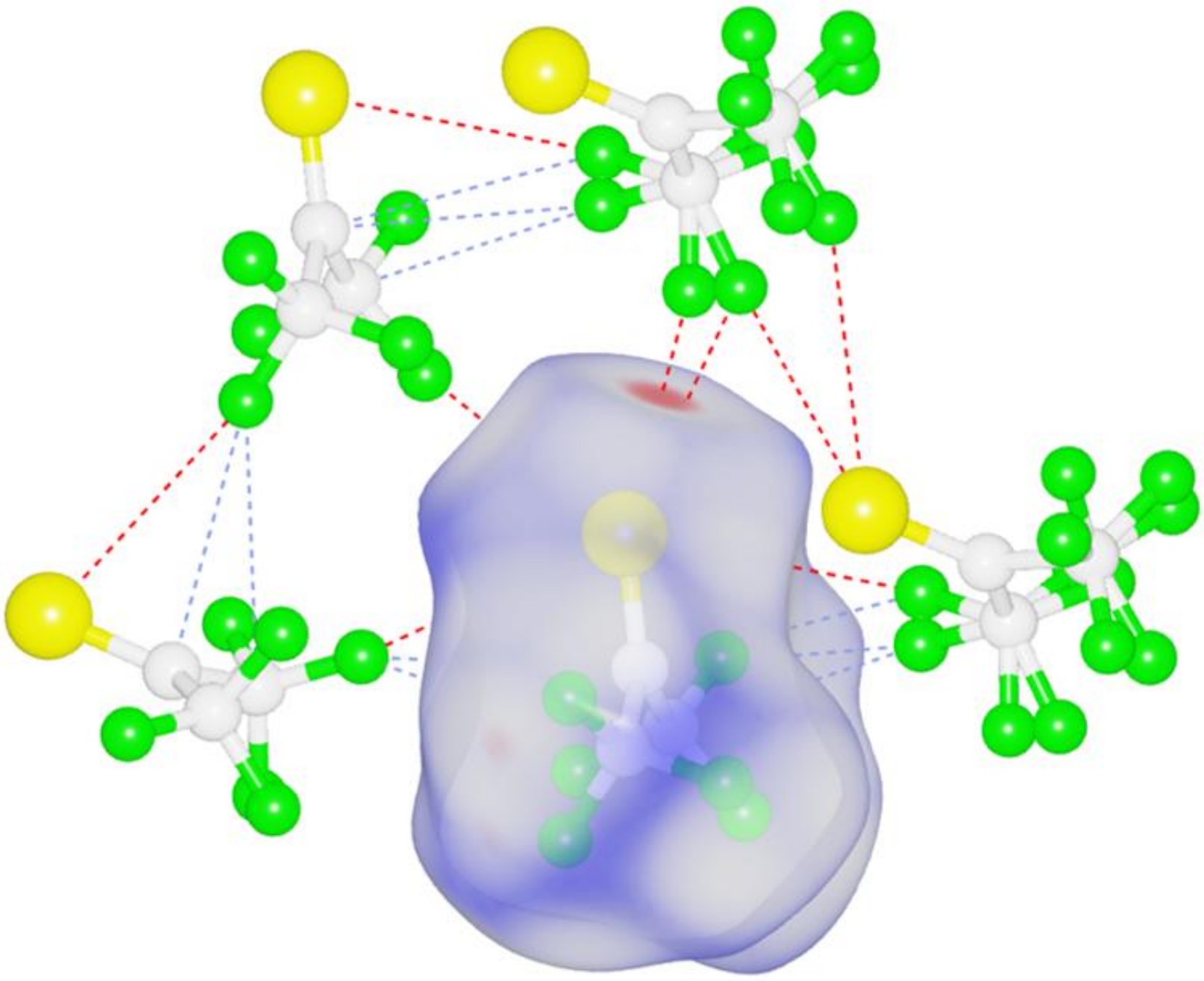

Figure S 34: Hirshfeld surface of **1** shown with selected adjacent molecules. Red areas indicate short contacts. Red dotted lines indicate short S–F contacts and blue dotted lines indicate short C–F contacts. Color code: C white, S yellow, F green.

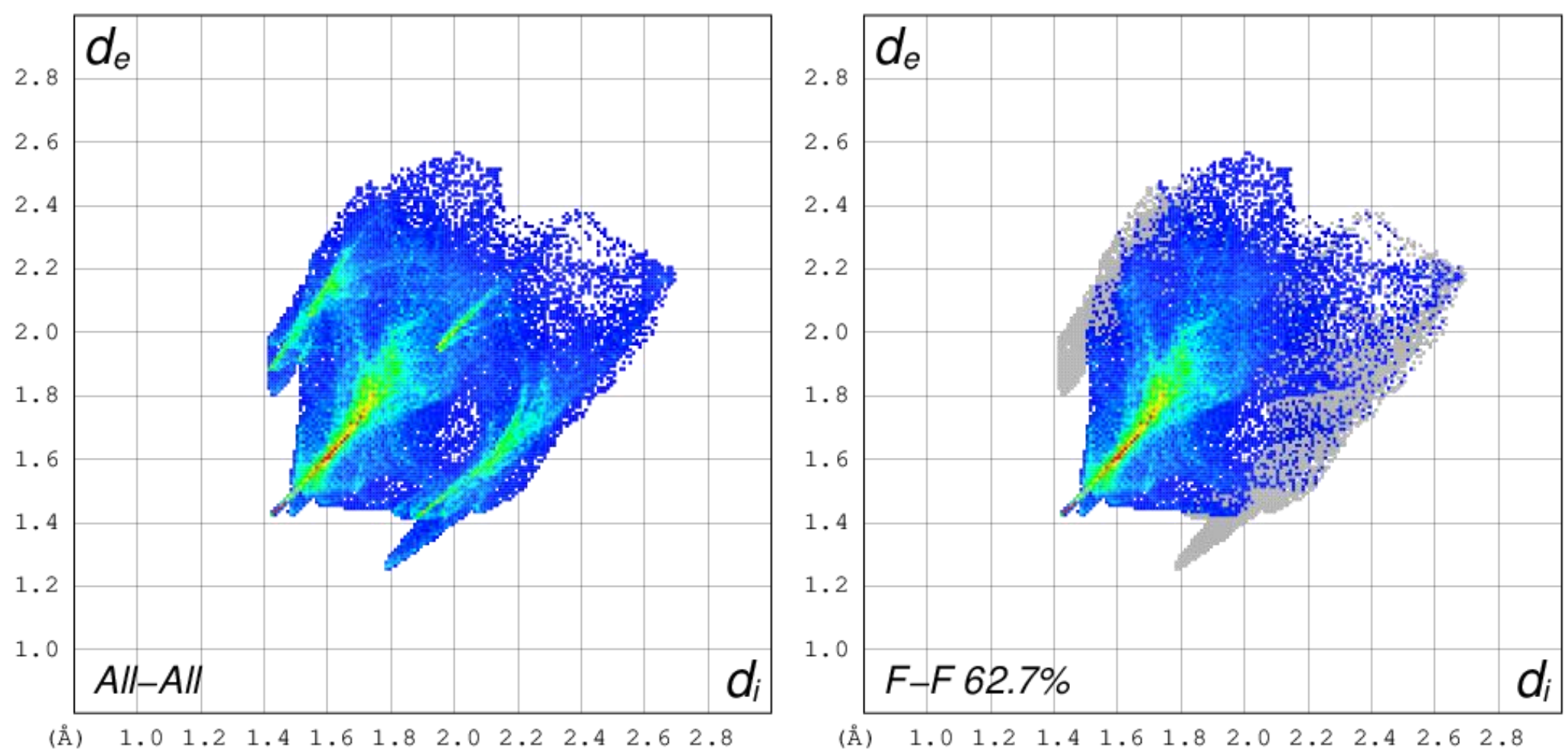


Figure S 35: Fingerprint plots for **1** mapped from the Hirshfeld surface. Left shows all surface contacts, right F–F contacts with their overall amount.

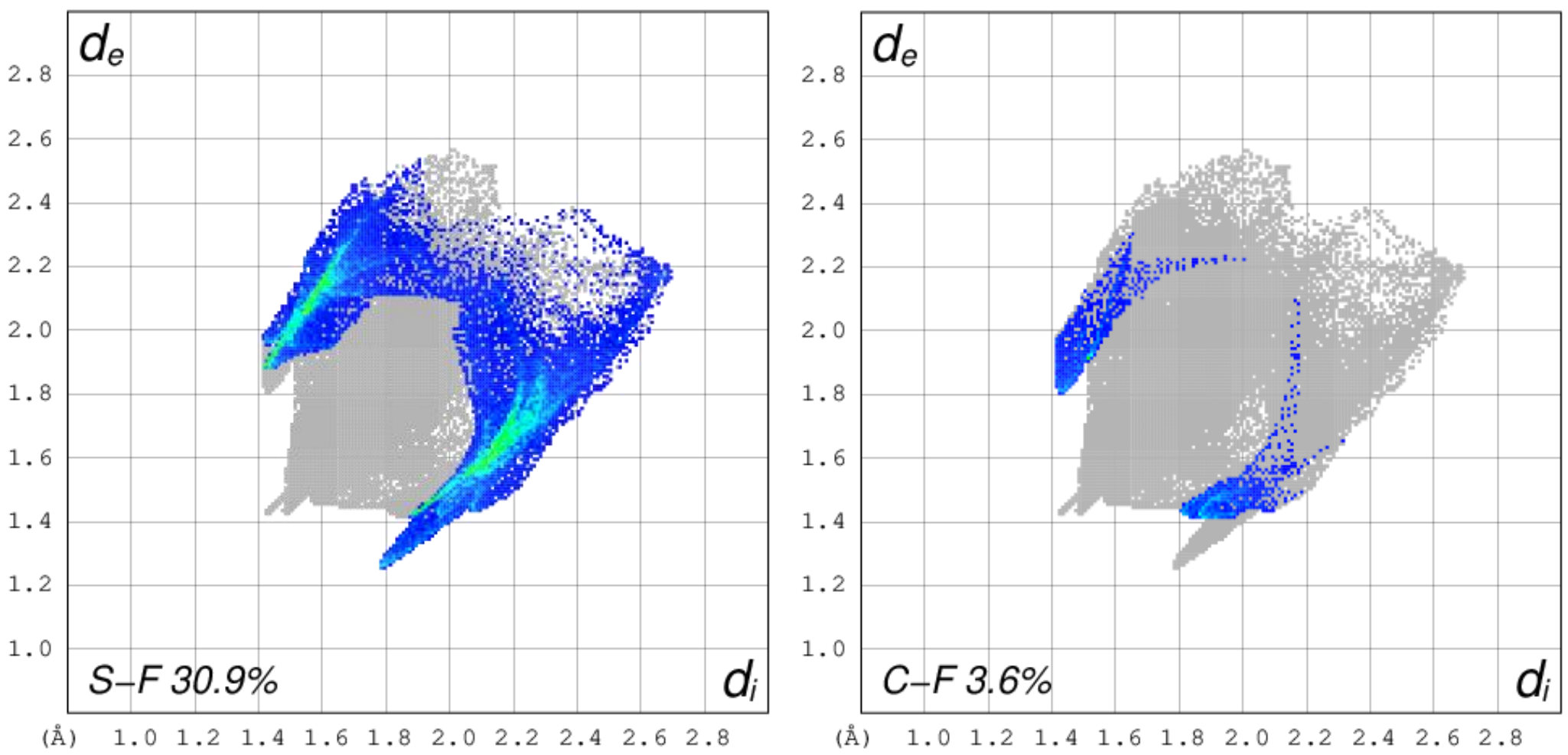


Figure S 36: Fingerprint plots for **1** mapped from the Hirshfeld surface. Left shows S–F contacts, right C–F contacts with their overall amount.

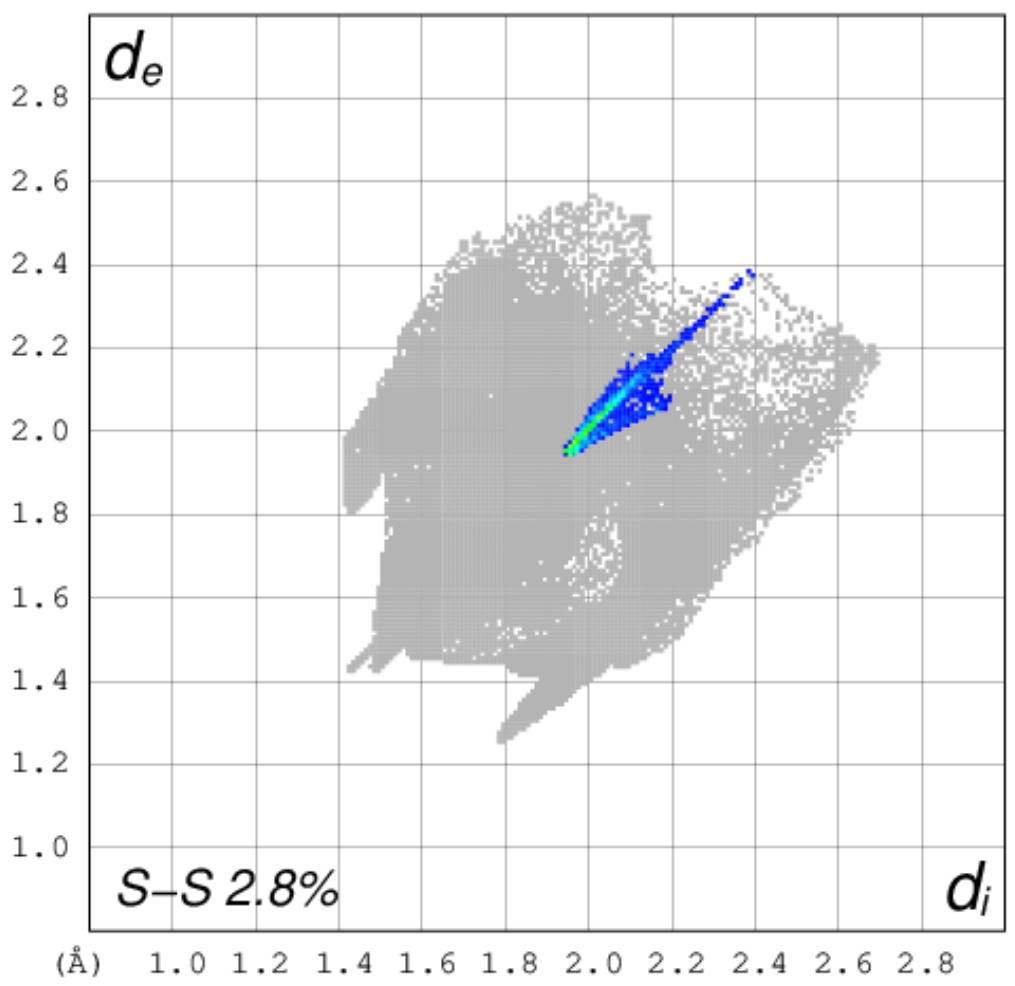


Figure S 37: Fingerprint plot for **1** mapped from the Hirshfeld surface which shows S–F contacts with their overall amount.

## 2.3 Additional Pictures

### 2.3.1 Additional Pictures of 1

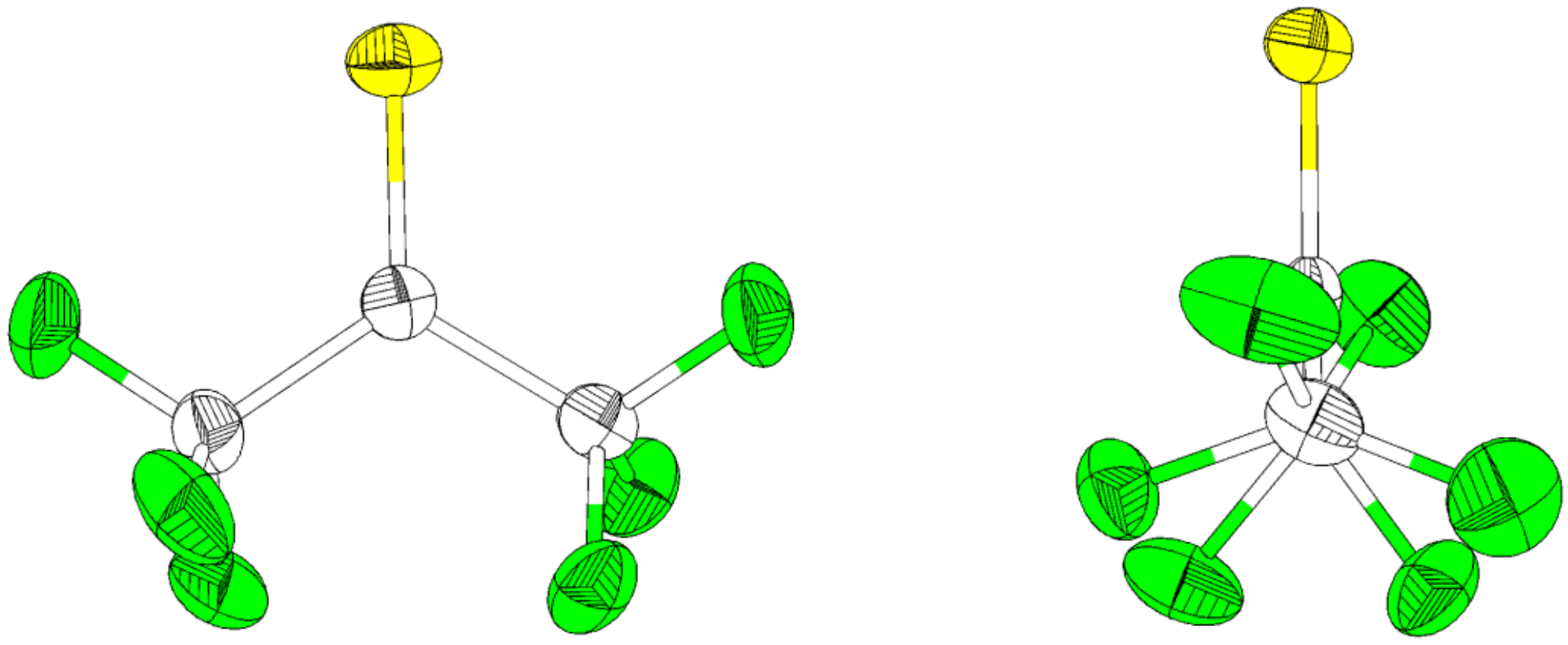

Figure S 38: Structure of the first molecule (no disorder) of **1** in the single crystal (left: front view, right: side view) drawn with 30% displacement ellipsoids at 100 K. Color code: C white, S yellow, F green.

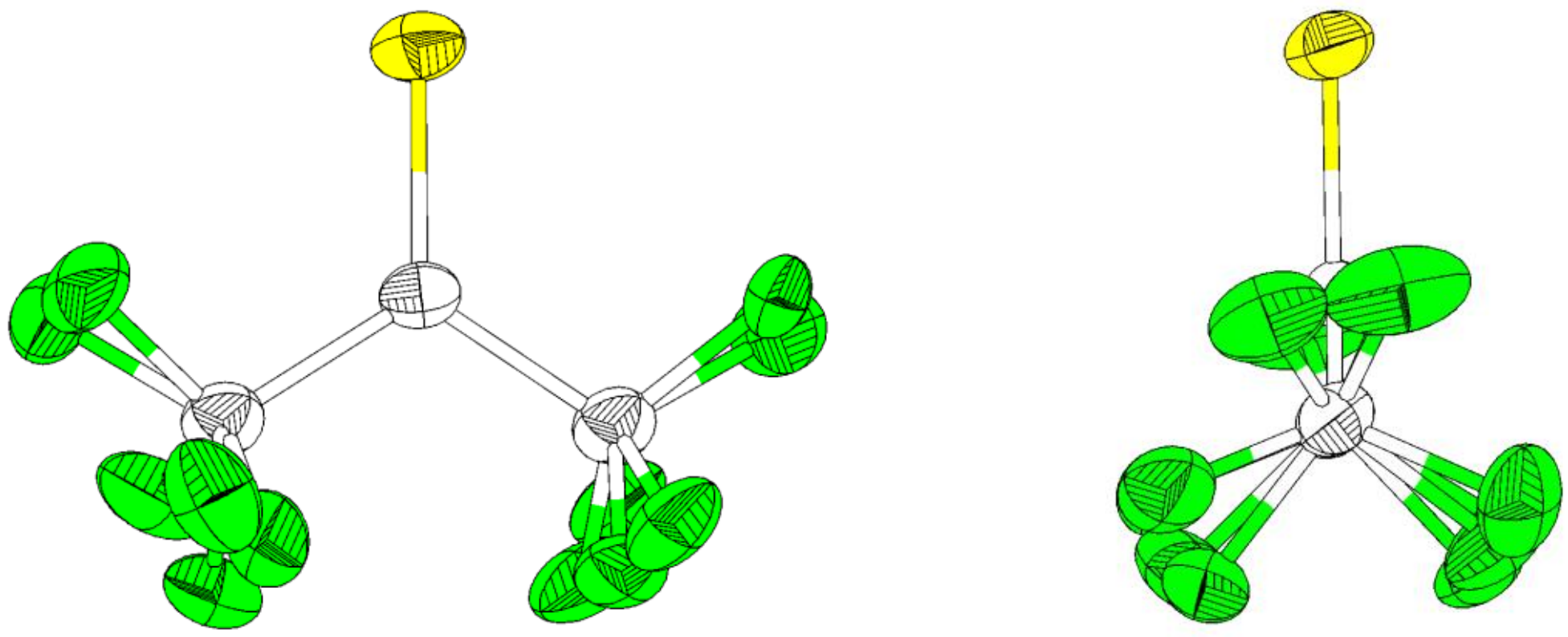

Figure S 39: Structure of second molecule (disordered) of **1** in the single crystal (left: front view, right: side view) drawn with 30% displacement ellipsoids at 100 K. Color code: C white, S yellow, F green.

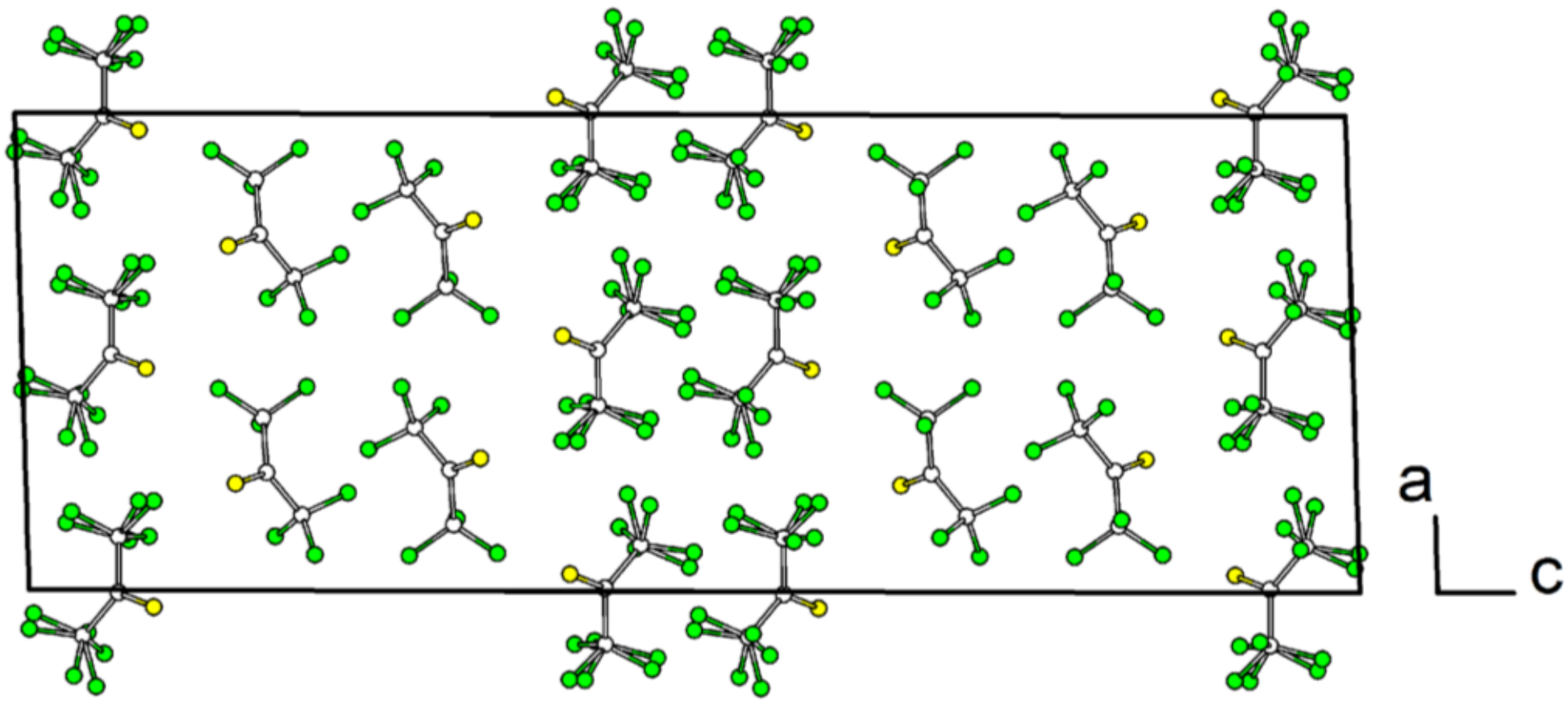


Figure S 40: Crystal structure of **1** viewed along [010]. Atoms drawn with arbitrary radii. Colour code: S yellow, C white, F green.

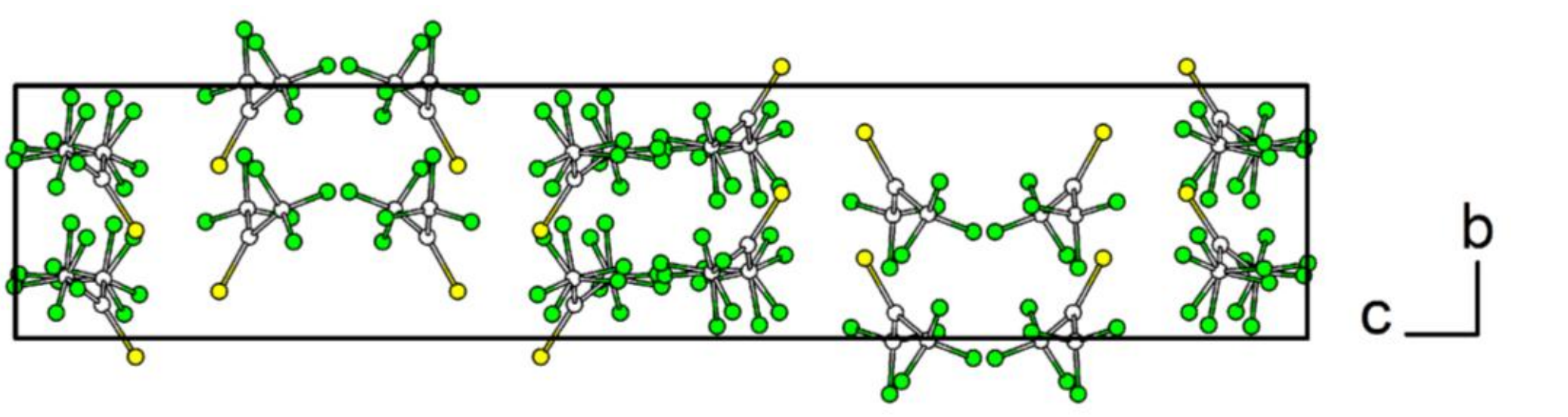


Figure S 41: Crystal structure of **1** viewed along [100]. Atoms drawn with arbitrary radii. Colour code: S yellow, C white, F green.

### 2.3.2 Additional Pictures of 2 (100 K)

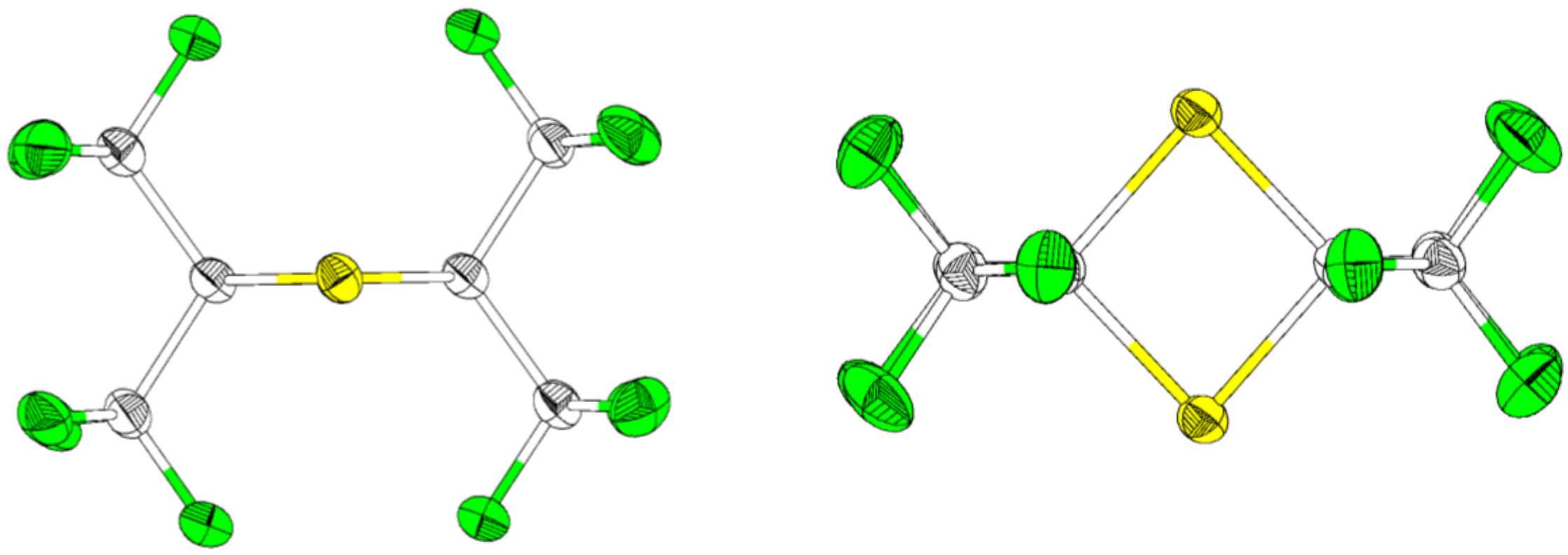

Figure S 42: Structure of **2** (100 K) in the single crystal (left: front view, right: top view) drawn with 50% displacement ellipsoids at 100 K. Color code: C white, S yellow, F green.

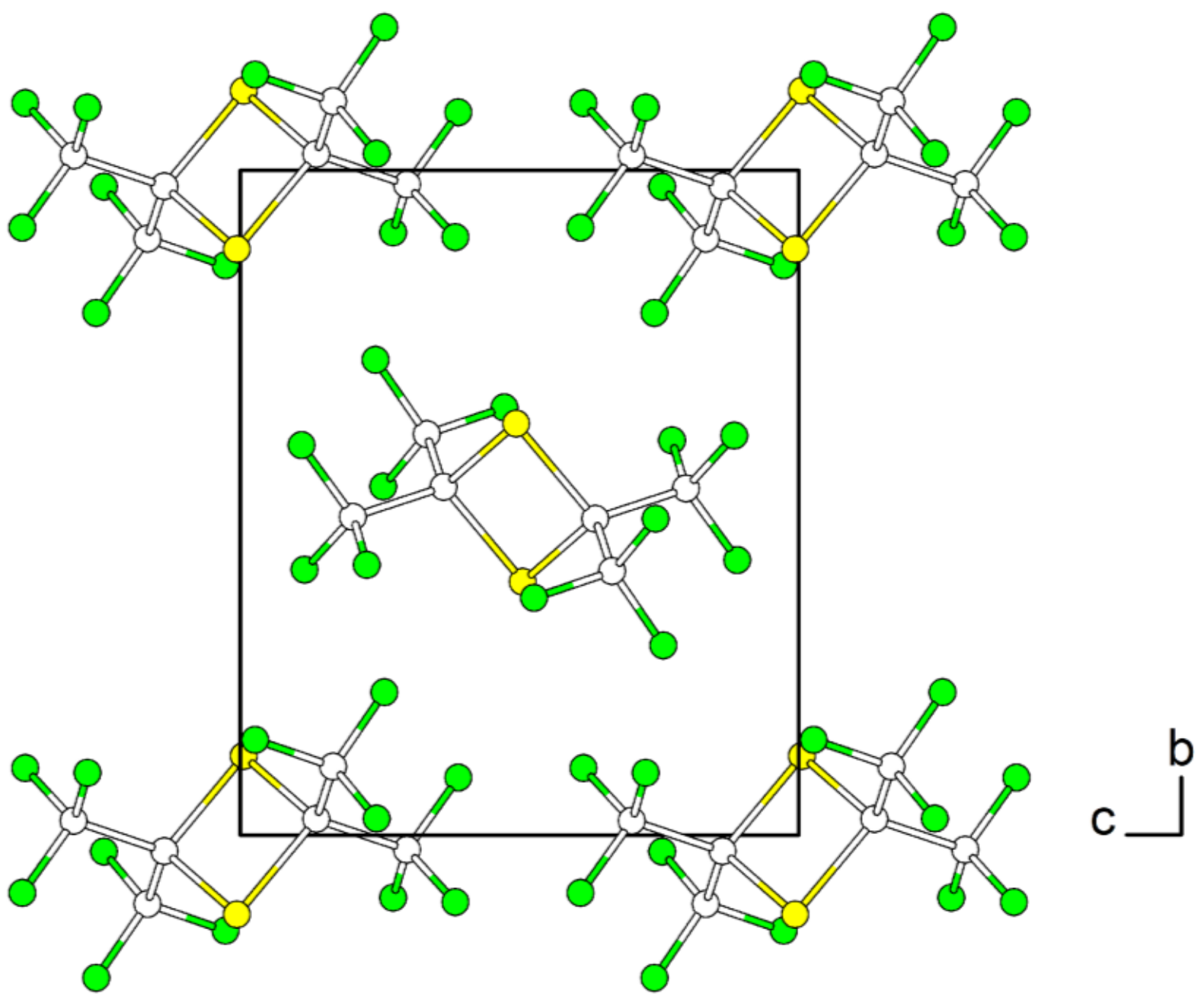


Figure S 43: Crystal structure of **2** viewed along [100]. Atoms drawn with arbitrary radii. Colour code: S yellow, C white, F green.

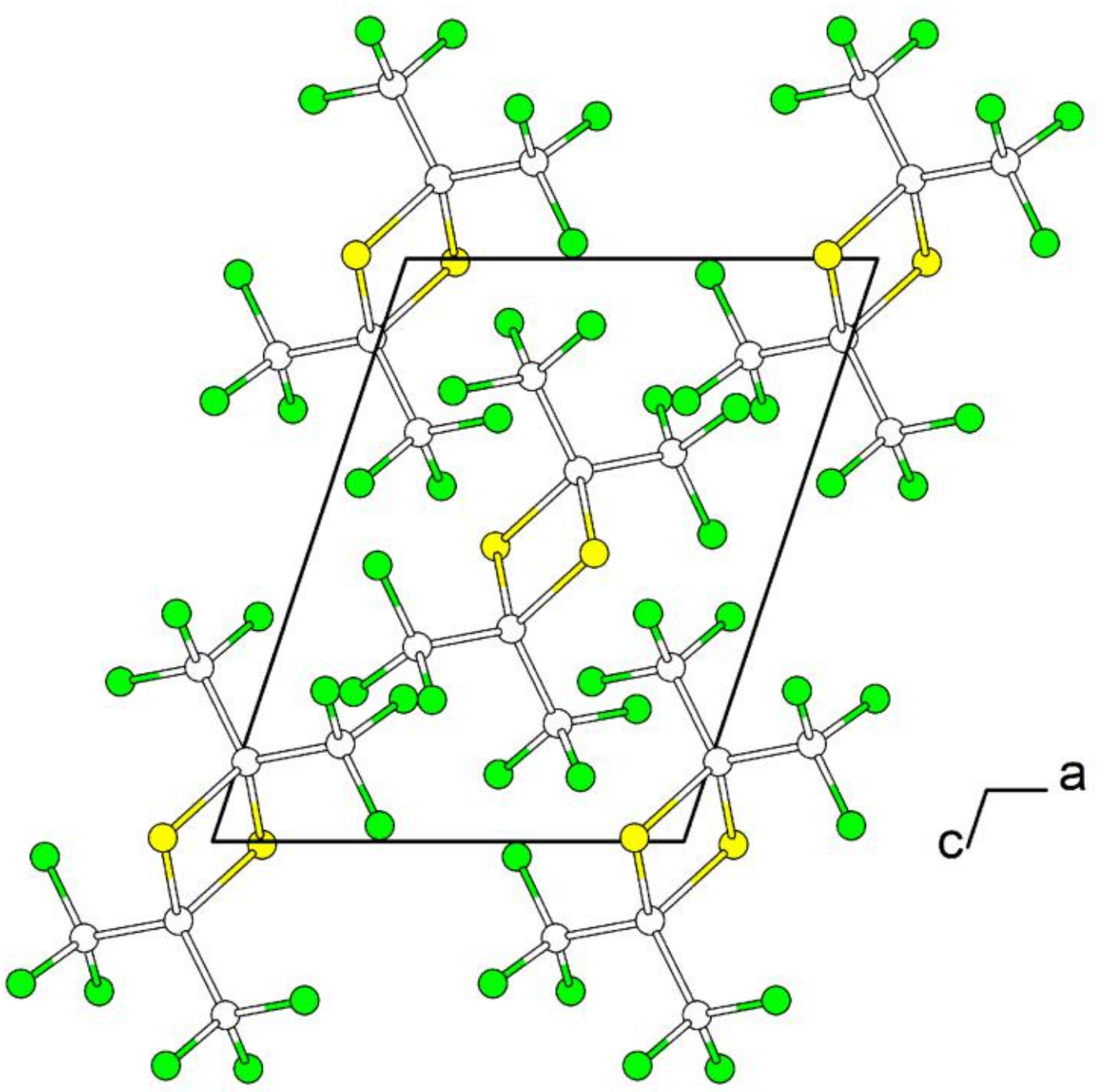


Figure S 44: Crystal structure of **2** viewed along [010]. Atoms drawn with arbitrary radii. Colour code: S yellow, C white, F green.

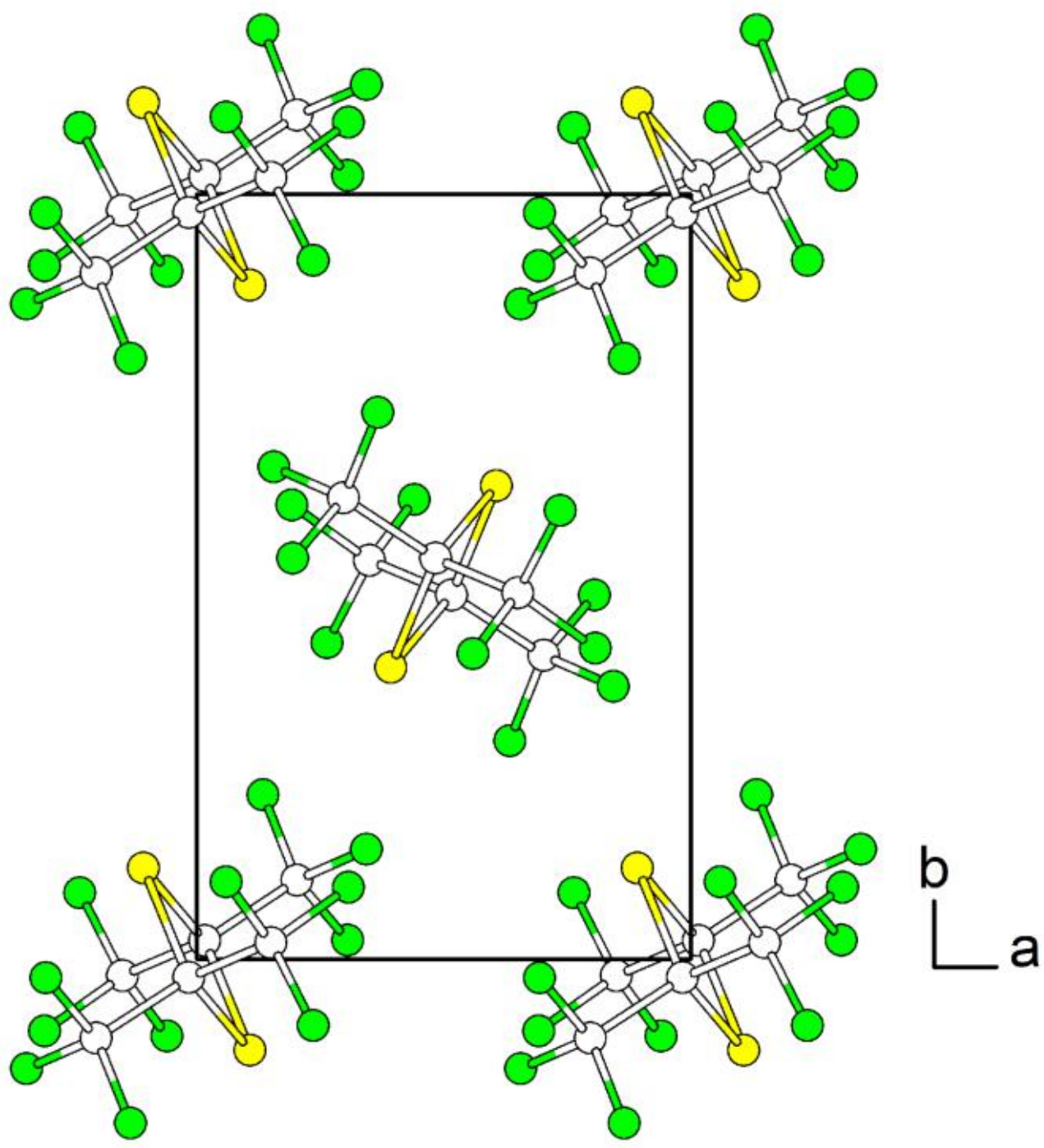


Figure S 45: Crystal structure of **2** viewed along [001]. Atoms drawn with arbitrary radii. Colour code: S yellow, C white, F green.

### 2.3.3 Additional Pictures of 3 (alpha)

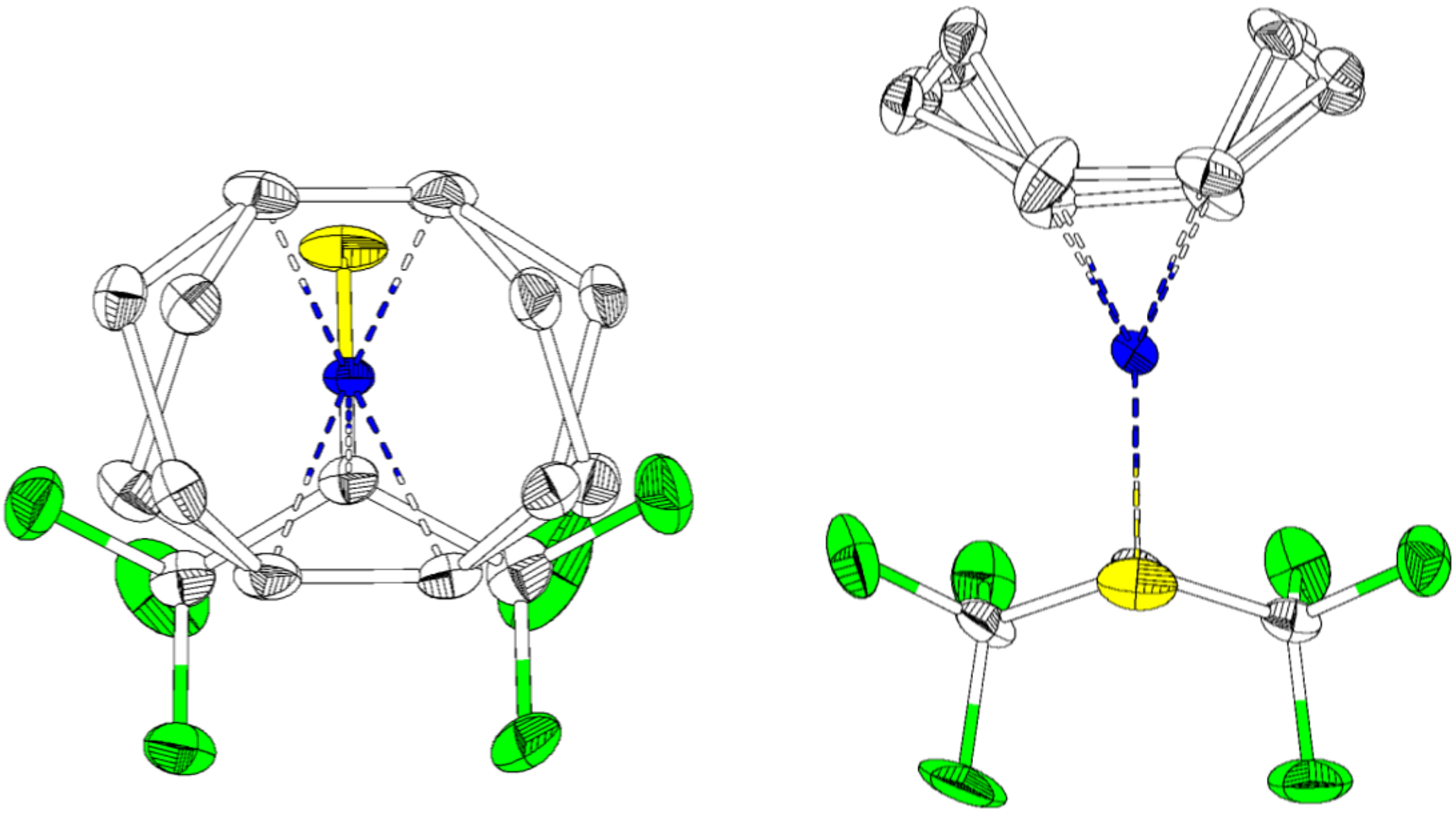

Figure S 46: Structure of **3** (alpha) in the single crystal (left: front view, right: top view) drawn with 50% displacement ellipsoids at 100 K. Hydrogen atoms are omitted for clarity. Color code: C white, S yellow, F green, Ni dark blue.

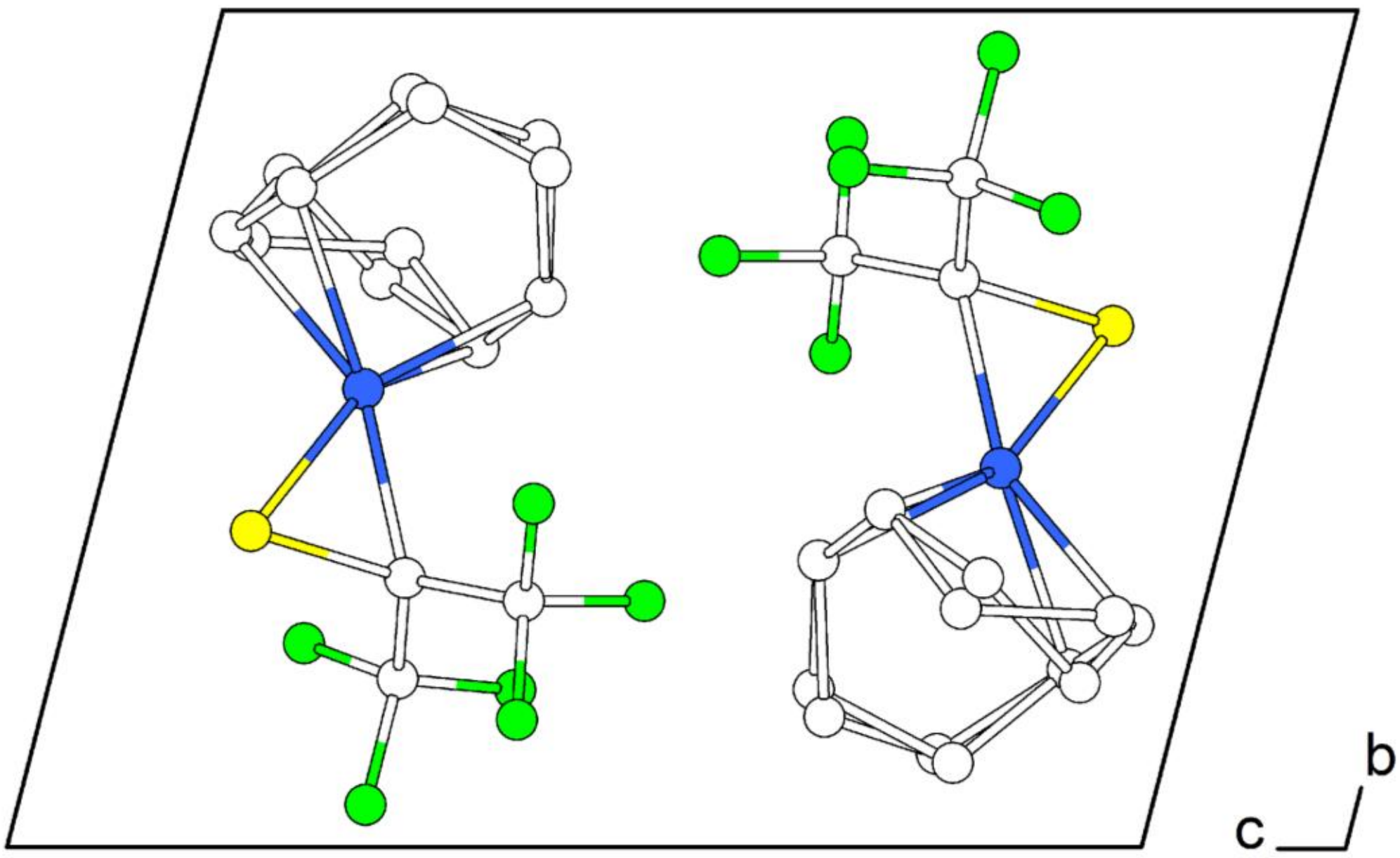


Figure S 47: Crystal structure of **3** (alpha) viewed along [100]. Atoms drawn with arbitrary radii. Hydrogen atoms are omitted for clarity. Colour code: S yellow, C white, F green, Ni dark blue.

### 2.3.4 Additional Pictures of 4

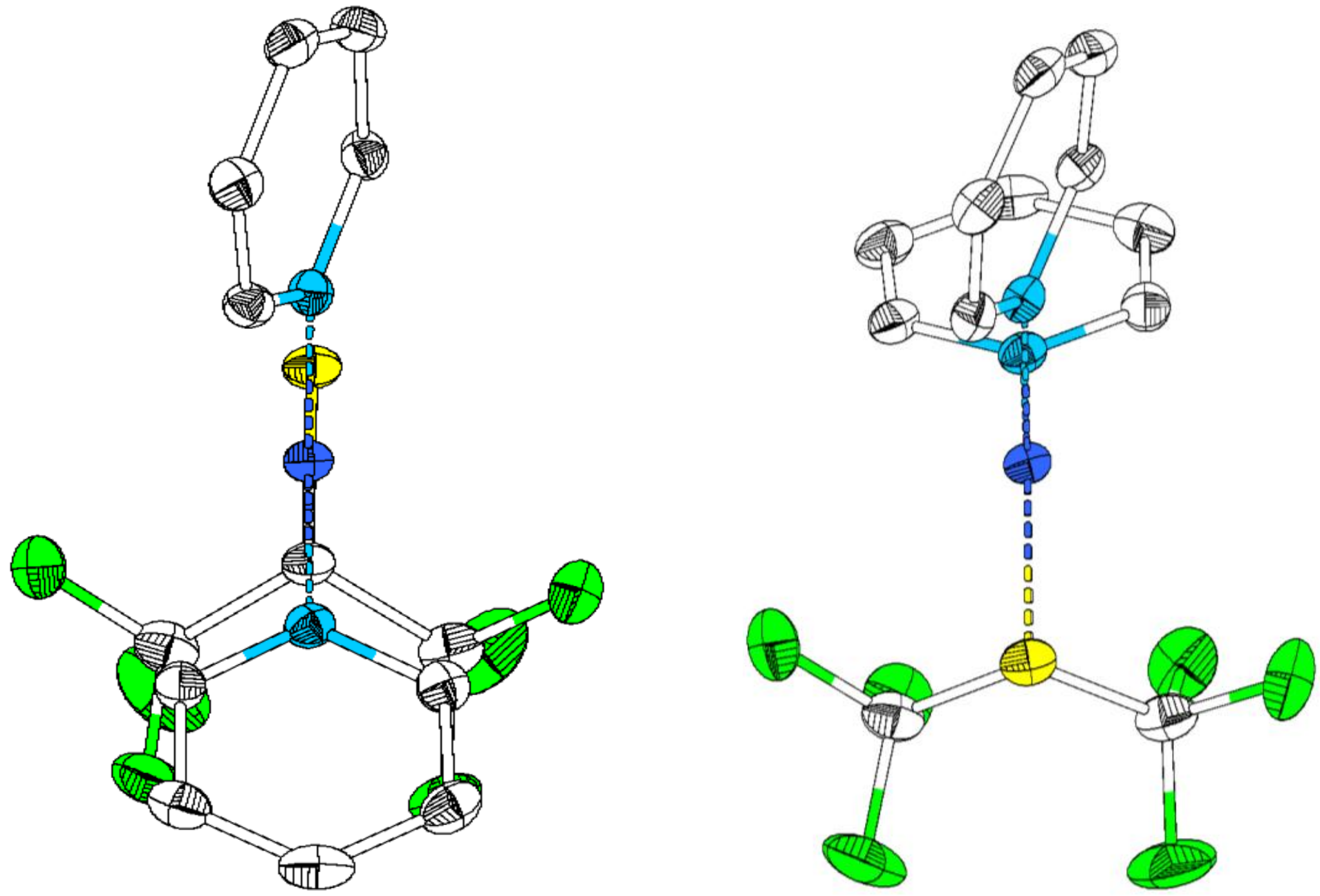

Figure S 48: Structure of **4** in the single crystal (left: front view, right: top view) drawn with 50% displacement ellipsoids at 100 K. Hydrogen atoms are omitted for clarity. Color code: C white, S yellow, F green, Ni dark blue, N light blue.

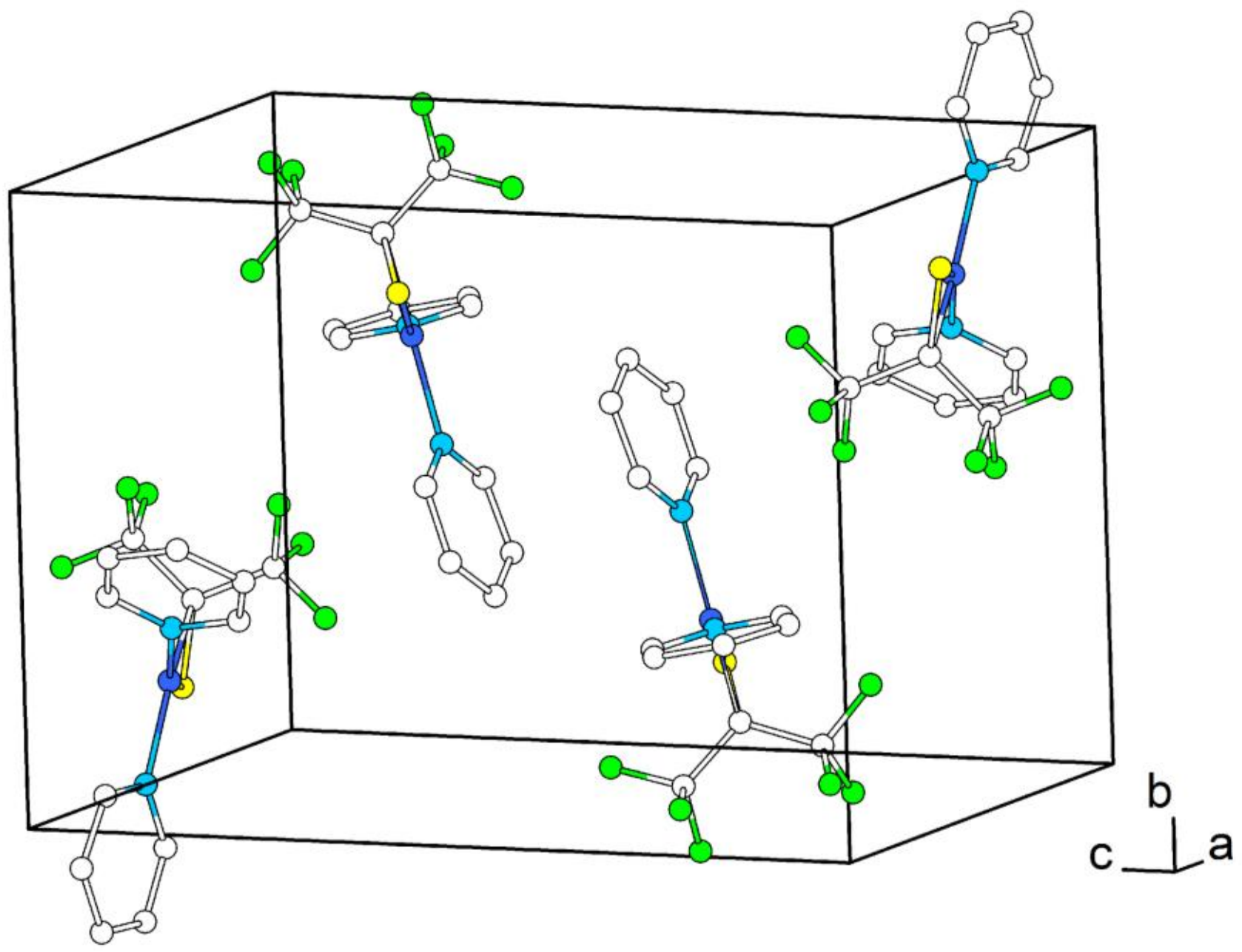


Figure S 49: Crystal structure of **4**. Atoms drawn with arbitrary radii. Hydrogen atoms are omitted for clarity. Colour code: S yellow, C white, F green, Ni dark blue, N light blue.

### 2.3.5 Additional Pictures of 5

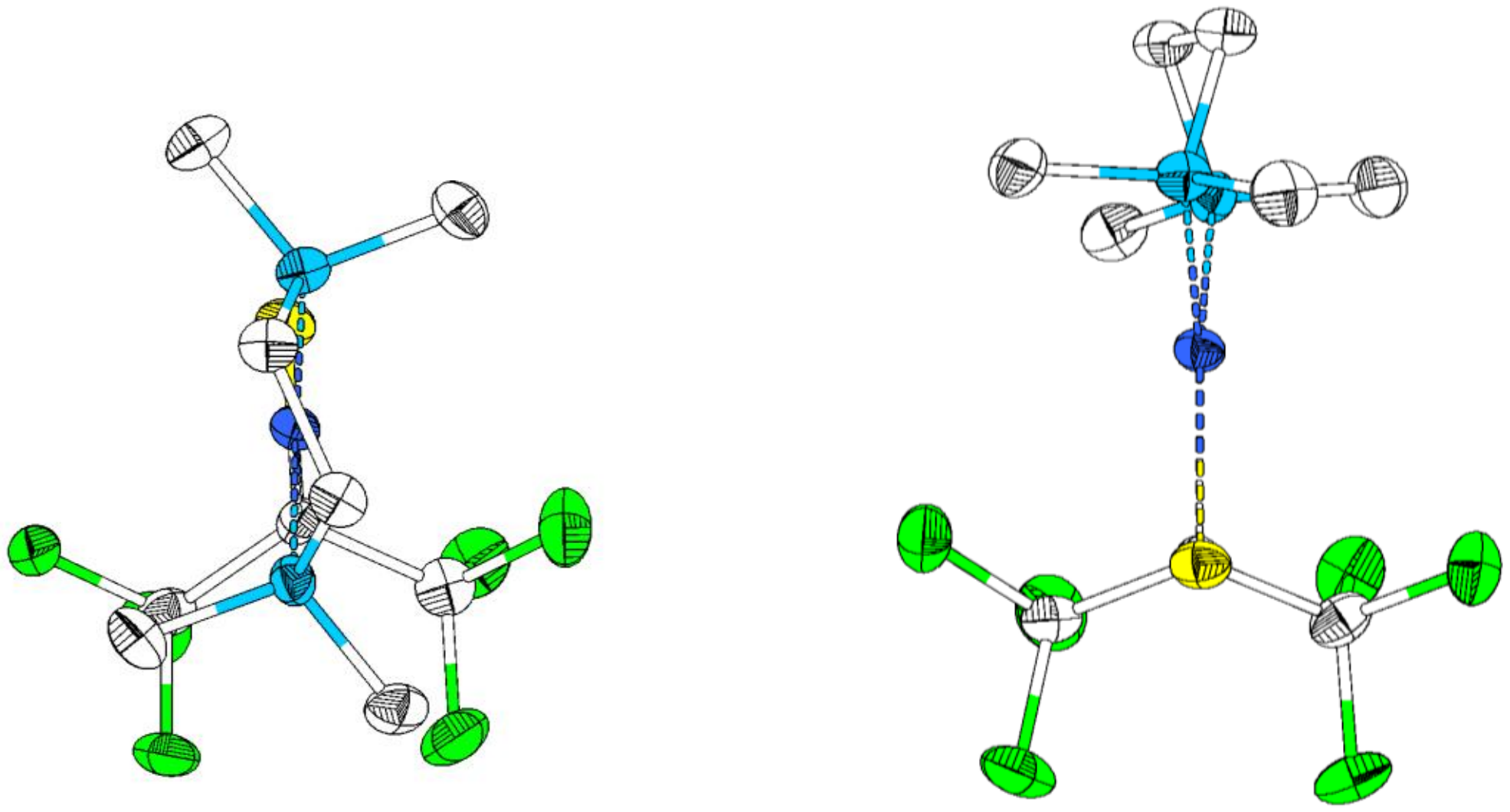

Figure S 50: Structure of **5** in the single crystal (left: front view, right: top view) drawn with 50% displacement ellipsoids at 100 K. Hydrogen atoms are omitted for clarity. Color code: C white, S yellow, F green, Ni dark blue, N light blue.

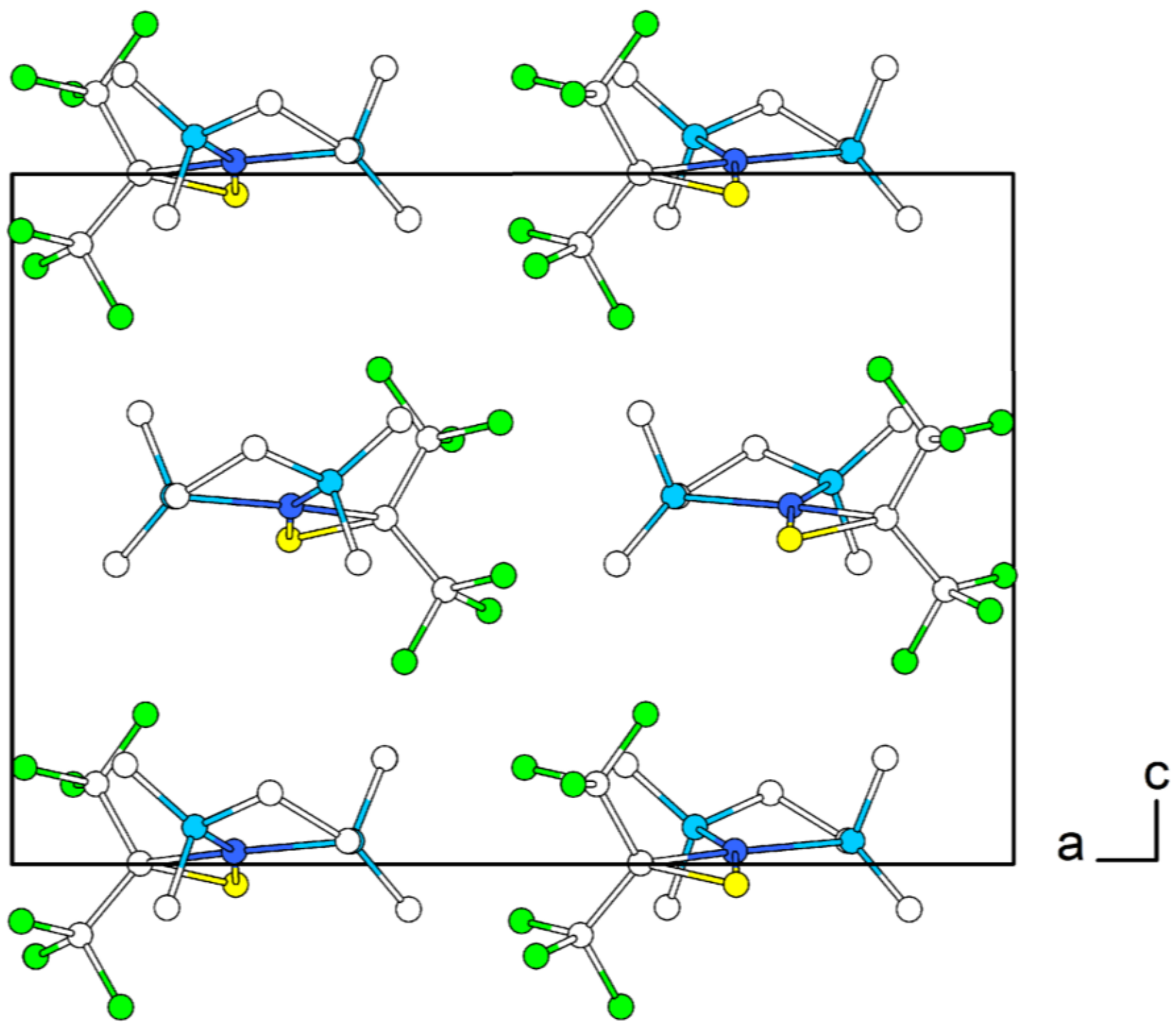


Figure S 51: Crystal structure of **4** viewed along [010]. Atoms drawn with arbitrary radii. Hydrogen atoms are omitted for clarity. Colour code: S yellow, C white, F green, Ni dark blue, N light blue.

## 3. Additional tables

Table S 2: Selected bond lengths (in Å) and angles (in °) of **1** and **2**.

| | **1** | **2** |
|---|---|---|
| C–S / Å | 1.583(5), 1.595(5) | 1.825(2), 1.830(2) |
| C–C / Å | 1.509(7)–1.524(6) | 1.542(3), 1.545(2) |
| C–C–S /° | 121.8(4)–123.4(4) | 111.7(1)–112.8(1) |
| C–C–C /° | 114.8(4), 115.0(4) | 111.8(1) |

Table S 3: Selected bond lengths (in Å) and angles (in °) of **3**–**5**.

| | **3-alpha** | **3-beta** | **4** | **5** |
|---|---|---|---|---|
| C–S / Å | 1.735(2) | 1.738(6) | 1.751(2) | 1.762(5) |
| C–Ni | 1.960(2) | 1.979(5) | 1.928(2) | 1.932(5) |
| S–Ni | 2.1372(7) | 2.133(2) | 2.1408(6) | 2.134(1) |
| C–S–Ni /° | 49.88(5) | 49.8(2) | 50.64(6) | 51.1(1) |
| C–C–S /° | 116.6(1), 117.2(1), | 116.9(4), 117.5(4) | 116.4(2), 117.2(2) | 114.6(3), 117.3(4) |

## 4. Computational details

Quantum chemical calculations were carried out with the TURBOMOLE program package version 7.8, using the PBE0 hybrid density functional method and polarized triple-ζ-valence Karlsruhe basis sets (DFT-PBE0/def2-TZVP).[14–18] Resolution-of-the-identity technique was used to speed up the calculations.[19,20] *m*4 integration grid was used for the numerical integration of the exchange-correlation functional. The studied molecular structures were fully optimized within the symmetry constraints of their point group. Harmonic frequency calculations were performed to check if the optimized structures were true local minima on the potential energy surface. When a one molecule of **1** was optimized in idealized point group $C_{2v}$, the frequency calculation showed two imaginary frequencies at 50*i* and 37*i* $cm^{-1}$. Distorting the structure along the first imaginary mode reduced the point group symmetry to $C_2$. Optimization in this point group resulted in a true local minimum. In the case of one molecule of **2**, the structure was optimized in point group $C_{2h}$. Frequency analysis showed the structure to be a true local minimum in this point group. The XYZ coordinates of the optimized structures are available below. Intrinsic bond orbital (IBO) analyses were performed with the Iboview program.[21,22] We calculated partial charges both from Intrinsic Atomic Orbital (IAO) analysis and Natural Population Analysis (NPA).[23]

### 4.1 Intrinsic Bond Orbitals for studied systems

The partial charges shown in the IBO plots are based on Intrinsic Atomic Orbitals (IAO). In all IBO pictures, the IBO isosurface contains 80% of the total density. Atom contributions to the IBOs are shown as percentages. If the percentages do not add up to 100%, some other atoms have small contributions of less than 2%. Partial charge trends obtained from Natural Population Analysis (NPA) and IAO analysis are in agreement. For example, in the case of **1**, the IAO (NPA) partial charges (*e*) are +0.16 (+0.28) for S, –0.19 (–0.33) for C1, +0.82 (+0.91) for C2, and –0.27 (–0.29) for F. In the case of **2**, the IAO (NPA) partial charges (*e*) are +0.21 (+0.38) for S, –0.24 (–0.47) for C1, +0.82 (+0.93) for C2, and –0.24 (–0.29) for F.

## Partial charges (*e*) for **1** ($C_2$)

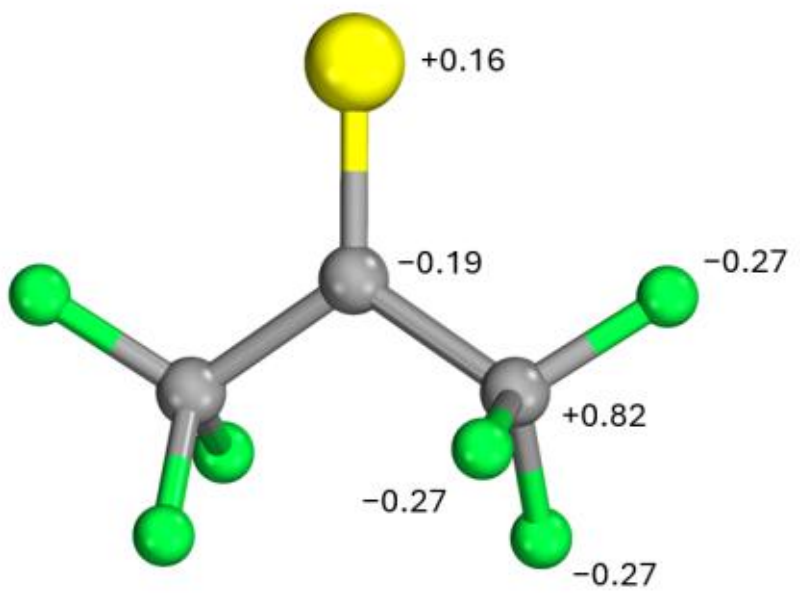


## IBOs for the C=S bond in **1**

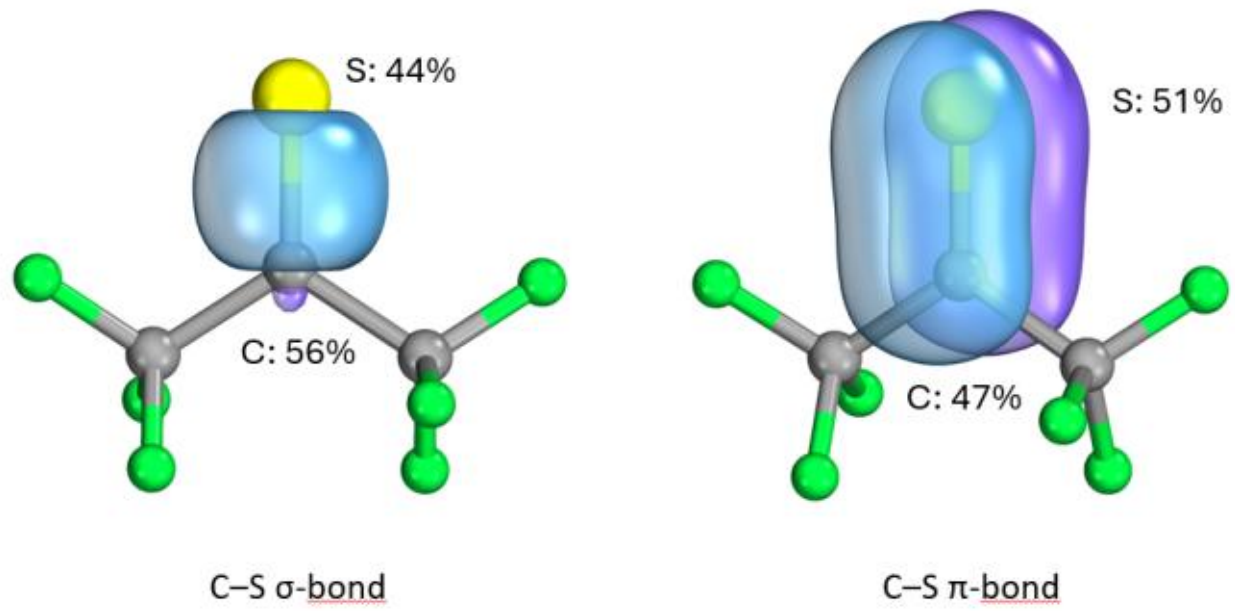


## IBOs for the S lone pairs in **1**

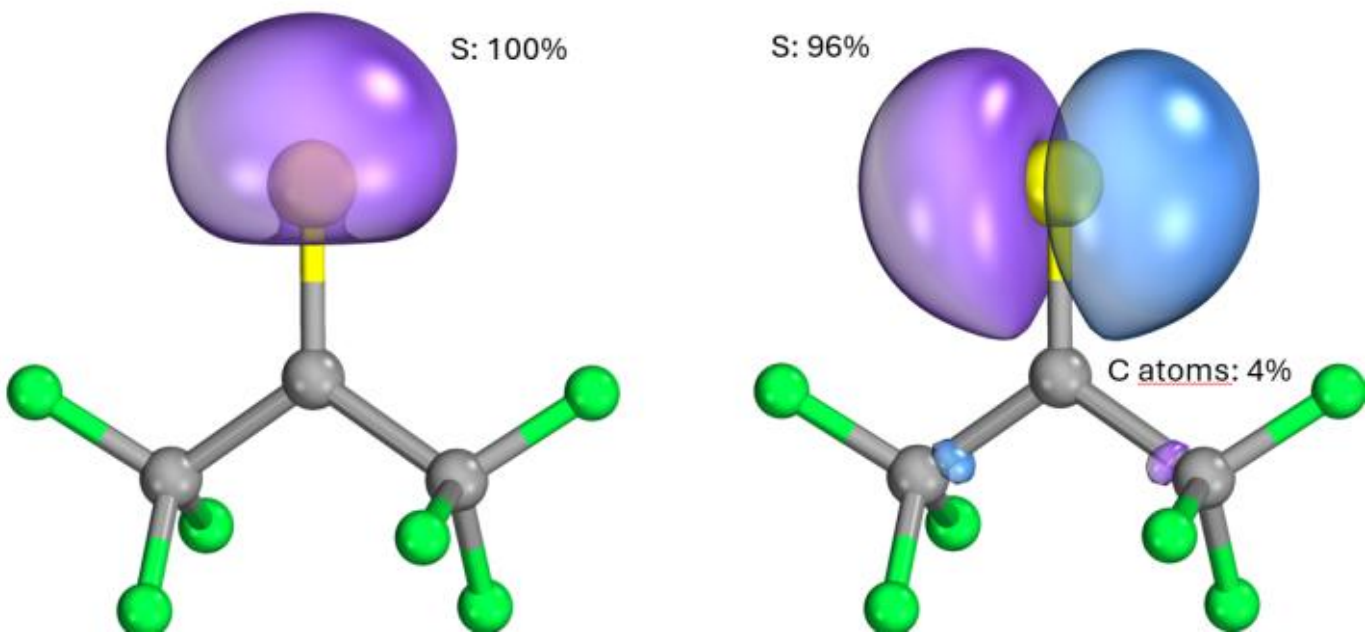


## IBOs for the C–C and C–F bonds in **1**

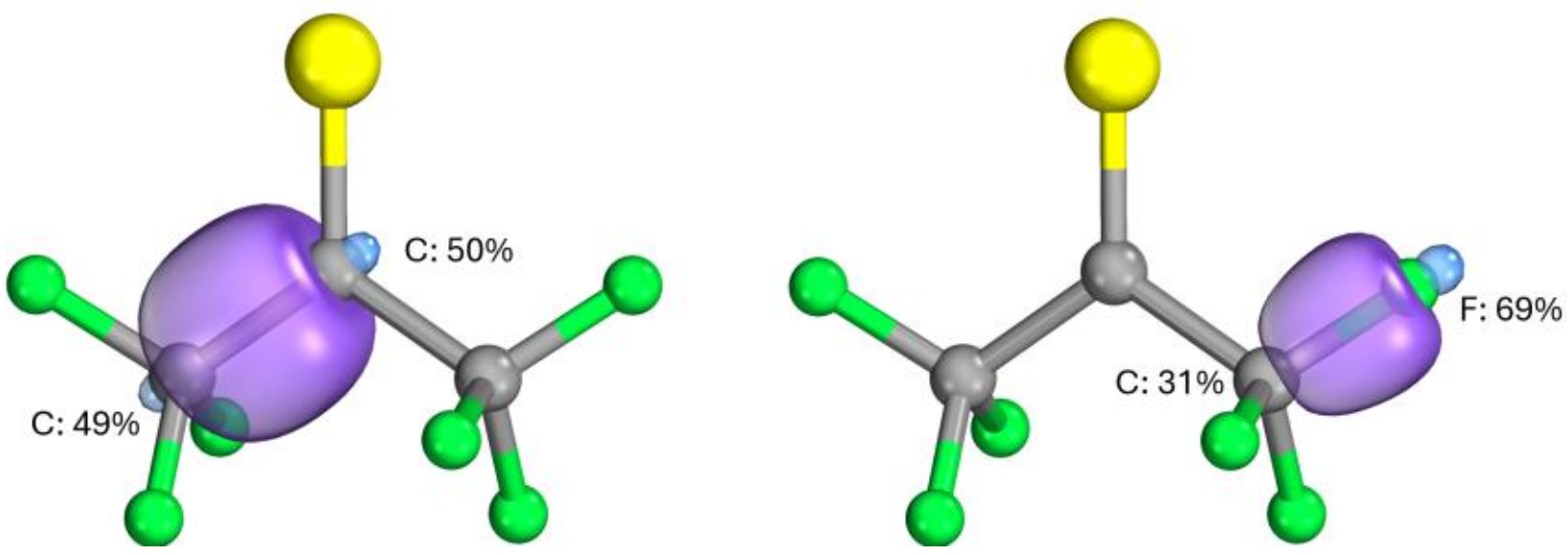


Figure S52. IAO partial charges and key IBOs for **1**.

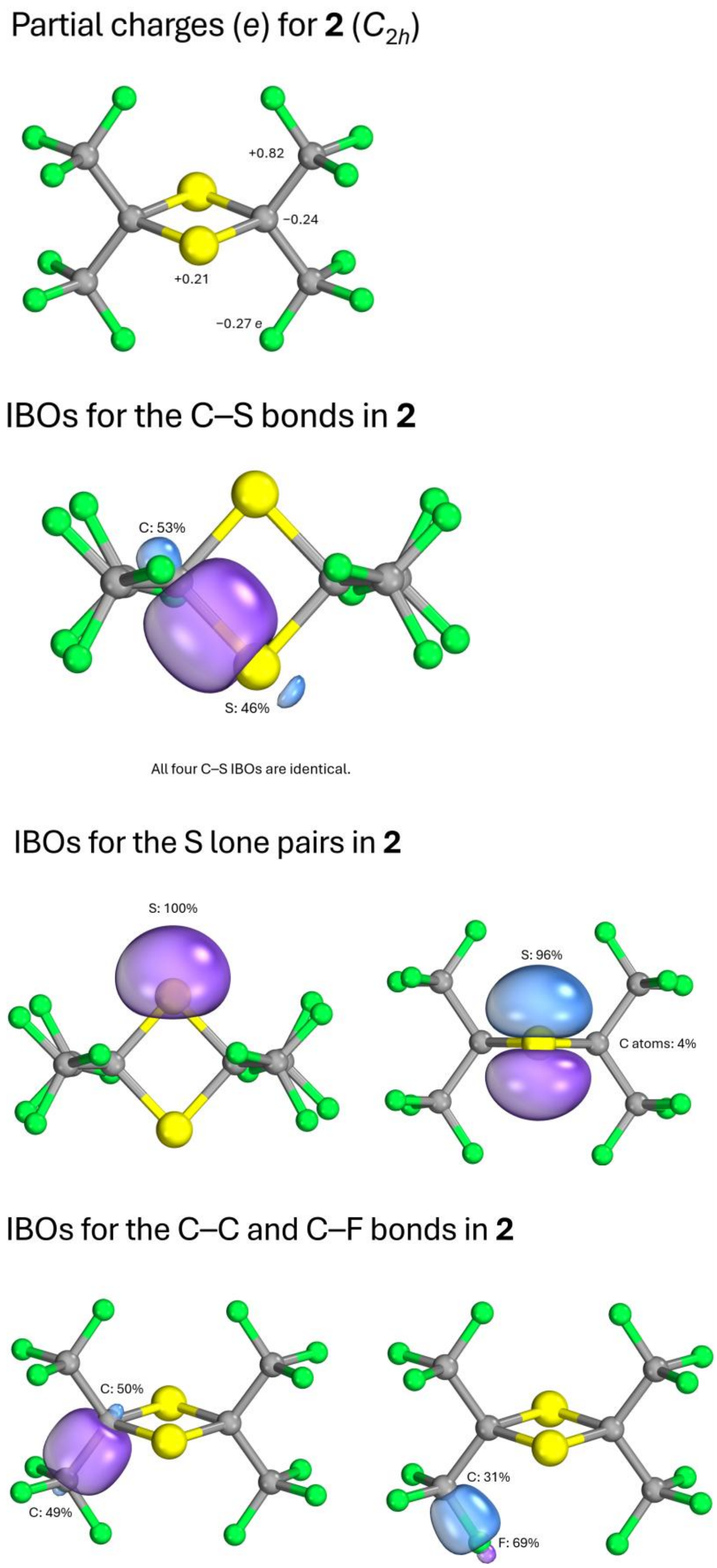


Figure S53. IAO partial charges and key IBOs for **2**.

## Partial charges (*e*) for **3** ($C_1$)

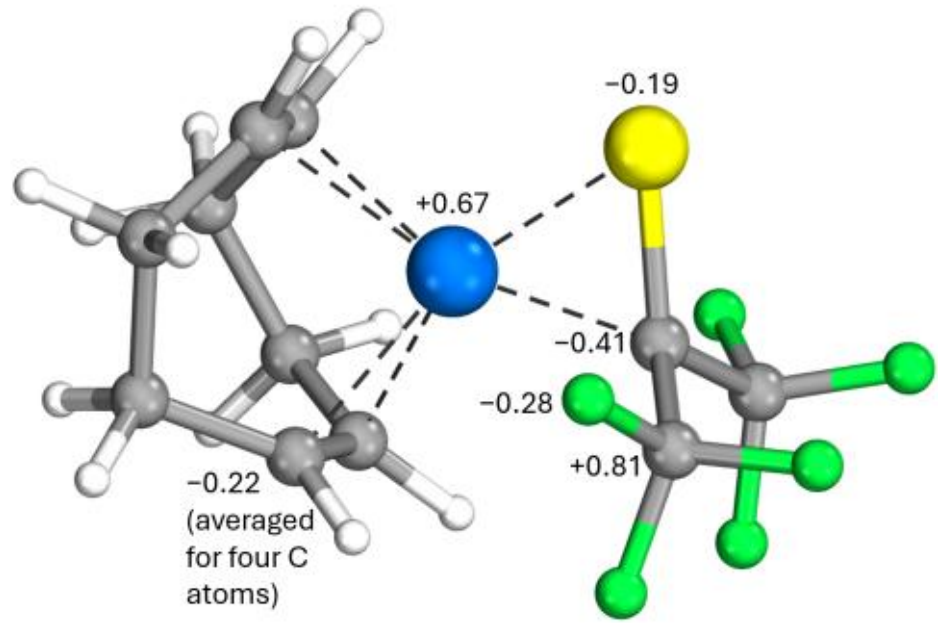


## IBO for the C–S bond in **3**

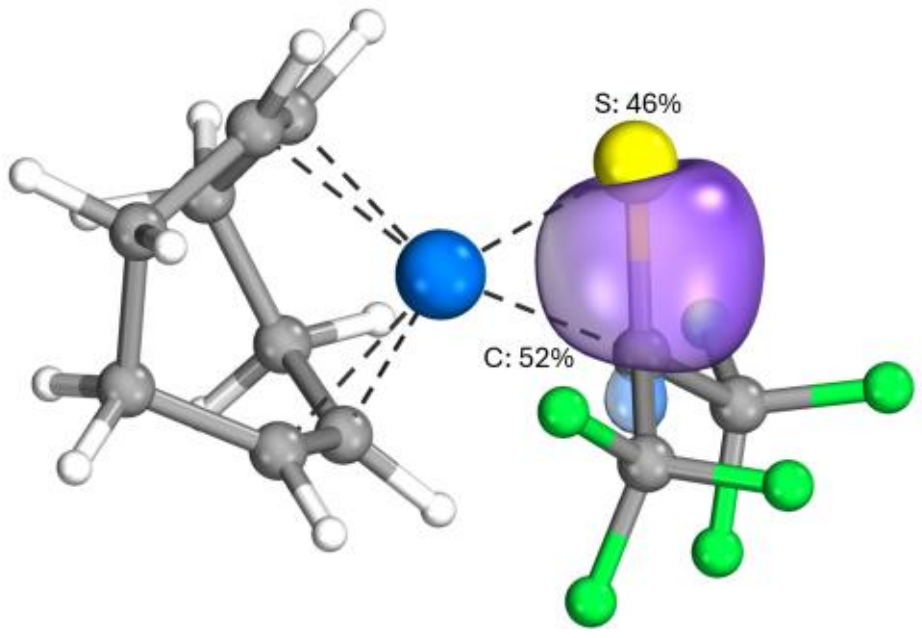


C–C and C–F IBOs are similar to **1** and **2** (not drawn here)

## IBOs for Ni–C and Ni–S bonds in **3**

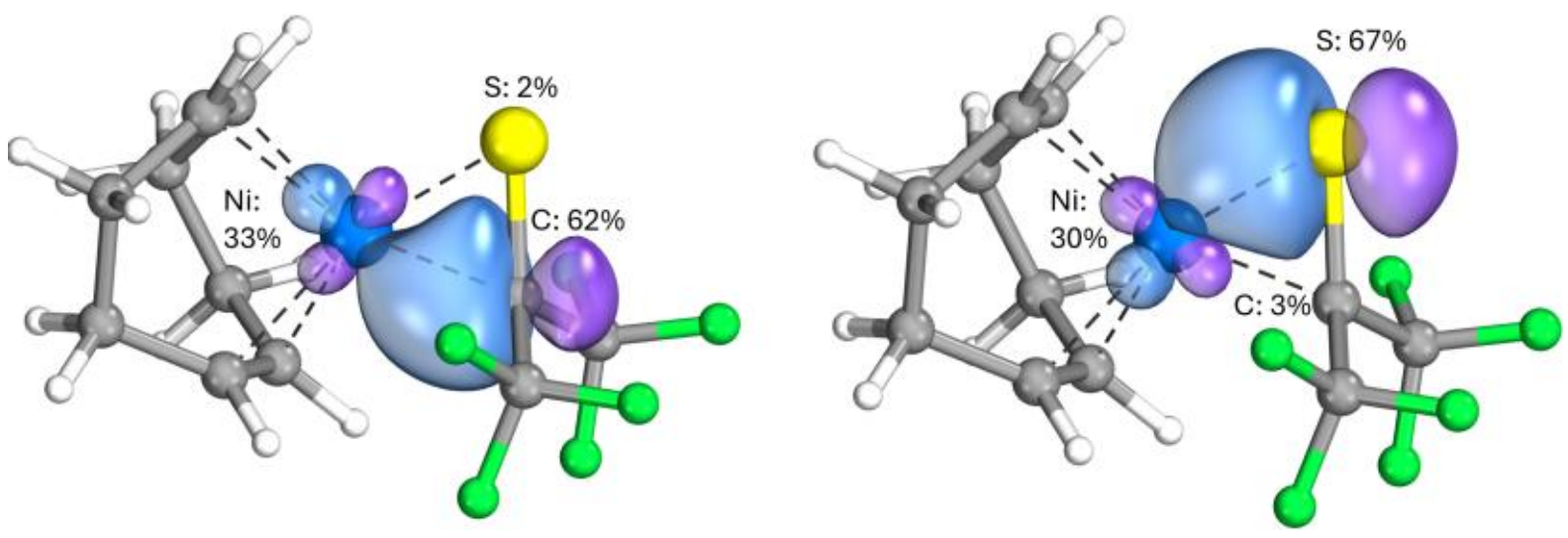


## Two IBOs for Ni coordination to C=C bonds in **3**

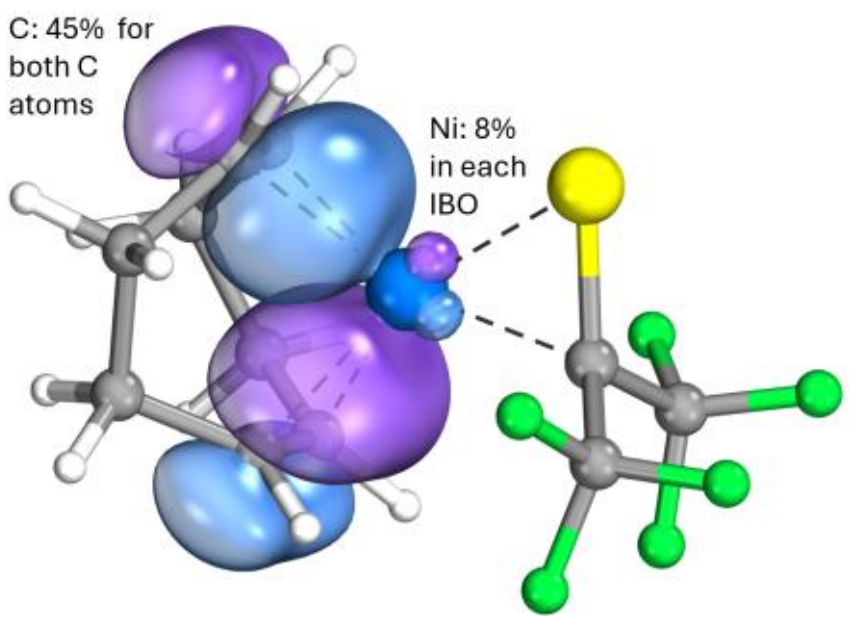


Figure S54. IAO partial charges and key IBOs for **3**.

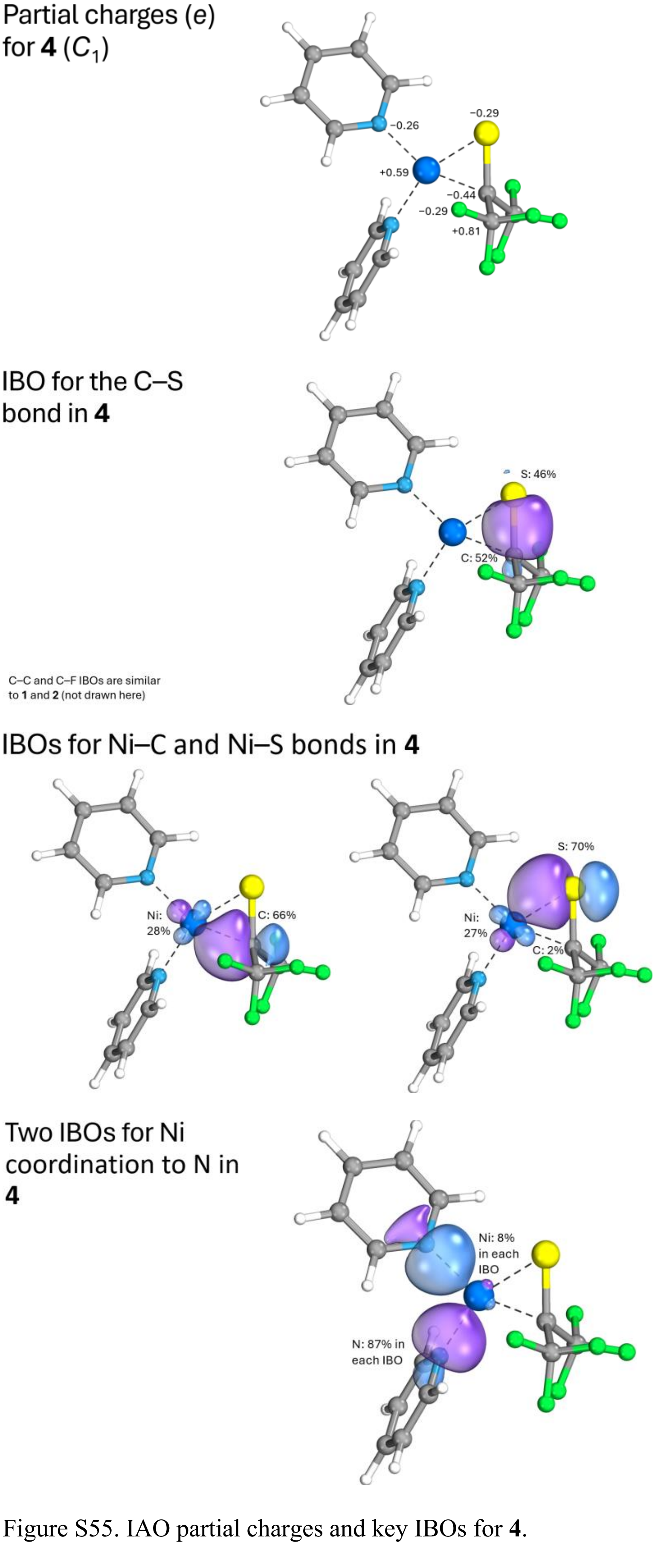


Figure S55. IAO partial charges and key IBOs for **4**.

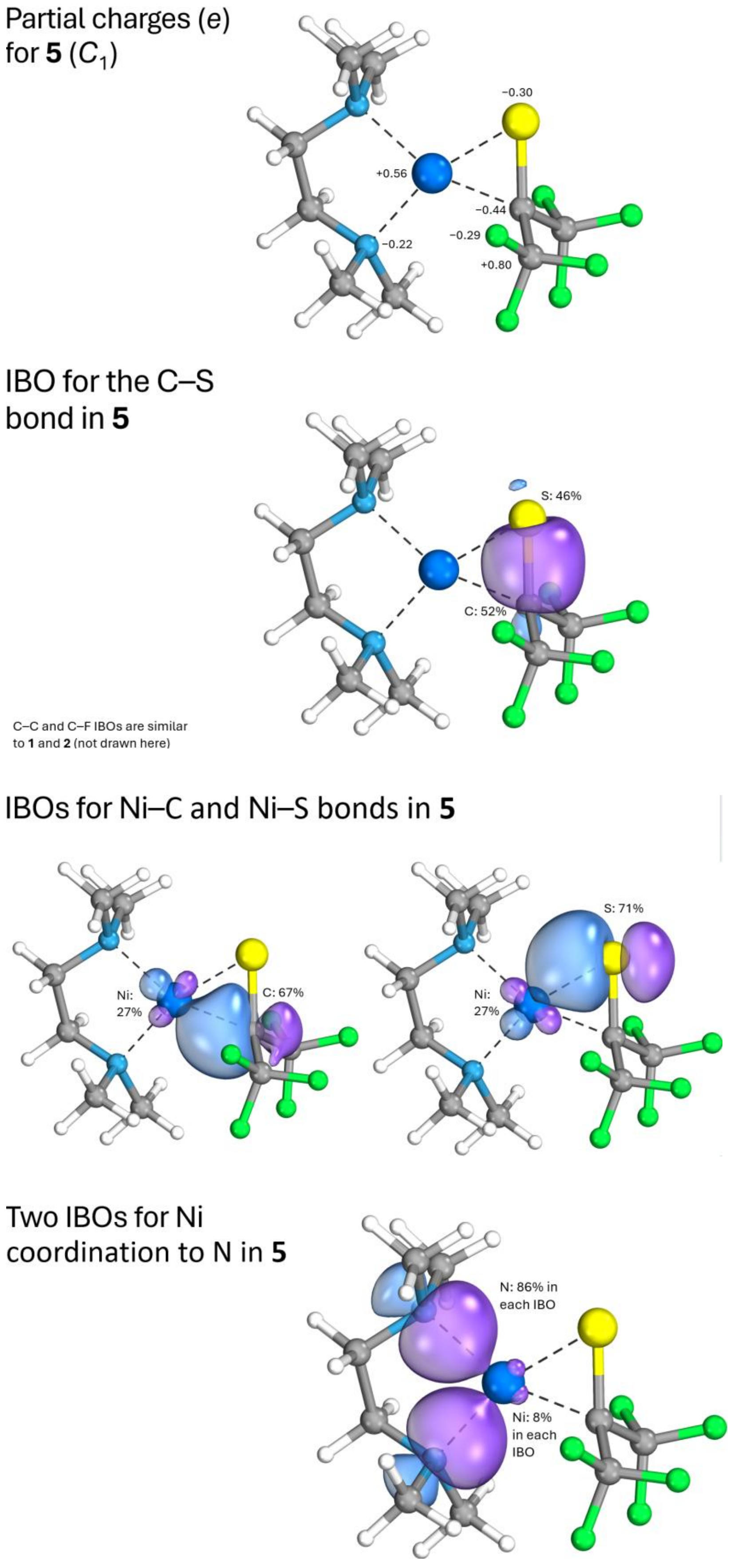


Figure S56. IAO partial charges and key IBOs for **5**.

## Partial charges ($e$) for thioacetone ($C_2$)

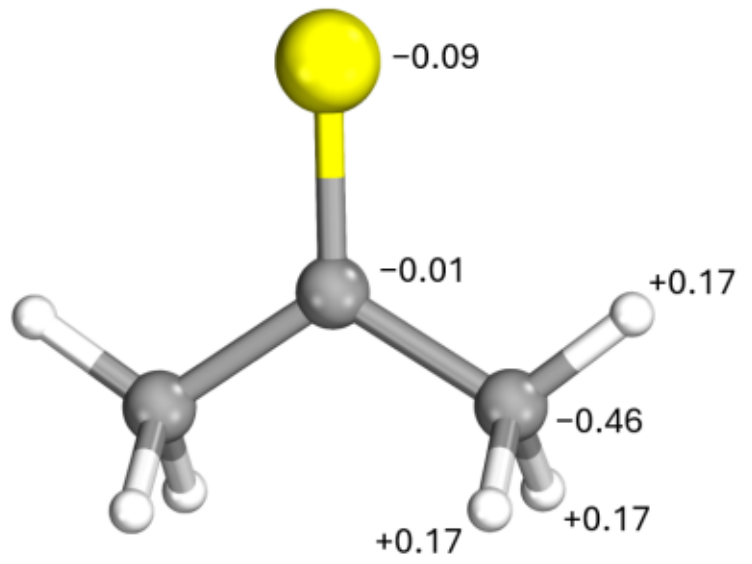


## IBOs for the C=S bond in thioacetone

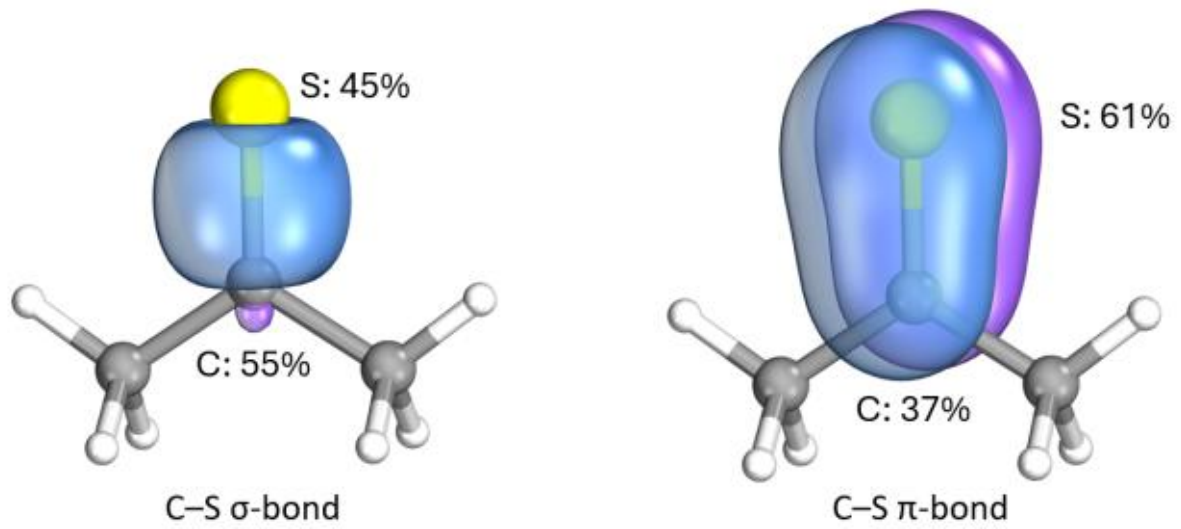


## IBOs for the S lone pairs in thioacetone

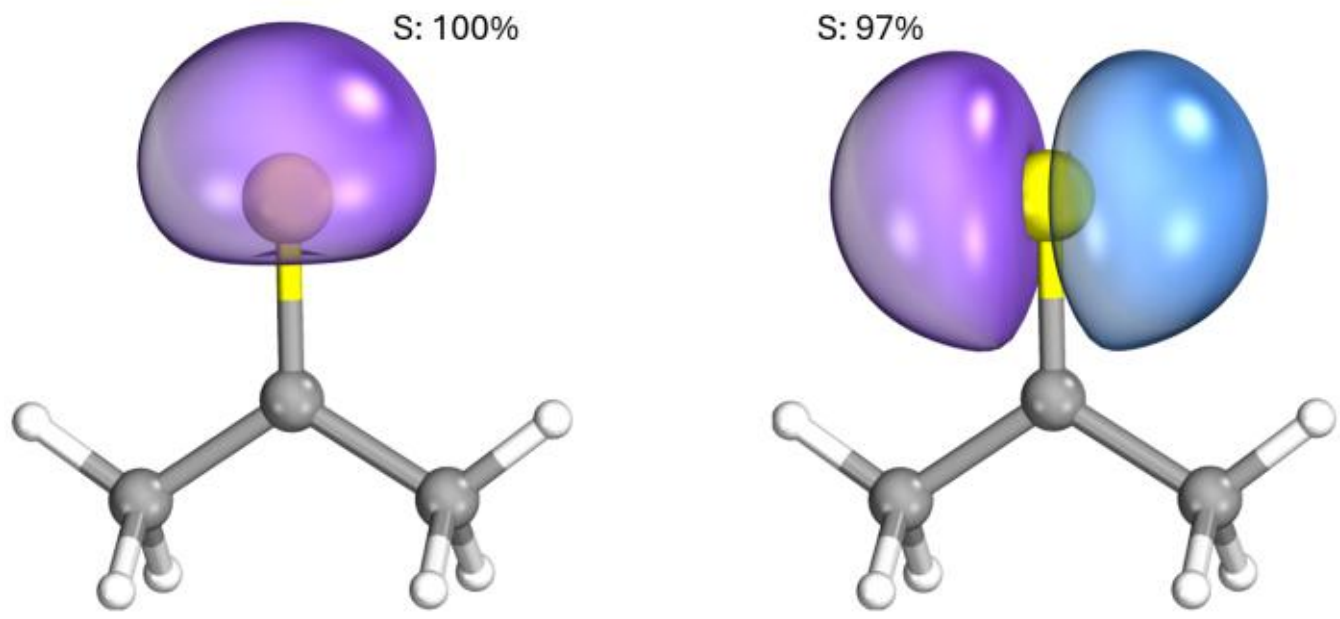


## IBOs for the C–C and C–H bonds in thioacetone

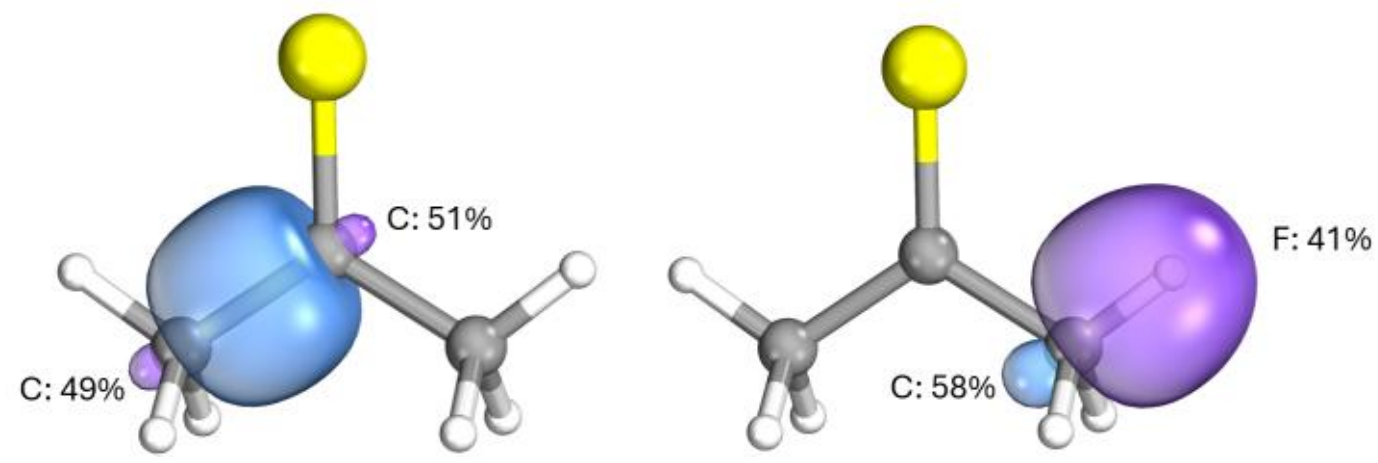


Figure S57. IAO partial charges and key IBOs for thioacetone $(CH_3)_2CS$.

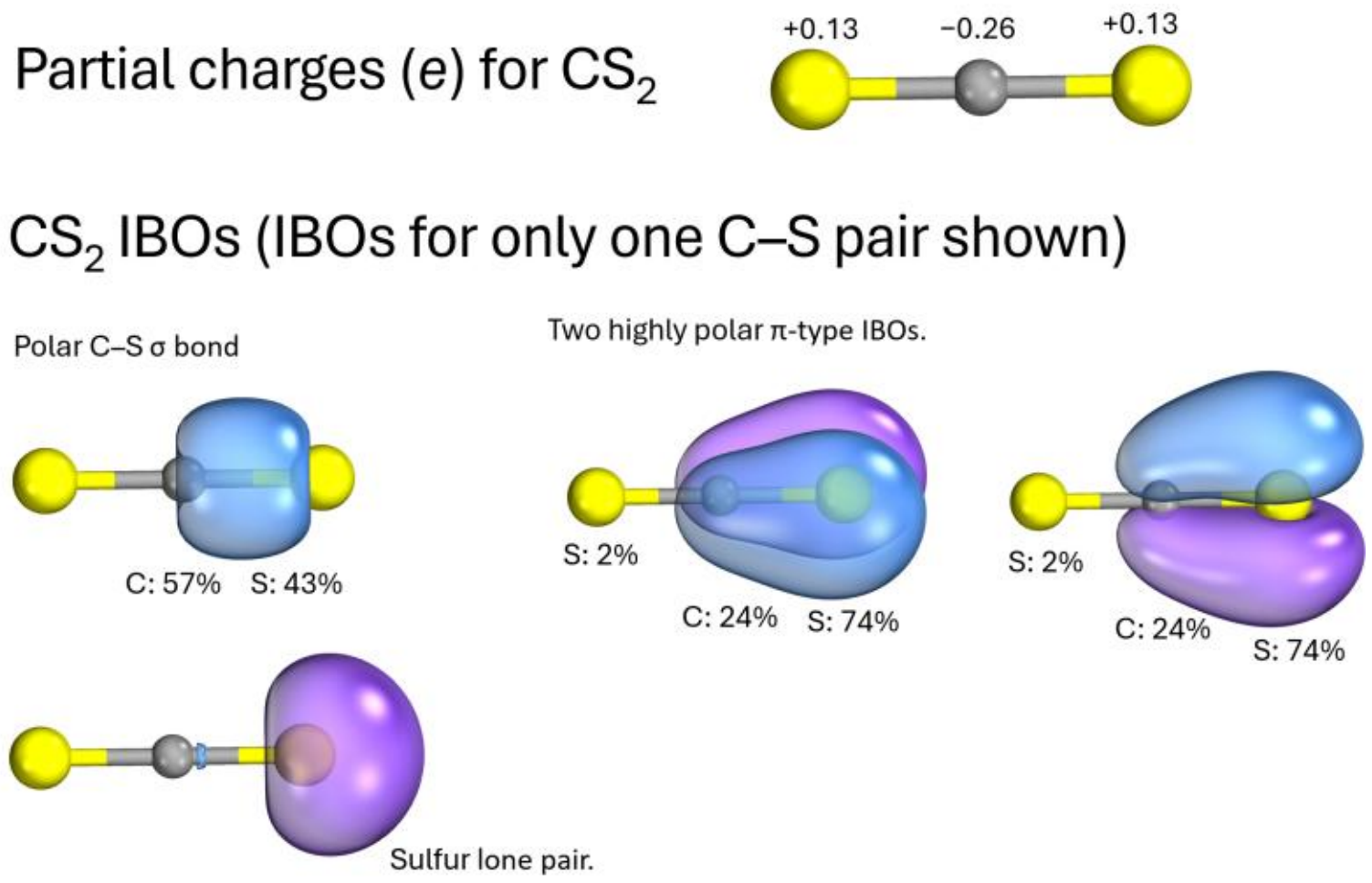


Figure S58. IAO partial charges and key IBOs for $CS_2$.

### 4.2 Optimized molecular structures in XYZ format

Molecule **1** (point group $C_2$)

```
10
Point group C2
C    -0.0000000   -0.0000000    0.6601123
S     0.0000000   -0.0000000    2.2511287
C    -0.1337360    1.2836624   -0.1673754
C     0.1337360   -1.2836624   -0.1673754
F     1.0714753    1.6626135   -0.5980284
F    -0.6488952    2.2744586    0.5409220
F    -0.9128530    1.0862280   -1.2311388
F     0.9128530   -1.0862280   -1.2311388
F    -1.0714753   -1.6626135   -0.5980284
F     0.6488952   -2.2744586    0.5409220
```

Molecule **2** (point group $C_{2h}$)

```
20
Point group C2h
S    -0.7527361   -1.1057047    0.0000000
S     0.7527361    1.1057047    0.0000000
F     1.8964097   -1.4262068    1.3793215
F    -1.8964097    1.4262068   -1.3793215
F    -1.8964097    1.4262068    1.3793215
F     1.8964097   -1.4262068   -1.3793215
F    -0.5265407    1.4628574    3.0316126
F     0.5265407   -1.4628574   -3.0316126
F    -1.8173627   -0.2422341    2.7271930
F     1.8173627    0.2422341   -2.7271930
F     1.8173627    0.2422341    2.7271930
F    -1.8173627   -0.2422341   -2.7271930
F     0.5265407   -1.4628574    3.0316126
F    -0.5265407    1.4628574   -3.0316126
C     1.0812445   -0.6805233    2.1070036
C    -1.0812445    0.6805233   -2.1070036
C    -1.0812445    0.6805233    2.1070036
C     1.0812445   -0.6805233   -2.1070036
C    -0.0000000    0.0000000    1.2324975
C    -0.0000000    0.0000000   -1.2324975
```

## $Ni(COD)_2$ (point group $D_2$)

41
Ni(COD2)

| | | | |
|---|---|---|---|
| Ni | 0.0000000 | 0.0000000 | 0.0000000 |
| H | 0.9680912 | 2.3439434 | -0.7407737 |
| C | 0.6076801 | 1.5133432 | -1.3413171 |
| C | 1.4520918 | 0.4324410 | -1.4793570 |
| C | -0.5714412 | 1.8275084 | -2.2204703 |
| C | 1.3941779 | -0.5898640 | -2.5923960 |
| H | 2.4000729 | 0.4935397 | -0.9556541 |
| C | -1.3941779 | 0.5898640 | -2.5923960 |
| H | -1.2138778 | 2.5305830 | -1.6811003 |
| H | -0.2476266 | 2.3575841 | -3.1279542 |
| C | 0.5714412 | -1.8275084 | -2.2204703 |
| H | 2.4127453 | -0.9001328 | -2.8416303 |
| H | 0.9999308 | -0.1257398 | -3.4987851 |
| C | -1.4520918 | -0.4324410 | -1.4793570 |
| H | -0.9999308 | 0.1257398 | -3.4987851 |
| H | -2.4127453 | 0.9001328 | -2.8416303 |
| C | -0.6076801 | -1.5133432 | -1.3413171 |
| H | 0.2476266 | -2.3575841 | -3.1279542 |
| H | 1.2138778 | -2.5305830 | -1.6811003 |
| H | -2.4000729 | -0.4935397 | -0.9556541 |
| H | -0.9680912 | -2.3439434 | -0.7407737 |
| H | 2.4127453 | 0.9001328 | 2.8416303 |
| C | 1.3941779 | 0.5898640 | 2.5923960 |
| C | 1.4520918 | -0.4324410 | 1.4793570 |
| C | 0.5714412 | 1.8275084 | 2.2204703 |
| H | 0.9999308 | 0.1257398 | 3.4987851 |
| C | 0.6076801 | -1.5133432 | 1.3413171 |
| H | 2.4000729 | -0.4935397 | 0.9556541 |
| C | -0.6076801 | 1.5133432 | 1.3413171 |
| H | 0.2476266 | 2.3575841 | 3.1279542 |
| H | 1.2138778 | 2.5305830 | 1.6811003 |
| C | -0.5714412 | -1.8275084 | 2.2204703 |
| H | 0.9680912 | -2.3439434 | 0.7407737 |
| C | -1.4520918 | 0.4324410 | 1.4793570 |
| H | -0.9680912 | 2.3439434 | 0.7407737 |
| C | -1.3941779 | -0.5898640 | 2.5923960 |
| H | -1.2138778 | -2.5305830 | 1.6811003 |
| H | -0.2476266 | -2.3575841 | 3.1279542 |
| H | -2.4000729 | 0.4935397 | 0.9556541 |
| H | -0.9999308 | -0.1257398 | 3.4987851 |
| H | -2.4127453 | -0.9001328 | 2.8416303 |

## **3** (point group $C_1$)

31
Molecule 3

| | | | |
|---|---|---|---|
| Ni | 0.3860754 | 0.6066633 | 0.1211104 |
| H | 0.4640461 | -1.3761770 | -1.4998443 |
| C | -0.3915030 | -1.0175633 | -0.9347615 |
| C | -0.4224734 | -1.3169130 | 0.4025402 |
| C | -1.5116603 | -0.4481068 | -1.7566289 |
| C | -1.6262890 | -1.2200393 | 1.3047833 |
| H | 0.4310481 | -1.8588505 | 0.7997433 |
| C | -2.3648637 | 0.5917876 | -1.0145314 |
| H | -1.0664812 | 0.0185198 | -2.6390538 |
| H | -2.1506345 | -1.2568214 | -2.1345277 |
| C | -1.7133068 | 0.1278892 | 2.0366144 |
| H | -1.5676835 | -2.0161890 | 2.0503618 |
| H | -2.5351672 | -1.4086524 | 0.7325760 |
| C | -1.5417073 | 1.4897767 | -0.1292035 |
| H | -3.1441687 | 0.1055845 | -0.4273272 |
| H | -2.8819877 | 1.2093669 | -1.7525500 |
| C | -1.2974518 | 1.3039205 | 1.2013289 |
| H | -2.7290044 | 0.2816044 | 2.4232720 |
| H | -1.0604376 | 0.1001842 | 2.9126054 |
| H | -1.2577048 | 2.4505141 | -0.5505257 |
| H | -0.8717166 | 2.1391996 | 1.7496838 |
| F | 2.8922424 | -1.6783476 | 0.6249224 |

| | | | |
|---|---|---|---|
| C | 3.0652263 | -0.3653495 | 0.8749779 |
| C | 2.3231464 | 0.5071649 | -0.1189149 |
| F | 4.3876970 | -0.1498980 | 0.8678095 |
| F | 2.6401657 | -0.1625940 | 2.1249853 |
| S | 1.8741945 | 2.0978373 | 0.3715603 |
| C | 2.8032357 | 0.3518791 | -1.5497114 |
| F | 3.9991253 | 0.9143061 | -1.7601150 |
| F | 1.9515641 | 0.9173251 | -2.4167672 |
| F | 2.9164745 | -0.9380214 | -1.9144123 |

## **4** (point group $C_1$)

33
Molecule 4

| | | | |
|---|---|---|---|
| Ni | 0.8384997 | 0.0613897 | 0.4820787 |
| F | 3.0561496 | -0.6190920 | -2.3068430 |
| C | 3.2940609 | -0.6588134 | -0.9785483 |
| C | 2.5821353 | 0.4465668 | -0.2338694 |
| F | 4.6259638 | -0.6353371 | -0.8363487 |
| F | 2.8895540 | -1.8748875 | -0.5692465 |
| S | 2.6765173 | 0.3743991 | 1.5025069 |
| C | 2.7157450 | 1.7990466 | -0.8989446 |
| F | 3.9913863 | 2.1532441 | -1.1345134 |
| F | 2.1739539 | 2.7762714 | -0.1639403 |
| F | 2.0900254 | 1.8419358 | -2.0944414 |
| N | -0.5184956 | -0.1982631 | 1.8572705 |
| C | -1.4546625 | -1.1482068 | 1.7721240 |
| C | -0.4969806 | 0.5695302 | 2.9539768 |
| C | -2.3912172 | -1.3702722 | 2.7644933 |
| H | -1.4425108 | -1.7506996 | 0.8717819 |
| C | -1.4045815 | 0.4191475 | 3.9860828 |
| H | 0.2880830 | 1.3149098 | 2.9891710 |
| C | -2.3697694 | -0.5700887 | 3.8948099 |
| H | -3.1213255 | -2.1606633 | 2.6453345 |
| H | -1.3419418 | 1.0690399 | 4.8495306 |
| H | -3.0901629 | -0.7167968 | 4.6909336 |
| N | -0.3184300 | -0.0632529 | -1.0691711 |
| C | -0.1640193 | -1.0209903 | -1.9898709 |
| C | -1.2652999 | 0.8593236 | -1.2704935 |
| C | -0.9474171 | -1.0940261 | -3.1269474 |
| H | 0.6243215 | -1.7395314 | -1.8049658 |
| C | -2.0845901 | 0.8606119 | -2.3832618 |
| H | -1.3546016 | 1.6242942 | -0.5085200 |
| C | -1.9257525 | -0.1359485 | -3.3321724 |
| H | -0.7763916 | -1.8892778 | -3.8410832 |
| H | -2.8289897 | 1.6379882 | -2.4988328 |
| H | -2.5492559 | -0.1615515 | -4.2180799 |

## **5** (point group $C_1$)

35
Molecule 5

| | | | |
|---|---|---|---|
| Ni | 0.3920005 | 0.4385933 | -0.3464670 |
| F | 2.4863620 | 0.6384320 | 2.4005173 |
| C | 2.6178260 | 1.4336280 | 1.3080219 |
| C | 2.2648566 | 0.7053933 | 0.0332911 |
| F | 3.8798761 | 1.8900017 | 1.3402816 |
| F | 1.8200550 | 2.4836232 | 1.5271749 |
| S | 1.7951482 | 1.6821103 | -1.3368944 |
| C | 3.2087168 | -0.4327456 | -0.2852664 |
| F | 4.4005030 | -0.0296224 | -0.7476405 |
| F | 2.6978880 | -1.2551289 | -1.2181843 |
| F | 3.4694762 | -1.2076312 | 0.7895956 |
| N | -0.4691272 | -0.8193699 | 0.9606138 |
| N | -1.3551373 | 0.5497633 | -1.3196277 |
| C | 0.3446603 | -1.9881717 | 1.3152263 |
| H | 0.6561751 | -2.5084643 | 0.4101021 |
| H | 1.2348712 | -1.6615593 | 1.8472704 |
| H | -0.2265076 | -2.6766929 | 1.9530255 |
| C | -0.7975166 | -0.0828480 | 2.1889566 |

| | | | |
|---|---|---|---|
| H | -1.3864730 | 0.8044815 | 1.9609118 |
| H | -1.3652839 | -0.7218473 | 2.8797154 |
| H | 0.1231627 | 0.2373123 | 2.6713379 |
| C | -1.1991865 | -0.1832938 | -2.5828656 |
| H | -0.4151279 | 0.2882156 | -3.1728461 |
| H | -0.8985743 | -1.2128697 | -2.3925829 |
| H | -2.1413457 | -0.1828188 | -3.1487212 |
| C | -1.7698734 | 1.9253885 | -1.6123892 |
| H | -1.0286849 | 2.3945320 | -2.2565514 |
| H | -2.7483446 | 1.9408592 | -2.1118410 |
| H | -1.8266196 | 2.4979685 | -0.6871103 |
| C | -1.6954882 | -1.2644564 | 0.2774170 |
| C | -2.3418434 | -0.1075265 | -0.4481455 |
| H | -3.2136133 | -0.4493173 | -1.0222333 |
| H | -2.7010001 | 0.6337359 | 0.2680160 |
| H | -1.4121950 | -2.0442143 | -0.4325933 |
| H | -2.3996351 | -1.7154602 | 0.9904851 |

# 6. References


[1] W. L. F. Armarego, C. L. L. Chai, *Purification of Organic Chemicals*, **2009**.

[2] David C. England, “Fluoroketenes. 11.1 Synthesis and Chemistry of a Perfluoroacylketene and Related Compounds Containing a Perfluoroisopropyl Sulfide Group” *J. Org. Chem.* **1981**, *46*, 153.

[3] T. F. C. Cruz, P. S. Lopes, P. T. Gomes, “Allylnickel(II) complexes of bulky 5-substituted-2-iminopyrrolyl ligands” *Polyhedron* **2021**, *207*, 115357.

[4] W. J. Middleton, E. G. Howard, W. H. Sharkey, “PERFLUOROTHIOCARBONYL COMPOUNDS” *J. Am. Chem. Soc.* **1961**, *83*, 2589–2590.

[5] D. J. Clouthier, D.-L. Joo, “The spectroscopy of hexafluorothioacetone, a blue gas” *J. Chem. Phys.* **1997**, *106*, 7479–7490.

[6] J. Browning, C. S. Cundy, M. Green, F. G. A. Stone, “Hexafluoroacetone and hexafluorothioacetone nickel complexes” *J. Chem. Soc. Inorg. Phys. Theor.* **1969**, 20–23.

[7] APEX3 V2019.11-2, Bruker AXS Inc., Madison, Wisconsin, USA, **2019**.

[8] G. M. Sheldrick, “SHELXT – Integrated space-group and crystal-structure determination” *Acta Crystallogr. Sect. Found. Adv.* **2015**, *71*, 3–8.

[9] G. M. Sheldrick, “Crystal structure refinement with *SHELXL*” *Acta Crystallogr. Sect. C Struct. Chem.* **2015**, *71*, 3–8.

[10] O. V. Dolomanov, L. J. Bourhis, R. J. Gildea, J. A. K. Howard, H. Puschmann, “*OLEX2*: a complete structure solution, refinement and analysis program” *J. Appl. Crystallogr.* **2009**, *42*, 339–341.

[11] K. Brandenburg, H. Putz, *DIAMOND, Program for X-Ray Structure Analysis*, Crystal Impact GbR, Bonn, Germany, **1999**.

[12] M. A. Spackman, D. Jayatilaka, “Hirshfeld surface analysis” *CrystEngComm* **2009**, *11*, 19–32.

[13] P. R. Spackman, M. J. Turner, J. J. McKinnon, S. K. Wolff, D. J. Grimwood, D. Jayatilaka, M. A. Spackman, “*CrystalExplorer* : a program for Hirshfeld surface analysis, visualization and quantitative analysis of molecular crystals” *J. Appl. Crystallogr.* **2021**, *54*, 1006–1011.

[14], “TURBOMOLE, Version 7.9 2024; a development of University of Karlsruhe and Forschungszentrum Karlsruhe GmbH 1989-2007, TURBOMOLE GmbH since 2007, available via https://www.turbomole.org.,” **2024**.

[15] Y. J. Franzke, C. Holzer, J. H. Andersen, T. Begušić, F. Bruder, S. Coriani, F. Della Sala, E. Fabiano, D. A. Fedotov, S. Fürst, S. Gillhuber, R. Grotjahn, M. Kaupp, M. Kehry, M. Krstić, F. Mack, S. Majumdar, B. D. Nguyen, S. M. Parker, F. Pauly, A. Pausch, E. Perlt, G. S. Phun, A. Rajabi, D. Rappoport, B. Samal, T. Schrader, M. Sharma, E. Tapavicza, R. S. Treß, V. Voora, A. Wodyński, J. M. Yu, B. Zerulla, F. Furche, C. Hättig, M. Sierka, D. P. Tew, F. Weigend, “TURBOMOLE: Today and Tomorrow” *J. Chem. Theory Comput.* **2023**, *19*, 6859–6890.

[16] J. P. Perdew, K. Burke, M. Ernzerhof, “Generalized Gradient Approximation Made Simple” *Phys. Rev. Lett.* **1996**, *77*, 3865–3868.

[17] C. Adamo, V. Barone, “Toward reliable density functional methods without adjustable parameters: The PBE0 model” *J. Chem. Phys.* **1999**, *110*, 6158–6170.

[18] F. Weigend, R. Ahlrichs, "Balanced basis sets of split valence, triple zeta valence and quadruple zeta valence quality for H to Rn: Design and assessment of accuracy" *Phys. Chem. Chem. Phys.* **2005**, *7*, 3297.
[19] K. Eichkorn, O. Treutler, H. Öhm, M. Häser, R. Ahlrichs, "Auxiliary basis sets to approximate Coulomb potentials" *Chem. Phys. Lett.* **1995**, *240*, 283–290.
[20] F. Weigend, "Accurate Coulomb-fitting basis sets for H to Rn" *Phys. Chem. Chem. Phys.* **2006**, *8*, 1057.
[21] G. Knizia, "Intrinsic Atomic Orbitals: An Unbiased Bridge between Quantum Theory and Chemical Concepts" *J. Chem. Theory Comput.* **2013**, *9*, 4834–4843.
[22] G. Knizia, J. E. M. N. Klein, "Electron Flow in Reaction Mechanisms—Revealed from First Principles" *Angew. Chem. Int. Ed.* **2015**, *54*, 5518–5522.
[23] A. E. Reed, R. B. Weinstock, F. Weinhold, "Natural population analysis" *J. Chem. Phys.* **1985**, *83*, 735–746.
